# Pericytes change function depending on glioblastoma vicinity: emphasis on immune regulation

**DOI:** 10.1002/1878-0261.70095

**Published:** 2025-07-17

**Authors:** Carolina Buizza, Robert Carlsson, Coralie Gamper, Gayatri Chitale, Johan Bengzon, Gesine Paul

**Affiliations:** ^1^ Department of Clinical Sciences, Translational Neurology Group Lund University Sweden; ^2^ Department of Neurosurgery Scania University Hospital Lund Sweden; ^3^ Department of Neurology Scania University Hospital Lund Sweden; ^4^ Wallenberg Centre for Molecular Medicine, Lund University Sweden

**Keywords:** glioblastoma, immune cell, pericyte, signaling, smooth muscle cells, transcriptome

## Abstract

Glioblastoma (GBM), the most aggressive brain tumor in adults, is characterized by its infiltrative growth along the perivascular space. Mural cells (MCs), encompassing pericytes and smooth muscle cells, are multifunctional perivascular cells implicated in GBM progression. MCs not only facilitate vascular co‐option but have also been suggested to contribute to the immunosuppressive tumor microenvironment, promoting tumor growth and migration. However, whether MC interactions with immune cells differ based on their proximity to the tumor remains unclear. Using single‐cell RNA sequencing, we analyzed MC transcriptome profiles across distinct regions relative to the tumor mass in mouse and human GBM samples. Tumor‐residing MCs exhibited profound phenotypic changes, showing upregulated gene expression and enhanced signaling activity toward immune cells, with region‐specific ligand–receptor interactions. Conversely, border‐residing MCs, despite their abundance, showed reduced activation and lacked distinct transcriptional profiles. These findings reveal spatially defined transcriptional heterogeneity in MCs within the GBM microenvironment, underscoring their dynamic role in the GBM microenvironment. This study provides novel insights into MC responses in GBM, identifying potential avenues for targeting MC–immune‐cell interactions in therapeutic interventions.

## Graphical abstract

Pericytes alter their transcriptome depending on their proximity to the tumor core. In the tumor core, pericytes display a more active state with higher communication strength but with lower immune activation potential and a shift toward extracellular matrix production. In the border zone, pericytes have downregulated their gene expression consistent with a transition or resting state.

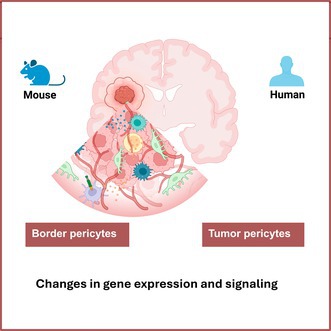

**Abbreviations**
cSMC: classical smooth muscle cells
DC: dendritic cells
DEGs: differentially expressed genes
ecm‐PC: extracellular matrix PC
FGSEA: Fast gene set enrichment analysis
GBM: glioblastoma
GO:BP: Gene Ontology Biological Processes
hiPC: human immune PC
htPC: human transport PC
miPC: mouse immune PC
msPC: mouse signaling PC
mtPC: mouse transport pericytes
PC: pericytes
RGS5: regulator of G protein signaling 5
rSMC: reactive smooth muscle cells
scRNA‐seq: single‐cell RNA sequencing
SMC: smooth muscle cells
TAM: tumor‐associated macrophages
TME: tumor microenvironment

## 1 Introduction

Glioblastoma (GBM) is a grade IV glioma and the most common and aggressive primary brain tumor in adults [1]. Despite advancements in treatment, which typically involve surgical resection followed by chemotherapy, radiotherapy, and temozolomide, the median overall survival remains approximately 16 months, with the latest therapeutic approaches extending it to just 20.9 months [2, 3, 4, 5, 6]. The primary challenges in GBM treatment include its infiltrative growth, making complete resection impossible, and the highly immunosuppressive tumor microenvironment (TME), where immunotherapies have so far shown limited success [7]. The tumor's invasive nature is linked to processes such as vascular co‐option, microvascular proliferation, and abnormal vessel formation [8]. These mechanisms enable tumor cells to exploit the perivascular space for migration, facilitating infiltration into surrounding brain regions. Consequently, residual tumor cells evade surgical resection and drive recurrence, underscoring the urgent need for therapies targeting these invasive pathways [9].

While the role of endothelial cells in the vasculature of GBM is well studied, the role of pericytes—specialized cells that enwrap the brain's microvasculature—remains less well understood in this context [10]. In the TME, pericytes exhibit various abnormalities, including vascular detachment and altered expression of key markers [11, 12]. Notably, pericytes have emerged as key players in the process of vascular co‐option [13].

Under physiological conditions, pericytes regulate both innate and adaptive immune responses [14, 15], either by secreting pro‐ or anti‐inflammatory factors and adhesion molecules that influence immune cells chemotaxis and activation [16, 17, 18, 19, 20, 21], or through their ability to phagocytose and clear toxic cellular debris [13].

In GBM, the interaction between tumor cells and pericytes switches the pericyte function from tumor‐suppressor to tumor‐promoter [13, 22, 23, 24], contributing to the immunosuppressive TME [25]. Using an orthotopic mouse model of GBM [26, 27], we have previously observed pericyte activation not only within the tumor core but also in regions outside the tumor mass. Whether pericytes in the tumor and surrounding regions interact differently with the immune system to either suppress or promote tumor growth and invasion remains unclear.

Here, we examined differences in the transcriptomic signatures of pericytes based on their proximity to the tumor mass, investigating how their functions and their signaling toward immune cells shift depending on the location. Indeed, the glioma border, also defined as peritumoral brain zone (PBZ), represents a critical interface between the tumor and the surrounding brain tissue, making it a key area of investigation in glioblastoma research. This region of a few centimeters surrounding the tumor contains specific molecular, radiological, and cellular alterations that are crucial to understand for effective GBM intervention [28, 29].

In our study, we performed single‐cell RNA sequencing (scRNA‐seq) to profile pericytes in distinct regions of interest in an orthotopic glioma mouse model and analyzed an online dataset of human tissue samples [30]. Our analysis revealed a strong upregulation of gene expression in tumor‐residing pericytes in both mouse and human samples. 228 differentially expressed genes (DEGs) were shared between mouse and human tumor‐residing mural cells and were involved in Gene Ontology Biological Processes pathways associated with RNA processing, suppression of catabolic processes, and immune modulation. Additionally, in both species, we observed a marked increase in communication strength between mural and immune cells within the tumor and region‐specific interactions. Interestingly, pericytes in the border region, despite being the primary origin of mural cells, exhibited lower activation levels with an almost complete absence of upregulated gene expression compared to other regions.

In summary, our findings highlighted that pericytes undergo phenotypic and transcriptional changes depending on their spatial location relative to the tumor. These changes were accompanied by altered signaling strength and changing interactions with immune cells, reflecting the diverse roles that pericytes may play in the GBM microenvironment.

## 2 Materials and methods

### 2.1 Cell culture

The commercially available GL261 mouse glioma cell line (RRID:CVCL_Y003), syngeneic to the C57BL/6 mouse strain, was purchased from DSMZ (Germany, #ACC802) and cultured in Dulbecco's modified Eagle medium (DMEM) + Glutamax medium (Gibco, #61965026) supplemented with 10% fetal bovine serum (FBS) and 2% penicillin/streptomycin. All cultures were maintained at 37 °C in a 5% CO₂ atmosphere. Species‐level identification of GL261 cells was carried out by the producer by mitochondrial Cytochrome C Oxidase Subunit 1 DNA barcoding according to standard ANSI/ATCC ASN‐0003‐2015 and revealed Mus musculus species. Results obtained through sequencing confirmed authentication. All experiments were performed with mycoplasma‐free cells.

### 2.2 GL261 transduction with mCherry

GL261 cells were transduced with mCherry to facilitate visualization when selecting the brain regions for dissection. The pLV‐mCherry lentivirus (Addgene #36084, Watertown, MA, USA) was generated using pMD2.G (#12259), pBR8.91 (#187441), and pLV‐mCherry through Lipofectamine 2000 transfection of HEK293T cells (RRID:CVCL_0063; CRL‐3216 from ATCC, https://www.atcc.org/products/crl‐3216). HEK293T cells were grown in DMEM high glucose, + sodium pyruvate (110 mg·L^−1^) with 10% FBS and (100 μg·mL^−1^) P/S and transfected at ~80% confluency in 6‐well plates. HEK293T cells do not undergo regular authentication verifications, as they are used only for producing the lentiviruses. Virus‐containing supernatants were collected 24 h post‐transfection. GL261 cells were then transduced with 1.5 mL virus‐containing supernatant. Live (DAPI^−^), mCherry‐positive transduced cells were sorted using fluorescent‐activated cell sorting (FACS) Aria II in the Pecy5.5 channel 7 days after transduction. Sorted mCherry^+^ GL261 cells were confirmed to have detectable mCherry expression and were cryopreserved at −150 °C. All experiments were performed with mycoplasma‐free cells.

### 2.3 GL261 tumor cell inoculation *in vivo*

Five *RGS5* ^*+/GFP*^‐C57bl/6 16‐week‐old male mice were used. The RGS5 mouse strain [27] was crossed with C57BL6J (Janvier from Jax), and heterozygote mice were used in this study. In this mouse model, one allele of RGS5 is replaced by green fluorescent protein (GFP), allowing tracking of brain pericytes through GFP expression driven by the RGS5 promoter [27]. RGS5 in the brain is exclusively expressed by mural cells and, therefore, a valuable tool for their identification [31, 32]. Mice were housed under standard conditions with a 12‐h light/dark cycle and access to food and water *ad libitum*. Animals were kept under pathogen‐free conditions in individually ventilated cages. All animal procedures were approved by the Committee of Animal Ethics in Lund‐Malmö, Sweden (Ethical Permit 14 074/2020). All experiments were conducted in accordance with the Swedish Board of Agriculture and reported according to the ARRIVE guidelines. Mice were anesthetized with isoflurane and secured in a stereotaxic frame, followed by subcutaneous injection of 0.025 mL Marcaine (AstraZeneca). GL261 tumor cells (100 000 cells in 2 co‐express DMEM) were injected over 3 min via a burr hole into the caudate nucleus using a 10 μL Hamilton syringe (Hamilton Bonaduz AG, Switzerland). The injection site was located 1 mm anterior to the bregma and 1 mm to the right of the midline, along the three‐dimensional axis, corresponding to the frontal and lateral parts of the striatum. The needle remained in place for 5 min before being slowly retracted. Mice were perfused on day 21 post‐tumor inoculation.

### 2.4 Cell processing for scRNA‐seq

Bulk live non‐neuronal cells were isolated and processed according to previously published protocols [33, 34]. Briefly, mice were transcardially perfused with saline (0.9% NaCl in dH₂O). Following the removal of the brain, the tumor core and the border were selected based on fluorescence of mCherry tumor cells under UV light. The tumor core was defined as the region where mCherry^+^ tumor cells were macroscopically visible, while the tumor border was dissected as the surrounding brain tissue, where no mCherry fluorescence was detected by eye under UV light. This definition was further validated by FACS analysis and sequencing data. The contralateral caudate nucleus was also dissected and dissociated.

Cells were filtered, pelleted, and resuspended in ice‐cold 20% bovine serum albumin (BSA) for myelin removal and then resuspended in 0.5% BSA in PBS and kept on ice. FACS sorting was used to exclude dead (DAPI^+^) cells and doublets. As an additional verification step before sequencing, flow cytometry was performed on separate aliquots from the samples designated for sequencing to confirm the presence of GL261 (mCherry^+^), immune cells (CD45^+^), and pericytes (PDGFRβ^+^, CD13^+^, GFP^+^, PECAM1^−^). After sorting, live, singlet cells from the sequencing aliquots were spun down and counted with Trypan blue to assess cell viability. 8500 live cells were prepared in 45 μL PBS, 0.5% BSA for 10× Chromium v.3.13′ droplet labeling and scRNA‐seq on an Illumina sequencer.

### 2.5 Bioinformatic analysis on mouse samples

After running Cellranger (v.5.0.1) on the fastq files from the sequencing, we eliminated technical artifacts using Cellbender (v.0.3.0) on each sample separately. Data were then processed in R 4.3.1 and Seurat v.5.0.2 [35]. Doublets were filtered out with scDblFinder [36], and the singlets were subsequently quality filtered with the following parameters: nCount_RNA > 300, nFeature_RNA > 200, mitochondrial genes <10%. Summary of the output numbers from Cellranger for the mouse dataset can be found in Table S1A.

After initial evaluation, since the samples originated from the same batch of 10X analysis and we wanted to distinguish clusters specific to the single regions, samples were not integrated. To define cellular clusters, we used the Louvain algorithm with resolution set to 0.8 in order to distinguish among the expected clusters of interest, that is, at least one cluster of pericytes. To define cluster identities, we compared the expression of cell type‐specific transcripts to the transcripts reported in the literature [37, 38, 39, 40, 41, 42, 43, 44], and on PanglaoDB [45]. To further divide the pericytes, we used the Seurat function Subset. The object was then further processed with DietSeurat, NormalizeData, FindVariableFeatures, ScaleData, RunPCA, FindNeighbors, FindClusters, and RunUMAP. The Louvain algorithm resolution was set to 0.5 to define the mural cells subclusters. To compare the distribution of the clusters in each region of interest, the number of cells from each cluster was normalized for the total number of cells of the sample of origin. Two‐way ANOVA followed by Tukey's *post hoc* comparison was performed on the data to evaluate significant changes in the cell percentage of each cluster among the areas. The two clusters of tumor cells were excluded from the statistical analysis of distribution because they were present only in the tumor core. Differentially expressed genes (DEGs) among mural cells were calculated with the FindAllMarkers function and filtered for an adjusted *P*‐value <0.05. Fast gene set enrichment analysis (FGSEA) was performed on the DEGs using the Msigdbr package in R to define Gene Ontology Biological Processes (GO:BP) gene set enrichments within the data. Interacting receptor/receptor‐secreted molecule pairs were studied with CellChat [46]. CellChat is a computational tool designed to infer, analyze, and visualize intercellular communication networks from scRNA‐seq data. By using known ligand–receptor interactions, CellChat predicts signaling pathways that could mediate cell–cell communication, helping to identify key cellular interactions within the dataset.

### 2.6 Bioinformatic analysis on published human dataset

Raw scRNA‐seq count data were downloaded from the NCBI Gene Expression Omnibus under accession number GSE162631 and processed in R 4.3.1 and Seurat v. 5.0.2 according to the original publication [30]. A summary of the cell numbers for the human dataset can be found in Table S1B. An additional step of doublet elimination was performed by using scDblFinder [36]. The same procedure as the mouse data was followed to compare the distribution of the clusters in each region of interest. Mural cell clusters were isolated for further analysis with the subset function in Seurat and reclustered using the Louvain algorithm (resolution = 0.5). Interacting ligand/receptor molecule pairs were studied with CellChat [46]. Due to significant differences in cellular composition between malignant and non‐malignant tissues in the published human dataset, direct comparison of mural cell crosstalk across regions, as performed in the mouse dataset, was not feasible. Many immune cell clusters were exclusive to either malignant or non‐malignant regions, preventing direct regional comparisons. Consequently, analyses were conducted separately for tumor and non‐malignant tissues.

### 2.7 Mouse–human shared DEGs and pathways analysis

Tumor‐enriched DEGs were first obtained separately in the two datasets comparing tumor to contralateral‐residing mural cells (for mouse) and tumor to non‐malignant‐residing mural cells (for human). DEGs were calculated using the same parameters in both datasets (minimum number of cells = 3, maximum number of cells = 80 (mouse) or 100 (human), random.seed = 3, log2FC threshold = 0.15, test.use = “wilcox”, min.pct = 0.1, min.diff.pct = 0.1, only.pos = TRUE). FGSEA analysis on the shared DEGs was performed using the Msigdbr package in R. The analysis was run separately for mouse and human using the corresponding species gene sets.

### 2.8 Immunohistochemistry

On day 21 post‐tumor inoculation, two mice were perfused with paraformaldehyde (PFA) solutions. The brains were removed, post‐fixed in 4% PFA at 4 °C overnight and then transferred to a 30% sucrose solution. Brains were sectioned into 40 μm‐thick coronal sections using a Leica SM200 R sliding microtome and stored at +4 °C in antifreeze solution. Sections were washed in PBS and blocked with 10% normal donkey serum (NDS) and 0.75% Triton X‐100 in PBS for 1 h at room temperature (RT). Following this, sections were incubated overnight at 4 °C with primary antibodies diluted in blocking solution. The next day, sections were washed and incubated for 1 h at RT with secondary antibodies diluted in PBS. After secondary antibody incubation, sections were briefly incubated with DAPI for 5 min. Immunofluorescence images were acquired using a Leica DMi8 confocal microscope with a 40x objective. Z‐stacks were acquired with a depth of 40 μm. Images were analyzed and assembled using fiji (imagej) software (versions 2.16.0/1.54p) [161]. 2D images were generated from z‐stacks using the “Max Intensity” projection, followed by “Subtract Background” (rolling ball radius: 50 pixels) and “Despeckle” functions. Color intensity was adjusted consistently across all images to ensure comparability.

## 3 Results

### 3.1 Cellular clusters distribution varies depending on glioblastoma proximity in mouse GBM

**Fig. 1:**
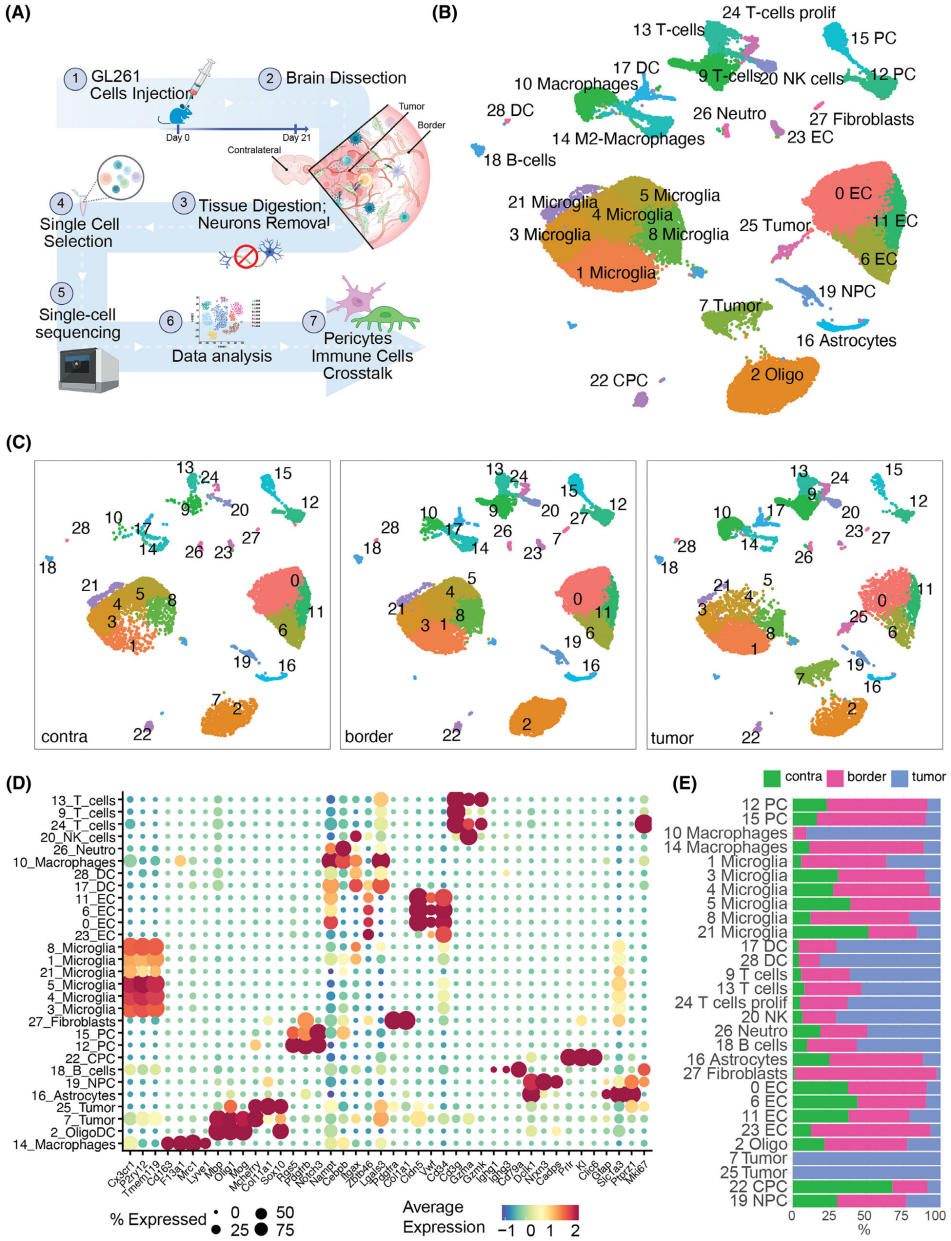
Overview of experimental design and characterization of cell populations in the different regions. (A) Schematic representation of the experimental setup. (B) UMAP visualization of cells, categorized into distinct clusters and (C) divided by the three regions: contralateral (contra), border, and tumor core. The UMAPs are colored by cluster. (D) Dot plot depicting the marker genes defining each cell cluster. (E) Bar plot illustrating the relative distribution of each cell type in each region. Green = contralateral; magenta = border; blue = tumor. Bar lengths represent the average percentage per cluster. UMAP, Uniform Manifold Approximation and Projection; CPC, choroid plexus cells; DC, dendritic cells; EC, endothelial cells; OligoDC, oligodendrocytes; NK, natural killer; NPC, neuron precursor cells; PC, pericytes. Number of animals per region = 3.

Following selection of regions of interest (Fig. 1A), we profiled 49 292 individual cells, each with an average median expression of 1600 genes (Table S1A). Cell clusters were annotated based on established cell type‐specific markers (Fig. 1B–D, Table S2). We identified two distinct mural cell populations characterized by the expression of *Rgs5‐GFP*, *Rgs5*, Platelet‐derived growth factor receptor beta (*Pdgfrβ*), and Notch receptor 3 (*Notch3*) (Fig. 1D). To confirm pericyte identity, we cross‐referenced these clusters with the marker profile proposed by Oudenaarden *et al*. [38] (Fig. S1B). Spatial analysis revealed that the majority of pericytes and SMC localized at the tumor border, comprising 1617 cells (7.1% of the total cell population in this region). This was followed by the contralateral region (460 cells, 4.1%) and the tumor core (215 cells, 1.48%) (Fig. 1E, Table S1A, Fig. S1A,C,D).

It is well established that GBM exhibits robust infiltration of tumor‐associated microglia and macrophages (TAMs) [47], which are also often referred to as “GAMMs” (glioblastoma‐associated microglia and macrophages) [48, 49, 50]. Here, we identified several TAMs clusters (Fig. 1D, Fig. S2). Among these, six clusters expressed typical microglial markers (*Cx3cr1*, *Tmem119*, *P2ry12*), and two exhibited macrophage markers (*Cd163*, *F13a1*). Supporting this microglia–macrophage distinction, the macrophages clusters corresponded to the monocytes/TAM1 categories defined by Pombo‐Antunes *et al*. [51] (Fig. S3A), representing cells with a monocyte ontogeny. Macrophage cluster 14 also expressed perivascular macrophage markers (*Cd163*, *Mrc1*, *Lyve1*) [52] (Fig. 1D). Microglial clusters were classified as TAM2 (Fig. S3A) [51], reinforcing their microglial origin. Microglia cluster 21, on the other hand, did not correspond to any of the two categories (Fig. S3A), suggesting a distinct origin. In general, each of the TAM clusters displayed specific regional origin enriched genes and pathways (Fig. S1A; Fig. S2). Additionally, we identified two dendritic cells (DCs) clusters (*Itgax*, *Zbtb46*), with cluster 17 corresponding to classical DC signatures from [51], while cluster 28 corresponded to a pre‐DC signature [51] (Fig. S3B). We then identified three T cells clusters (*Cd3g*, *Gzma*, *Gzmk*). Clusters 9 and 24 expressed a T‐regulatory signature [51], whereas cluster 13 had a cytotoxic T‐cell signature, expressing *Cd8a*, *Cd8b1*, and *Gzmb* (Fig. 1D, Figs S3C, S4).

### 3.2 Mural cells subclusters redistribute depending on the proximity to the GL261 tumor core

**Fig. 2:**
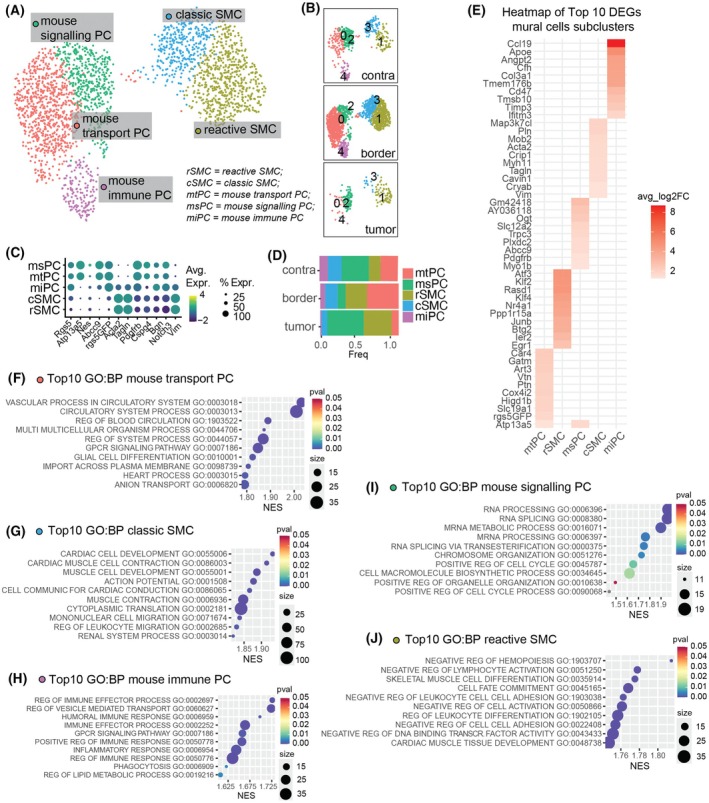
Subclustering of mural cells and differential gene expression analysis by subcluster division. (A) UMAP visualization of mural cells, colored according to subclusters and (B) split into the three regions: contralateral (contra), border, and tumor core. (C) Dot plot showing the expression of pericyte and smooth muscle cell markers. (D) Bar plot displaying the relative distribution of each mural cell subclusters within each region. Colors indicate the subclusters. (E) Heatmap showing the 10 top differentially expressed genes for each mural cell subcluster, calculated by comparing each subcluster against all other mural cell subclusters, colored according to the average fold change increase. (F–J) Dot plots showing the significantly enriched Gene Ontology Biological Processes for mtPC (F), cSMC (G), miPC (H), msPC (I), rSMC (J). avg log_2_FC, average log_2_ fold change; cSMC, classical smooth muscle cells; DEGs, differentially expressed genes; GO:BP, Gene Ontology Biological Processes; mtPC, mouse transport pericytes; msPC, mouse signaling pericytes; miPC, mouse immune pericytes; NES, normalized enrichment score; rSMC, reactive smooth muscle cells; UMAP, Uniform Manifold Approximation and Projection. Number of animals per region = 3.

To further identify regional differences in transcriptomic profiles of the mural cell population, we performed subclustering analysis, identifying five subclusters. Based on marker expression, we resolved two pericyte subclusters, two SMC subclusters, and one subcluster with an intermediate phenotype (Fig. 2A–C). One pericyte subcluster exhibited a classic phenotype enriched in vascular and transport function pathways, while the other showed an increase in RNA splicing and cell cycle pathways, suggesting a more active phenotype (Fig. 2E,F,I). These were respectively designated as “mouse transport” pericytes (mtPC) and “mouse signaling” pericytes (msPC). Similarly, the two SMC subclusters included one with a classical phenotype and another reactive type, associated with hematopoiesis, lymphocyte activation, and cell adhesion pathways (Fig. 2E,G,J). We named these “classical” SMC (cSMC) and “reactive” SMC (rSMC), respectively. One subcluster expressed markers of both pericytes and SMCs, indicating an intermediate phenotype. This subcluster was enriched in immune response and phagocytosis pathways (Fig. 2E,H) and was therefore named “mouse immune” PC (miPC).

All subclusters were present in the contralateral region, with msPC being the most common, followed by mtPC, cSMC, rSMC, and miPC (Fig. 2D). In the tumor core, rSMC and msPC predominated, while at the tumor border, mtPC was the most abundant subcluster. Overall, these findings suggest that pericyte subclusters undergo region‐specific redistribution within the TME.

To validate our single‐cell findings, we performed immunohistochemistry on selected genes among the Top 10 marker genes from the immune PC cluster. We observed that CCL19, APOE, and ANGPT2 were predominantly expressed in cells co‐expressing CD13 and ASMA, consistent with the immune PC phenotype identified in our transcriptomic analysis (Fig. S5).

### 3.3 Human mural cell subclusters redistribute depending on their region of origin

We then investigated mural cells in human GBM using a previously published dataset, where tissue samples were obtained from either the tumor core or neighboring non‐malignant brain tissue [30]. After initial clustering (Fig. S6A–E), we focused on pericytes and immune cells. Pericytes were identified by *RGS5*, *PDGFRβ*, and *NOTCH3* expression, in addition to the pericyte marker profile proposed before [38] (Fig. S6F, Table S3). In total, after the processing steps, we obtained 264 (0.6%) mural cells in the non‐malignant region and 708 (1.3%) from the tumor (Table S1B). We then identified seven clusters: CD163^+^, F13A1^+^ which we defined as macrophages; six clusters: CX3CR1^+^, P2RY12,^+^ and TMEM119^+^ which we identified as microglia; one cluster: MBP^+^, NAMPT^+^, CEBPB^+^, ITGAX^+^ as neutrophils; one cluster of cytotoxic T cells (CD3G^+^, CD8A^+^, GZMA^+^, GZMB^+^, GZMK^+^) and one cluster of B cells (IGHG1^+^, CD79A^+^). Following the categories defined by Pombo‐Antunes *et al*. [51], four out of seven among our macrophage clusters corresponded to the TAM1 signature [51], one to the proliferating‐TAM signature [51], and one to the monocyte; all our microglial clusters corresponded to the TAM2 signature (Fig. S3D) [51].

**Fig. 3:**
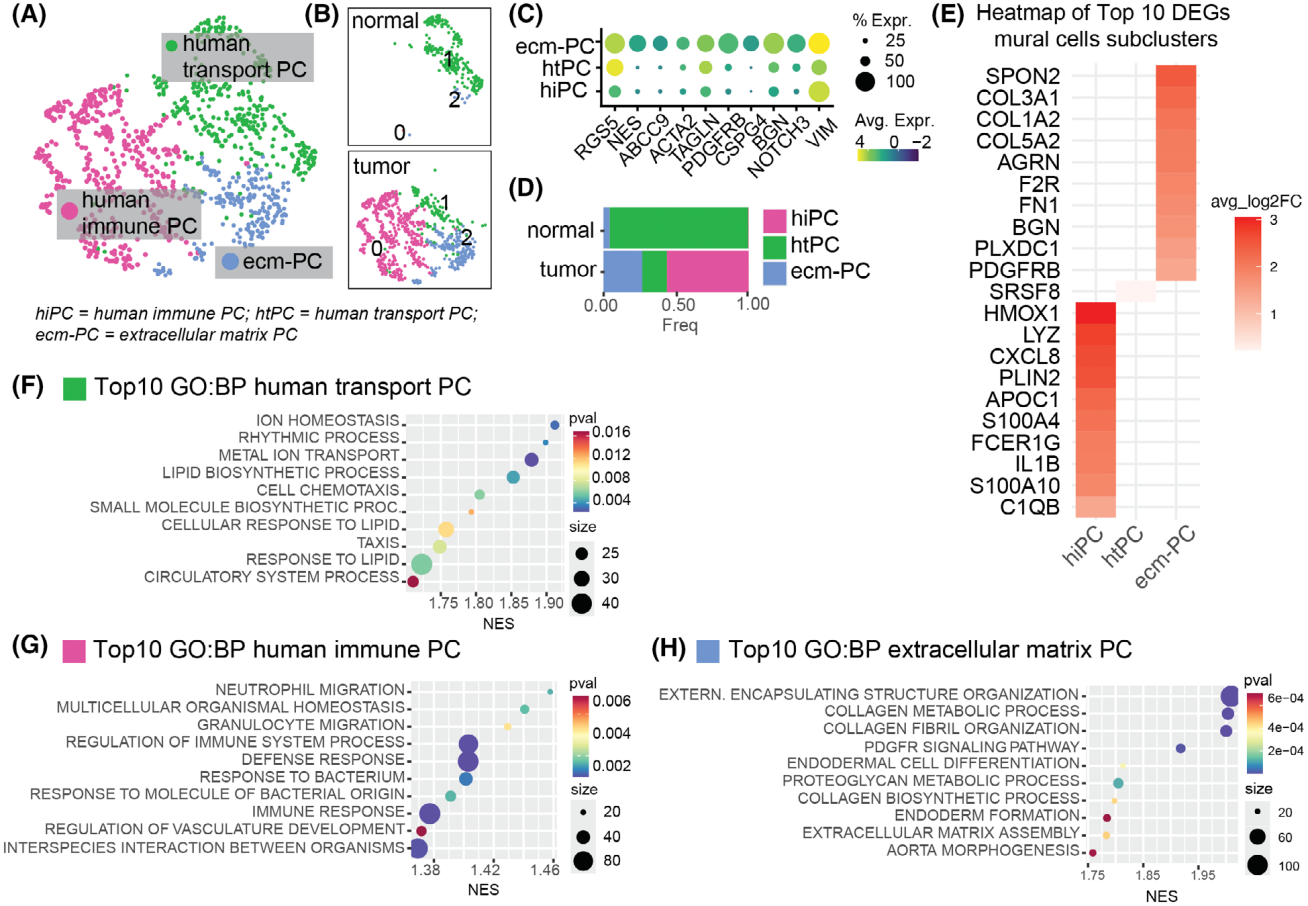
Subclustering and differential gene expression analysis of human mural cells from the Xie et al. dataset [30]. (A) UMAP visualization of mural cells, colored by distinct subclusters and (B) divided by region. (C) Dot plot showing the expression of pericyte and smooth muscle cell markers across subclusters. (D) Relative distribution of mural cell subclusters in each region, presented as a bar plot. Colors indicate the subclusters. (E) Heatmap of the 10 top DEGs for each mural cell subcluster colored by average fold change. (F–H) Dot plots of significantly enriched GO:BP for htPC (G), hiPC (H), and ecm‐PC. avg log2FC, average log2 fold change; DEGs, differentially expressed genes; GO:BP, Gene Ontology Biological Processes; hiPC, human immune pericytes; htPC, human transport pericytes; ecm‐PC, extracellular matrix pericytes; NES, normalized enrichment score; UMAP, Uniform Manifold Approximation and Projection. Number of patients per region = 4.

Subclustering analysis in the human GBM dataset identified three distinct subclusters of pericytes (Fig. 3A–D). Signature markers did not clearly differentiate pericytes from SMC, suggesting that the two mural cell types were intermixed in this dataset. Subcluster 0, which constituted the majority of the mural cells within the tumor, originated exclusively from the tumor core. This subcluster was characterized by upregulated genes related to immune functions such as cytokines, complement system, and MHC‐II complex, as well as pathways involved in immune responses (Fig. 3B–E,G; Fig. S6G,H). We designated these cells as “human immune” pericytes (hiPC). Subcluster 1, representing nearly all mural cells in normal tissue, was enriched in pathways related to ion homeostasis, lipid biosynthesis and chemotaxis (Fig. 3E,F). Given its association with normal tissue, we defined these cells as “human transport” pericytes (htPC). Subcluster 2 was mainly found in the tumor while also being present in the non‐malignant tissue. This subcluster 2 was especially enriched for extracellular matrix (ECM) components (Fig. 3E,H) with pathway enrichment in collagen metabolism, PDGFR signaling, and external structure and ECM organization. We therefore designated these cells as “extracellular matrix pericytes” (ecm‐PC).

In summary, similarly to the rodent data, human mural cell subclusters showed region‐specific redistribution, with non‐malignant tissue predominantly characterized by a single phenotype, while tumor tissue displayed a variety of phenotypes. Human mural cells tended to cluster more by function than by phenotype, in contrast to mouse mural cells, which showed a clearer distinction between pericytes and SMCs. The mouse and human subclusters of pericytes identified were different in gene expression and function, suggesting that subclusters do not fully overlap across species, in line with observations from previous reports [53, 54, 55], with the exception of the pericytes with an immune signature found in both species.

### 3.4 Mouse and human tumor‐residing mural cells shift their gene expression depending on the tumor proximity

We then evaluated if mural cells changed their overall transcriptional profile depending on the region of origin (Fig. S7).

Classical pericyte and SMC markers were generally comparable across the regions in the mouse model, except for an elevation of Nestin in the tumor (Fig. S7A,B).

**Fig. 4:**
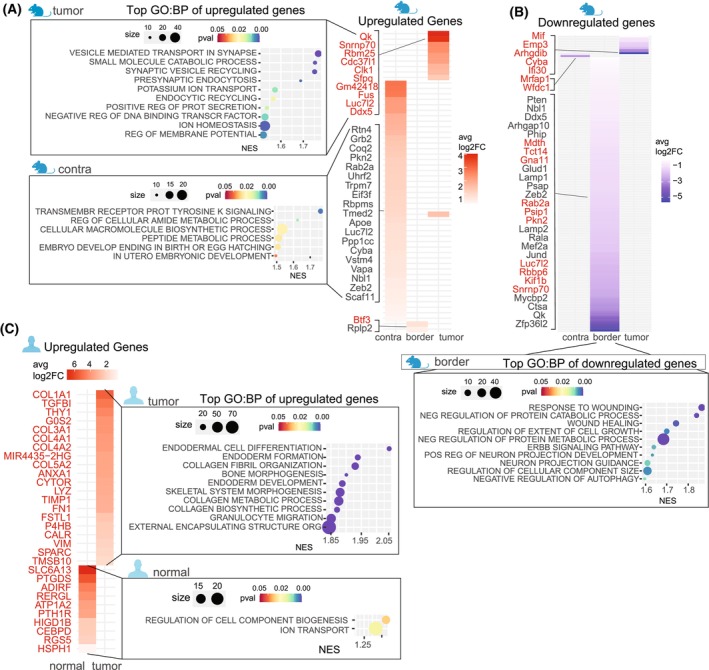
Differential gene expression and pathway analysis of mural cells across regions in mouse and human. (A, B) Heatmaps displaying the top upregulated (A) and downregulated (B) DEGs in mural cells, categorized by region in the mouse. Genes highlighted in red have a *P*‐value <0.05. GO terms associated with DEGs from each regional mural cell population are indicated in boxed sections. (C) Heatmap of the top upregulated DEGs for human mural cells, categorized by region. GO terms for each area's DEGs are noted in boxed sections. avg log_2_FC, average log_2_ fold change; DEGs, differentially expressed genes; GO:BP, Gene Ontology Biological Processes; NES, normalized enrichment score. Number of animals per region = 3; number of patients per region = 4.

We then examined further transcriptional changes that might be associated with functional alterations. In the mouse, the contralateral region was characterized by transmembrane receptor signaling, metabolic processes, and upregulated genes related to embryonic development, consistent with normal pericyte functions (Fig. 4A). At the border, we observed a general downregulation of genes (Fig. 4A,B). The downregulated genes were related to wound responses, negative regulation of catabolism, autophagy, metabolism, cellular growth, and others (Fig. 4B). In other words, in the border, pericytes shifted toward functions indicating an increased catabolism and autophagy. This may reflect a change in energy utilization or an adaptation to external stressors, whereas reparative and supportive functions such as wound healing and neuronal support seem to be less prioritized by those cells. In the tumor core, we observed a strong upregulation of gene expression. Associated pathways included vesicle transport, catabolism, endocytosis, ion homeostasis, protein secretion, and transcription factor regulation (Fig. 4A). Upregulation of these pathways likely highlights the adaptation of the pericytes to the challenging tumor environment, enabling them not only to survive but also to actively influence and remodel the surrounding microenvironment.

In the human dataset, DEG analysis between tumor‐derived and non‐malignant mural cells showed increased expression of collagen isoforms, fibronectin, *G0S2*, and immune‐related genes in tumor‐resident mural cells (Fig. 4C), which led to increased pathways such as endodermal cell differentiation, collagen synthesis and metabolism, bone and skeletal system morphogenesis, and granulocyte migration (Fig. 4C). Top DEGs in non‐malignant tissue included transporters *SLC6A13*, *ATP1A2*, and *RGS5*, with pathways associated with cellular component biogenesis and ion transport (Fig. 4C). Notably, *ATP1A2* is well‐established marker for pericytes, while *SLC6A13* is one of the characteristic genes of classical transport pericytes [54, 55, 56].

Overall, tumor‐resident pericytes in human GBM shifted toward an immune and matrix‐associated phenotype, whereas pericytes in normal tissue retained homeostatic and transport functions. Mural cells in the mouse tumor border showed substantial downregulation of gene expression compared to other regions, potentially indicating a transition stage. This highlights distinct regional functional adaptations within the tumor microenvironment.

### 3.5 Tumor‐residing mural cells shared 228 upregulated genes across species

**Fig. 5:**
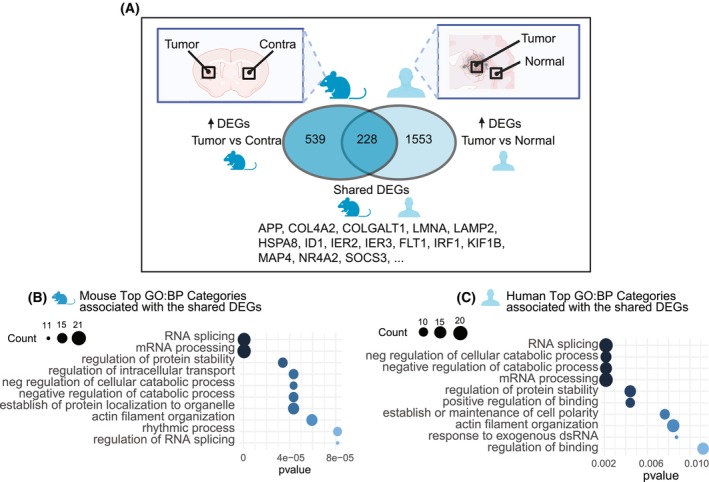
Shared DEGs and pathways between species. (A) Schematic of shared genes selection process: DEGs in mouse tumor mural cells were compared to contralateral cells and cross‐referenced with DEGs in human tumor mural cells versus non‐malignant tissue. Venn diagram illustrates the 228 shared DEGs, listed in Table S4C. (B, C) Dot plots showing significantly enriched GO processes for the 228 shared genes, with panel B using mouse *P*‐values and fold changes and the mouse GO database, and panel C using human *P*‐values and fold changes and the human GO database. DEGs, differentially expressed genes; GO:BP, Gene Ontology Biological Processes; neg, negative. Number of animals per region = 3; number of patients per region = 4.

To identify conserved transcriptional responses, we compared DEGs between tumor and non‐tumor mural cells across species (Fig. 5A). From 767 mouse DEGs and 1781 human DEGs, we identified 228 shared upregulated genes (Table S4A–C). FGSEA analysis revealed associations with RNA splicing, actin filament organization, and suppression of catabolic processes, suggesting conserved mechanisms by which mural cells contribute to the TME (Fig. 5B,C). RNA splicing is essential for mRNA maturation and hence protein synthesis, and it also generates diverse protein isoforms that may facilitate mural cell adaptation to the TME. The suppression of catabolism could imply a shift away from energy generation via macromolecule breakdown, potentially favoring anabolic processes that support biosynthesis of proteins, lipids, and nucleic acids. Actin filament organization, critical for cytoskeletal dynamics, influences cell migration and adhesion and plays a role in autophagy, which may help cells manage stress and damaged components under suppressed catabolic activity. In summary, while the majority of tumor DEGs were species‐specific, we identified a significant overlap in the upregulated genes and their associated functions, pointing to conserved mechanisms through which mural cells respond to the GBM microenvironment.

### 3.6 Communication analysis revealed region‐specific ligand–receptor interactions between mural and immune cells in murine GBM

Previous studies suggested that pericytes switch from tumor‐suppressors to tumor‐promoters, contributing to the immunosuppression in GBM [13, 22, 23, 24]. To elucidate whether mural cells in the tumor, border, and contralateral regions engaged in different communication patterns with immune cells, we utilized CellChat to model the ligand–receptor (L‐R) interactions.

**Fig. 6:**
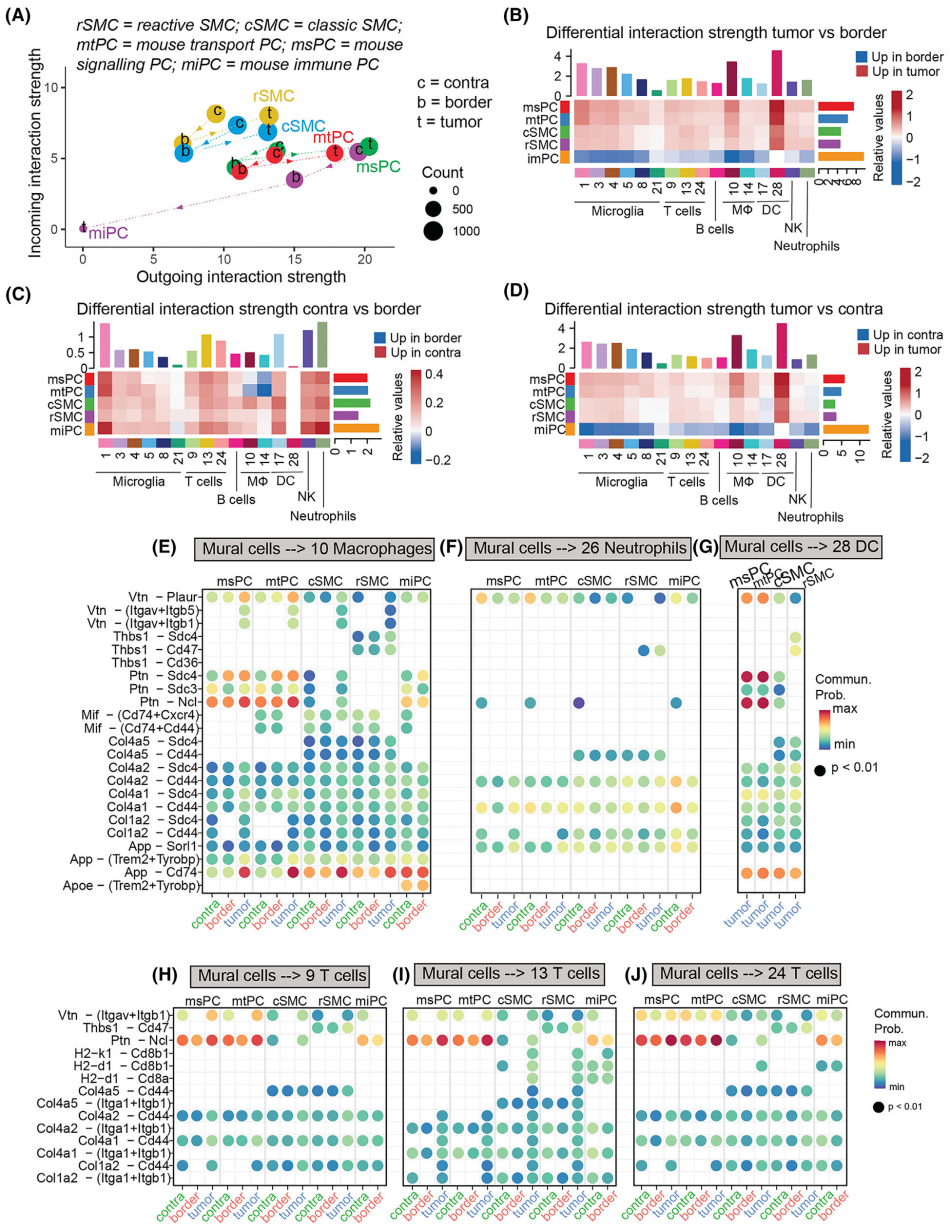
Cell–cell communication analysis of mouse mural cells using CellChat. (A) Scatterplot illustrating the total outgoing and incoming interaction strength for each mural cell subcluster across the contralateral (c), border (b), and tumor (t) regions in the mouse dataset. The x‐axis represents the total outgoing communication probability, while the y‐axis represents the total incoming communication probability for each cell group. Dot sizes correspond to the number of inferred connections outgoing and incoming), and colors denote mural cell subclusters. Arrows on the dashed lines indicate the changes in communication as mural cells transition from the contralateral region to the border and into the tumor. (B–D) Heatmaps comparing differential interaction strength among cell populations across the three regions. The top‐colored bar plot above each heatmap shows the sum of absolute values in each column, representing the total incoming signaling strength, while the right‐colored bar plot shows the sum of absolute values in each row, representing the total outgoing signaling strength. Bar heights indicate the magnitude of changes in the number or strength of interactions. In each heatmap, red denotes increased signaling, and blue indicates decreased signaling between regions. Specifically, (B) highlights differences between the tumor and the border, (C) compares the contralateral region to the border, and (D) shows differences between the tumor and the contralateral region. (E–J) Bubble plots showing outgoing signaling from mural cell subclusters to specific immune cell clusters in each region. Rows display selected ligand–receptor pairs, while columns correspond to the contralateral, border, and tumor regions for each mural cell subcluster. Colors in the dots represent the communication probability, and dot sizes indicate the statistical significance *P*‐value. (E) shows interactions with cluster 10 macrophages, (F) with cluster 26 neutrophils, (G) with cluster 28 dendritic cells, (H) with cluster 9 T cells, (I) with cluster 13 T cells, and (J) with cluster 24 proliferating T cells. cSMC, classical smooth muscle cells; DC, dendritic cells; mtPC, mouse transport pericytes; msPC, mouse signaling pericytes; miPC, mouse immune pericytes; Mφ, macrophages; rSMC, reactive smooth muscle cells; NK, natural killer. Number of animals per region = 3.

Overall, outgoing communication from mural cells was the highest in the tumor and the lowest in the border (Fig. 6A–D) indicating an increase in signaling activity, aligned with the general increase in gene expression.

In the mouse tumor core, msPC were the most active senders, while miPC had a minimal role (Fig. S8A). Conversely, miPC dominated the communication in the contralateral and border (Fig. 6A; Fig. S8A). In the tumor core, the strongest communication was predicted toward cluster 10 of macrophages and pre‐DC (Fig. 6B,D). Communication was slightly enhanced toward neutrophils in the contralateral region (Fig. 6C,D). At the tumor border, communication was generally less probable than in the contralateral or tumor regions, with no unique upregulated pathways.

Next, we investigated if mural cells varied their communication strength of specific L‐R pairs between regions. We focused on the most relevant clusters based on the heatmaps previously generated, and within the communication with those clusters, we evaluated the pathways with the highest communication probability.

Several signaling pathways were significantly upregulated in the tumor as part of the communication between pericytes and macrophages (cluster 10). Key interactions included Vitronectin (*Vtn*) with the Urokinase Plasminogen Activator Receptor (encoded by *Plaur*) and integrins, Pleiotrophin (*Ptn*) with Syndecan‐4 (*Sdc‐4*) and Nucleolin (*Ncl*), and Amyloid Precursor Protein (*App*) with *Cd74* (Fig. 6E). Vitronectin‐uPAR interactions promote matrix remodeling, immune responses, and immune cell infiltration [57, 58, 59], while interactions with vtn‐integrins primarily mediate adhesion. Sdc‐4 is implicated in immune cell migration and adhesion [60, 61, 62] and Ncl is implicated in recognizing and phagocytose apoptotic cells [63, 64, 65]. Macrophage cluster 10 might therefore exhibit scavenger‐like activity, heightened in the tumor core. Increased activity of these pathways in the tumor might reflect enhanced pericyte support for pro‐inflammatory macrophage chemotaxis and activation. App‐Cd74 inhibits the phagocytosis of APP‐expressing cells [66], suggesting that it might be a pericyte survival signal. *Ptn‐Sdc3* was enriched in the contralateral region (Fig. 6E). miPC was the only subcluster that communicated via *ApoE‐*(*Trem2 + Tyrobp*) (Fig. 6E). Apoe is associated with lipid delivery to tumor cells and lipid recycling by macrophages in necrotic areas [67], while Trem2 is a known phagocytic immunomodulator in gliomas [68] and promotes microglial survival [69].

Communication between mural cells and DC 28 was similar to macrophages 10 (Fig. 6G), while mural cell communication with neutrophils was generally weaker (Fig. 6F), consistent with the reduced signaling observed in heatmap analyses (Fig. 6B,D). Unlike the macrophage interactions, *Vtn‐Plaur* and *Ptn‐Ncl* with neutrophils were stronger in the contralateral region compared to the tumor (Fig. 6F). Furthermore, miPC showed unique *collagen IV–Cd44* signaling, again more prominent in the contralateral region. This suggests that pericyte‐mediated adhesion to neutrophils is favored in non‐tumor tissue, despite the higher abundance of neutrophils in the tumor.

For T‐cell communication, tumor‐residing SMC and cytotoxic T cells (Fig. 6I) communicated through *MHC‐I–Cd8* signaling. *MHC‐I* signaling partially targeted cluster 24 but was absent toward cluster 9 of T reg. Pericytes did not engage in MHC‐I signaling, suggesting that pericytes and SMCs engage in diverse communication patterns toward T cells. miPCs signaled via *MHC‐I* in both the contralateral and border regions, reinforcing their role in immune regulation even in the contralateral hemisphere (Fig. 6I). In the border and contralateral regions, miPC also communicated with cytotoxic T cells through *MHC‐I* signaling. Collagen I and collagen IV signaling with integrins was also exclusive to the cytotoxic T‐cell cluster (Fig. 6I), highlighting a potential role for mural cells in cytotoxic T‐cell adhesion, whereas collagen*–Cd44* signaling was stronger with the regulatory T‐cell clusters 9 and 24 (Fig. 6H,J). With all the T‐cell clusters, pericyte subclusters communicated strongly through *Vtn‐*integrins and *Ptn‐Ncl* signals, again increased in the tumor (Fig. 6H–J). Overall, the decreased *MHC‐I* signaling to cytotoxic T cells in the tumor may contribute to the immunosuppressive TME.

In conclusion, mouse tumor‐residing mural cells engaged in stronger communication with immune cells. This increased communication was mainly directed toward one cluster of macrophages and one cluster of DC and involved pathways such as *Vtn‐Plaur*, *Vtn‐integrins*, *Ptn‐Ncl*, and collagen*‐Cd44* and collagen*‐Sdc4*, related to adhesion. This further indicates that the normal pericyte function is changed in the tumor core, possibly leading to altered immune cell infiltration dynamics.

### 3.7 Communication analysis of human mural cells predicted increased communication strength in the tumor

**Fig. 7:**
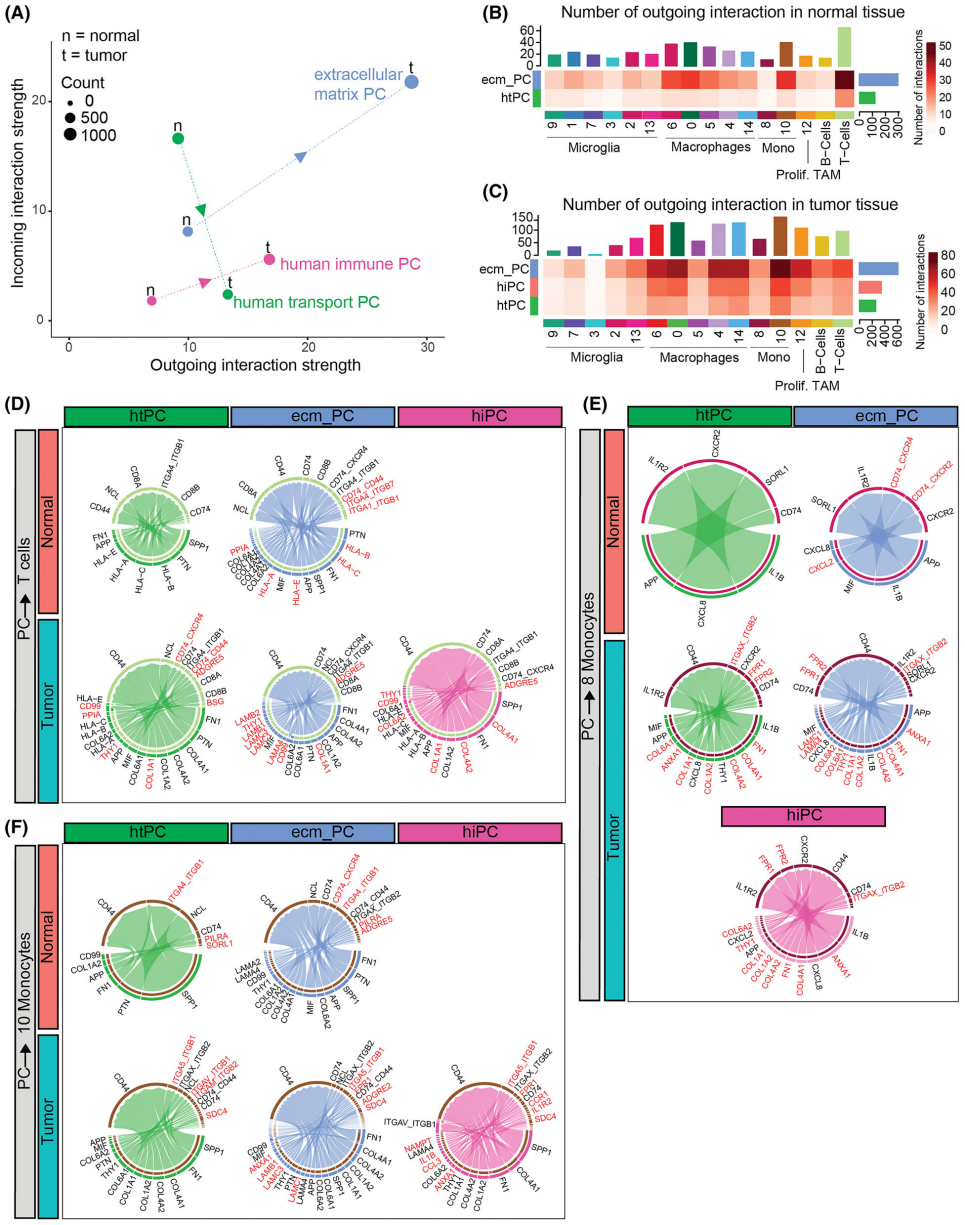
Human cell–cell communication analysis using CellChat. (A) Scatterplots showing the total outgoing or incoming communication probability interaction strength) for each mural cell subcluster in the human dataset across the non‐malignant tissue (n) and tumor (t). Dot sizes represent the number of inferred connections outgoing and incoming, and colors denote mural cell subclusters. Arrows on the dashed lines indicate the changes in communication as mural cells transition from the normal to the tumor tissue. (B, C) Heatmaps displaying differential interaction strength among cell populations across the two regions. The top‐colored bar plot represents the sum of the absolute values in each column, indicating incoming signaling strength. The right‐colored bar plot represents the sum of absolute values in each row, representing outgoing signaling strength. The bar height indicates the degree of change in the number of interactions between the conditions. The intensity of the red color denotes the number of interactions in the (B) non‐malignant tissue and (C) tumor tissue. (D–F) Chord diagrams illustrating the significantly upregulated signaling ligand–receptor pairs from a specific pericyte subcluster toward a specific immune cell cluster either in the non‐malignant or in the tumor region. htPC green, ecm‐PC blue and hiPC red signals toward T cells (D), cluster 8 of monocytes (E) and cluster 10 of monocytes (F) are displayed. Genes that are specific for a region within the considered mural cell cluster are denoted in red. hiPC, human immune PC; htPC, human transport PC; ecm‐PC, extracellular matrix PC; PC, pericytes; TAM, tumor‐associated macrophages. Number of patients per region = 4.

CellChat analysis predicted a significantly higher probability of communication for tumor‐residing pericytes compared to those in non‐malignant tissues (Fig. S8B), a result consistent with the mouse model. Also, similarly to the mouse data, all the pericyte subclusters interacted with every cluster of immune cells in the tumor (Fig. 7A–C). Despite ecm‐PC representing only 5% of the mural cells in the normal tissue, they were predicted to be stronger senders than htPC (Fig. 7A). The strongest signal in the normal tissue was delivered to T cells, while in the tumor, the strongest signals were delivered to cluster 10 of monocytes, followed by clusters 0, 4, 6, and 14 of macrophages (Fig. 7B,C).

Interestingly, the communication between pericytes and T cells changed depending on the location. For example, while normal tissue pericytes and T‐cell signaling included several *MHC‐I to CD8A/CD8B* receptors, those decreased in the tumor while fibronectin and collagen signals increased, suggesting a reduced antigen presentation ability of pericytes in the tumor (Fig. 7D).

Although pericytes expressed MHC‐II, this expression was at low levels, and no communication between pericytes and T cells involving MHC‐II was predicted. However, CellChat predicted MHC‐II‐based communication between ECM‐PC and microglia clusters 1 and 2 within the tumor, between hiPC and macrophage clusters 0, 4, 12, and 14 in the tumor, and between htPC and microglia cluster 1 in non‐malignant tissue, albeit at a relatively low probability. Additionally, pericytes expressed CD4 and were predicted to respond to MHC‐II expression from antigen‐presenting cells.

In the communication with cluster 10 of monocytes, the tumor‐associated pericytes increased ECM, such as collagens and laminins, and immune‐related interactions, such as *ANXA1‐FPR1* and *IL1B‐CCR1*, associated with the malignant behavior of gliomas [70, 71, 72, 73], and therefore suggesting a more immunosuppressive pericyte phenotype. hiPC showed the most pronounced immune‐related signals (Fig. 7F).

A similar communication pattern was observed for the rest of macrophages and microglial clusters, with the exception of cluster 8 of monocytes, which displayed unique LR expression. Particularly, the main LR pairs in the normal tissue included interleukin 1b (*IL1B*) to the IL1 receptor 2 (*IL1R2*), interleukin‐8 (*CXCL8*), and Macrophage Inflammatory Protein 2‐Alpha (*CXCL2*) to the IL‐8 Receptor Type 2 (*CXCR2*), APP to the Sortilin‐related receptor 1 (*SORL1*) (Fig. 7E), involved in monocyte motility [74]. Binding to IL1R2 dampens IL1B pro‐inflammatory effects. Pericytes are known to support neutrophil transmigration via the *CXCL8‐CXCR2* pathway [75], suggesting that they might do the same with T cells. CXCL2‐CXCR2 is implicated in neutrophils recruitment and angiogenesis in tumors [76]. These pathways were taken over in the tumor environment by ECM pathways and the immune *ANXA1‐FPR1* and *FPR2*.

These results suggest that pericytes in the tumor environment increase functions related to matrix remodeling possibly modulating immune responses. Additionally, each pericyte subtype adapted its interaction profile in the tumor environment, with htPC and ecm‐PC increasing matrix and adhesion interactions, while hiPCs adopted an anti‐inflammatory profile.

## 4 Discussion

In this study, we delineate transcriptional and communication changes in mural cells depending on their proximity to the tumor. Cells residing in the border impact tumor cell proliferation, GBM invasiveness, and recurrence [77, 78], whereby we specifically investigate the role of pericytes as TME influencers. Our findings provide new important insights into the diverse roles of mural cells within the GBM microenvironment.

In our datasets, the abundance of mural cell subclusters varied depending on the region. In the mouse model, signaling pericytes dominated the tumor core, whereas immune pericytes were absent. In humans, transport pericytes in normal tissue switched to matrix‐associated and immune pericytes within the tumor. This transport‐to‐matrix switch in pericytes may suggest a transition to a more immature, but also more actively signaling state. A similar result was recently described in GBM by Xie *et al*. [79], who reported that, while pericytes expressing transport‐related genes were the majority in normal tissue, a distinct pericyte cluster emerged in GBM characterized by upregulated ECM‐related genes [79].

An immune pericyte cluster was found in both mouse and human datasets sharing upregulated genes and functions with previously reported mural cells immune cluster [79, 80]. In the human dataset analyzed here, immune pericytes expressed cytokines, complement, and MHC‐II‐related genes, critical for antigen presentation and immune activation, consistent with the “scavenging” profile described in prior studies [80]. Human immune pericytes also participated in anti‐inflammatory signaling toward macrophages and T cells in the tumor core, suggesting a mixed pro‐ and anti‐inflammatory phenotype.

The mouse immune pericytes expressed *Ccl19* and *Apoe*, similarly to what was described previously [79]. Ccl19 is a potent chemoattractant for T cells, B cells, DC, and NK cells [81, 82, 83, 84, 85], and it has been linked to reduced tumor burden in lung cancer [85, 86]. Furthermore, treatment with IL‐7/CCL19‐expressing CAR‐T cells has been shown to induce extensive T‐cell infiltration and achieve complete rejection of GBM [87], underscoring the immune‐modulating potential of the mouse immune pericyte cluster. Apoe regulates lipid and amyloid‐beta metabolism, neuroinflammation, tau phosphorylation [88], changes in cerebral blood flow, vascular pathology, and BBB breakdown [89, 90] and its expression is enhanced in GBM [67]. Apoe expression implies pericytes involvement in lipid transport and metabolism in the TME.

Despite the fact that both mouse and human immune pericytes exhibited increased functions related to the regulation, both positive and negative, of immune processes, they shared only 4–5% of their marker genes. Additionally, their spatial distribution differed significantly. In mice, the immune subcluster was found in the contralateral and border regions but was absent in the tumor core, whereas in humans, it was predominantly tumor‐associated. This divergence suggests that although the clusters share immune‐modulating functions, they may play distinct roles in the TME, potentially adapting their functions to the spatial and species‐specific context. Species‐specific variations in pericyte subtypes and gene expression are consistent with previous studies emphasizing species‐specific gene expression patterns [53, 54, 55, 91, 92, 93], which likely reflect intrinsic variations in vascular architecture and immune responses between species.

In the mouse contralateral region, pericytes primarily supported cellular biosynthesis, metabolism, and development, and human pericytes showed functions associated with cellular component biogenesis and transport [79]. Interestingly, mural cells at the tumor border in the mouse model showed downregulation of genes associated with reparative and supportive functions, such as autophagy and wound healing. This suggests that border mural cells may represent a transitional state, adapting to the dynamic interplay between tumor‐promoting and tumor‐suppressing signals. Tumor‐resident pericytes exhibited strong activation and significant upregulation of genes linked to vesicle trafficking, endocytosis, catabolism, and protein secretion in the mouse model, and to collagen production and ECM remodeling in the human dataset. These findings suggest that mural cells in GBM change their gene expression and functions according to their spatial vicinity to the tumor.

We further identified 228 shared DEGs in mural cells from the mouse and human datasets. FGSEA analysis revealed that these genes were associated with RNA processing and the suppression of catabolic processes, suggesting conserved mechanisms by which mural cells contribute to the TME.

Although not among the pathways obtained from GO:BP analysis, several of the shared DEGs have been implicated in immune‐related processes (*Fosl2*, *Nfkbia*, *Nfkbiz*, *Ier3*, *Ifnar1*, *Irf1*, *Litaf*, *Socs3*, and *Usp14*) [94, 95, 96, 97, 98, 99, 100, 101] and chaperone‐mediated autophagy (CMA) (*Lamp2*, *Hspa8*, *Ctsb*, *Calr*, *Ddit4*, *Psap*, *Vmp1*) [102, 103, 104, 105, 106]. Previous studies have shown that pericytes in GBM aberrantly upregulate CMA, which alters their secretome and contributes to an immunosuppressive TME [24]. Furthermore, CMA activity in pericytes correlates with both peritumoral parenchymal invasion and poor patient outcomes [107]. Interestingly, our findings suggest that tumor core pericytes suppress global catabolism while upregulating multiple CMA‐related genes. This paradox is consistent with reports showing that CMA can be selectively activated to degrade stress, metabolic, and immune‐related proteins, even when overall catabolism is reduced [108, 109]. Such selective reprogramming may help pericytes adapt to the TME by preserving key functions while modulating immune responses and secretion.

Additionally, many of the shared upregulated genes were related to ECM remodeling (*Col4a2*, *Colgalt1*, *Lama4*, *Adamts1*, *Crispld2*, *Olfml2a*, *Plod2*, *Thbs1*). This is consistent with recent studies demonstrating the benefits of blocking collagen synthesis in a mouse model of GBM, where the blockage of the collagen crosslinking enzyme Lysyl oxidase homolog 2 enhanced chemotherapy efficacy in both murine and human patient‐derived xenograft GBM models [79]. Also, ECM components secreted by pericytes at the tumor edge have been suggested as prognostic markers in GBM, both in mouse and human [107]. These findings emphasize the potential of therapies targeting mural cell‐secreted ECM to mitigate GBM progression and reduce tumor invasiveness.

For a long time, pericytes in GBM have been primarily studied for their angiogenic properties, while other potentially important functions of pericytes in GBM have been neglected. However, recent studies have highlighted immune‐related roles of pericytes in GBM, including antigen presentation and phagocytic activity, as well as their ability to shape the tumor microenvironment [13, 22, 24, 110, 111].

Here, we consolidate those findings showing that mural cells engage in distinct ligand–receptor interactions with specific immune cell types, whereby their interaction strength varies by spatial localization.

We demonstrate that in both mouse and human, tumor‐associated pericytes highly communicated particularly with TAMs. Furthermore, pericytes exhibited a functional switch in their interactions with T cells, suppressing MHC‐I antigen presentation, implying a loss of antigen‐presenting capacity. This aligns with previous studies showing that pericytes, upon contact from GBM cells, increased the production of anti‐inflammatory cytokines IL‐10 and TGF‐β and secretion of other immunosuppressive factors and interleukins connected to metastasis [22, 112, 113], together with a reduction of CD80, CD86, and MHC‐II expression. GBM‐conditioned pericytes also showed an impaired ability to activate T cells [22, 114]. This reinforces the immunosuppressive phenotype of tumor pericytes and may potentially promote alternative T‐cell functions, such as angiogenesis [115] in the tumor core. These alterations could reflect an orchestrated adaptation of tumor pericytes to promote immune cell adhesion and priming.

We observed species‐specific differences also in signaling pathways. In human GBM, ECM pathways dominated the signaling landscape to immune cells, whereas in the mouse model, ECM signaling was mixed with vascular and immune‐related pathways. These findings align with prior reports, which observed an increase in ECM‐related genes in human GBM [79] while in the mouse an increase in genes related to other signatures (vascular, immune and macrophage ones), not related to ECM was described [116].

Finally, while GL261 recapitulates key features of GBM and enables the use of immunocompetent mice to specifically study host immune responses to tumor cells, species‐specific differences between mouse and human in tumor biology may limit the translational relevance of results. It is plausible that some of the differences that we observed in mural cell populations between mice and humans could depend on the choice of model to study the tumor and immune cells in the TME. Although a few studies have reported orthotopic xenografts of human GBM in immunocompetent mice [22, 24, 113], in our experience, human GBM cells are rapidly rejected in immunocompetent mice. Other approaches, enabling tumor growth through T‐cell suppression [117], compromise the immune dynamics we aimed to study. Since our study specifically focused on pericyte‐immune crosstalk, including MHC expression, as well as tumor border characteristics, we decided that a murine glioma model was appropriate. This model allowed us to investigate tumor‐pericyte‐immune cell interactions within a biologically relevant microenvironment while enabling direct comparison with human specimens. Future studies adopting diverse techniques could provide deeper insights into mural cell responses in human GBM.

## 5 Conclusions

Our study underscores the dynamic and region‐specific roles of pericytes within the GBM microenvironment. Tumor‐ and border‐resident mural cells exhibited transcriptional changes indicative of functional adaptations in both mouse and human datasets. Border‐residing mural cells reflected a transitional phenotype. Conversely, tumor‐residing mural cells exhibited transcriptional changes indicating high protein secretion and signaling. Communication analysis indicated enhanced signaling activity towards immune cells, in particular macrophages, monocytes, and DCs, with distinct region‐ and subtype‐specific signaling patterns influencing immune modulation. A general switch in signaling toward adhesion and ECM interactions was observed, accompanied by a diminished antigen presentation to T cells. These insights not only deepen our understanding of the immune landscape in GBM but also suggest potential targets for disrupting mural cell communication, paving the way for novel therapeutic strategies.

## Conflict of interest

The authors declare no conflict of interest.

## Author contributions

Conceptualization: CB, RC, GP; performed the experiments: CB, CG, GC, RC; data analysis: CB, RC; interpretation: CB, RC, GP; JB; writing– original draft preparation: CB; writing– review and editing: CB, RC, GP, JB; funding acquisition: RC, GP; materials and resources: JB, GP.

## Peer review

The peer review history for this article is available at https://www.webofscience.com/api/gateway/wos/peer‐review/10.1002/1878‐0261.70095.

## Supporting information


**Fig. S1.** Percentage distribution of mouse cell clusters, pericytes markers, and mural cell distribution. Proportion of cells within each cluster across the three regions: contralateral (green), border (magenta), and tumor (blue) for the mouse dataset. Bar lengths represent the average percentage per cluster, with each dot corresponding to an individual mouse. Error bars indicate SD. The abundance of each cell cluster was calculated and normalized in percentage for the total cells obtained from each sample. Statistical significance was calculated with two‐way ANOVA followed by Tukey's *post hoc* comparison. *P < 0.05; **P < 0.01; ***P < 0.001. (B) Dot plot displaying the expression of the pericytes enriched markers from Oudenaarden et al. [1]. Size of the dots represents the percentage of cells expressing a specific gene, while the color represents the average expression of the gene for that percentage of cells. (C) Line plot showing relative distribution of the mural cell clusters. The dots connected by the lines represent the average relative percentage of a specific subcluster in contra, border or tumor for each sample. Dashed lines represent SD. Colors indicate the subclusters. (D) Proportion of cells within each cluster across the three regions. Bar lengths represent the average percentage per cluster, with each dot corresponding to an individual mouse. Error bars indicate SD. SD, standard deviation; DC, dendritic cells; NK, natural killer; PC, pericytes; EC, endothelial cells; Oligo, oligodendrocytes; NPC, neural precursor cells; CPC, choroid plexus cells; mtPC, mouse transport pericytes; msPC, mouse signaling pericytes; cSMC, classical smooth muscle cells, rSMC, reactive smooth muscle cells; miPC, mouse immune pericytes. [1] Oudenaarden, C., Sjolund, J. & Pietras, K. (2022) Upregulated functional gene expression programmes in tumour pericytes mark progression in patients with low‐grade glioma, Mol Oncol. 16, 405–421.

**Fig. S2.** Mouse microglia and macrophages DEGs and associated pathways. (A) Heatmap of the top 10 upregulated DEGs for mouse microglia and macrophages compared to the other microglia and macrophages clusters. (B) Top 10 GO terms for each cluster are noted in boxed sections. DEGs, differentially expressed genes; avg log_2_FC, average log_2_ fold change; NES, normalized enrichment score.

**Fig. S3.** Mouse and human immune cells classification based on Pombo Antunes et al. 2021. Dot plots displaying the expression of top 20 markers of (A) TAM, (B) dendritic cells, (C) T regulatory signature in the mouse dataset, and (D) TAM signature in the human dataset. Size of the dots represents the % of cells expressing a specific gene, while the color represents the average expression of the gene for that percentage of cells. TAM, tumor‐associated macrophages; prolif. TAM, proliferating TAM; DC, dendritic cells; pre‐DC, DC precursor; T reg, T regulatory cells; avg expr., average expression.

**Fig. S4.** Mouse T cells DEGs and associated pathways. (A) Heatmap of the top 10 upregulated DEGs for mouse T cells compared to the other T‐cell clusters. (B) Top 10 GO:BP terms for each cluster are noted in boxed sections. DEGs, differentially expressed genes; GO:BP, Gene Ontology Biological Processes; avg log2FC, average log2 fold change; NES, normalized enrichment score; T cells prolif, proliferating T cells.

**Fig. S5.** CCL19, ANGPT2, and APOE are selectively expressed in mouse immune pericytes compared to other mural subtypes. Representative confocal images show that only a subset of CD13^+^/αSMA^+^ cells co‐express CCL19 (panel B, subpanel 1), ANGPT2 (panel C, subpanel 1), or APOE (panel E, subpanel 1). These cells are identified as immune PCs. In contrast, CD13^+^/αSMA^+^ cells lacking expression of CCL19 (panel A, subpanel 2), ANGPT2 (panel D, subpanel 2), and APOE (panel E, subpanel 2) are classified as smooth muscle cells. CD13^+^/αSMA^−^ pericytes also do not express CCL19 (panel B, subpanel 3), ANGPT2 (panel D, subpanel 3), or APOE (panel E, subpanel 3). Scale bars: 50 μm overview panels; 20 μm zoomed panels. CCL19, chemokine ligand 19; ANGPT2, angiopoietin‐2; APOE, apolipoprotein E; CD13, aminopeptidase N (pericyte marker); αSMA, alpha smooth muscle actin (smooth muscle cells marker).

**Fig. S6.** Human dataset characterization of cell populations in the different regions. (A) UMAP visualization of cells, categorized into distinct clusters and (B) divided by tumor core and non‐malignant regions. The UMAPs are colored by cluster. (C) Bar plot illustrating the relative distribution of each cell type in each region. Green, non‐malignant; blue, tumor. (D) Proportion of cells within each cluster across the two regions. Bar lengths represent the average percentage per cluster, with each dot corresponding to an individual patient. Error bars indicate standard deviation. The abundance of each cell cluster was calculated and normalized in percentage for the total cells obtained from each sample. Statistical significance was calculated with two‐way ANOVA followed by Tukey's post hoc comparison. *P < 0.05; **P < 0.01; ***P < 0.001. (E) Dot plot depicting the expression of genes used to define cell types. Size of the dots represents the % of cells expressing a specific gene, while the color represents the average expression of the gene for that percentage of cells. (F) Dot plot displaying the expression of the pericytes enriched markers from Oudenaarden et al. 2021. (G) Line plot showing the relative distribution of the mural cell clusters. The dots connected by the lines represent the average relative percentage of a specific subcluster in tumor or non‐malignant area for each patient. Dashed lines represent SD. Colors indicate the subclusters. (H) Proportion of cells within each cluster across the two regions. Bar lengths represent the average percentage per cluster, with each dot corresponding to an individual patient. Error bars indicate SD. SD, standard deviation; TAM, tumor‐associated macrophages; prolif. TAM, proliferating TAM; PC, pericytes; EC, endothelial cells; MC, mural cells; hiPC, human immune PC; htPC, human transport PC; ecm‐PC, extracellular matrix PC; avg, average.

**Fig. S7.** Comparison of mural cells with previous literature illustrated through dot plots. Specifically, (A) mouse mural cells divided by subclusters, (B) mouse mural cells divided by region, (C) human mural cells divided by subclusters, and (D) human mural cells divided by region. Size of the dots represents the % of cells expressing a specific gene, while the color represents the average expression of the gene for that percentage of cells. PC, pericytes; SMC, smooth muscle cells; mtPC, mouse transport pericytes; msPC, mouse signaling pericytes; cSMC, classical smooth muscle cells, rSMC, reactive smooth muscle cells; miPC, mouse immune pericytes; hiPC, human immune PC; htPC, human transport PC; ecm‐PC, extracellular matrix PC; avg expr., average expression.

**Fig. S8.** Cell–cell communication intensity scatterplots. Scatterplots showing the mouse (A) or human (B) total outgoing or incoming communication probability interaction strength for each cell population in the dataset across the contralateral region, border, and tumor (mouse) and non‐malignant and tumor (human). Dot sizes represent the number of inferred connections (outgoing and incoming). Pericytes subclusters are highlighted in red. mtPC, mouse transport pericytes; msPC, mouse signaling pericytes; cSMC, classical smooth muscle cells, rSMC, reactive smooth muscle cells; miPC, mouse immune pericytes. hiPC, human immune pericytes; htPC, human transport pericytes; ecm‐PC, extracellular matrix pericytes; TAM, tumor‐associated macrophages; prolif. TAM, proliferating TAM; DC, dendritic cells; T cells prolif, proliferating T cells; Neutro, neutrophils.

**Table S1.** Mouse sequencing data and cell numbers.

**Table S2.** Table showing the top 15 upregulated DEGs that characterize each cell population identified in the mouse dataset.

**Table S3.** Table listing the top 15 upregulated DEGs that characterize each cell population identified in the human dataset.

**Table S4.** (A) Table listing the DEGs between mural cells in the tumor core and the contralateral hemisphere. (B) Table listing the upregulated DEGs in human mural cells in the tumor core vs non‐malignant region. (C) Table listing the 228 shared genes between mouse and human mural cells.

## Data Availability

Raw data and code supporting the findings of this study are openly available upon publication in Gene Expression Omnibus (PRJNA1259731).
