# Supplementary figures and images for: Pericytes change function depending on glioblastoma vicinity: emphasis on immune regulation
Source: Mol Oncol. 2025 Jul 17;19(9):2491–514. doi: 10.1002/1878-0261.70095 (PMC12420362; doi:10.1002/1878-0261.70095)

Suppl. Figure 1

A

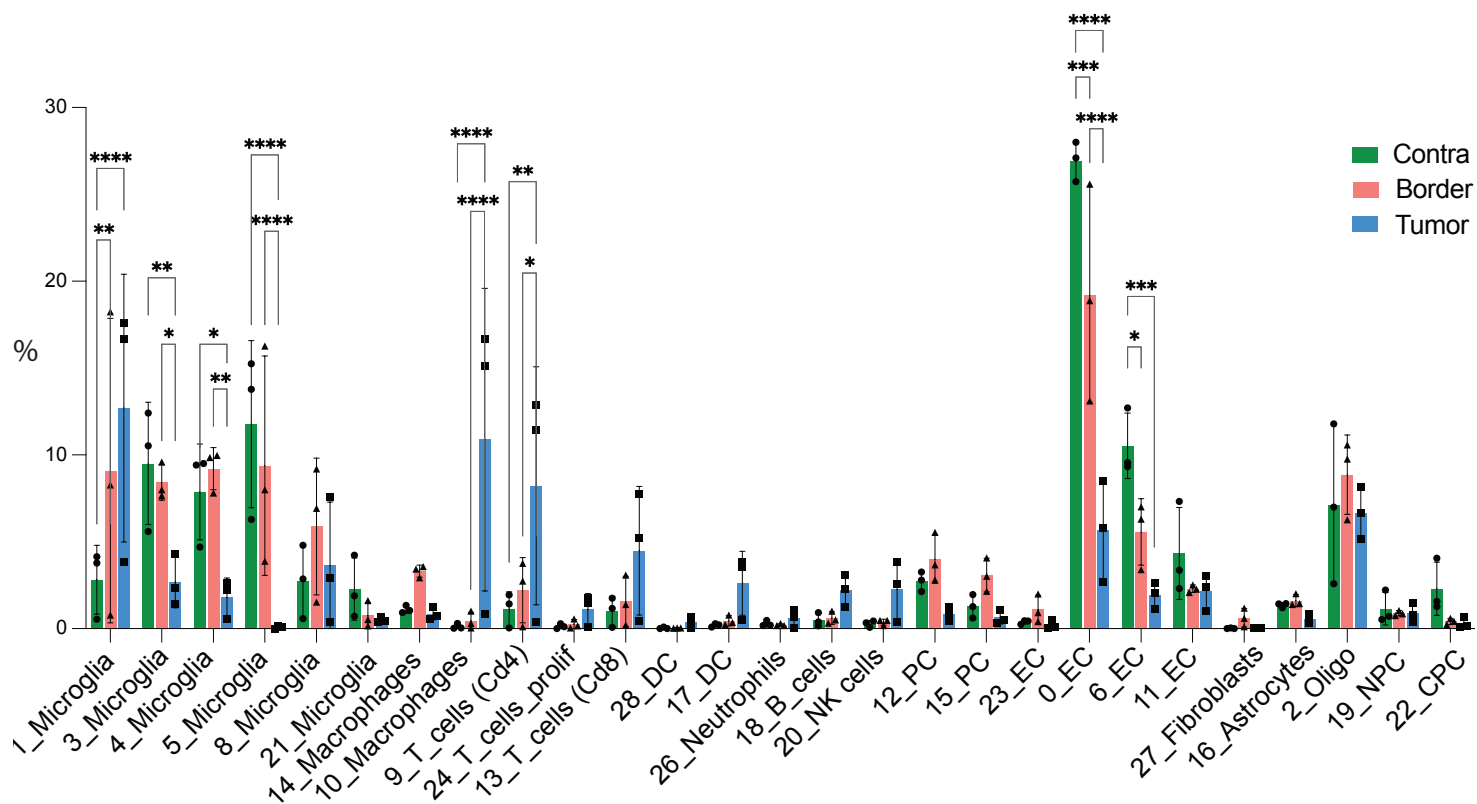

B

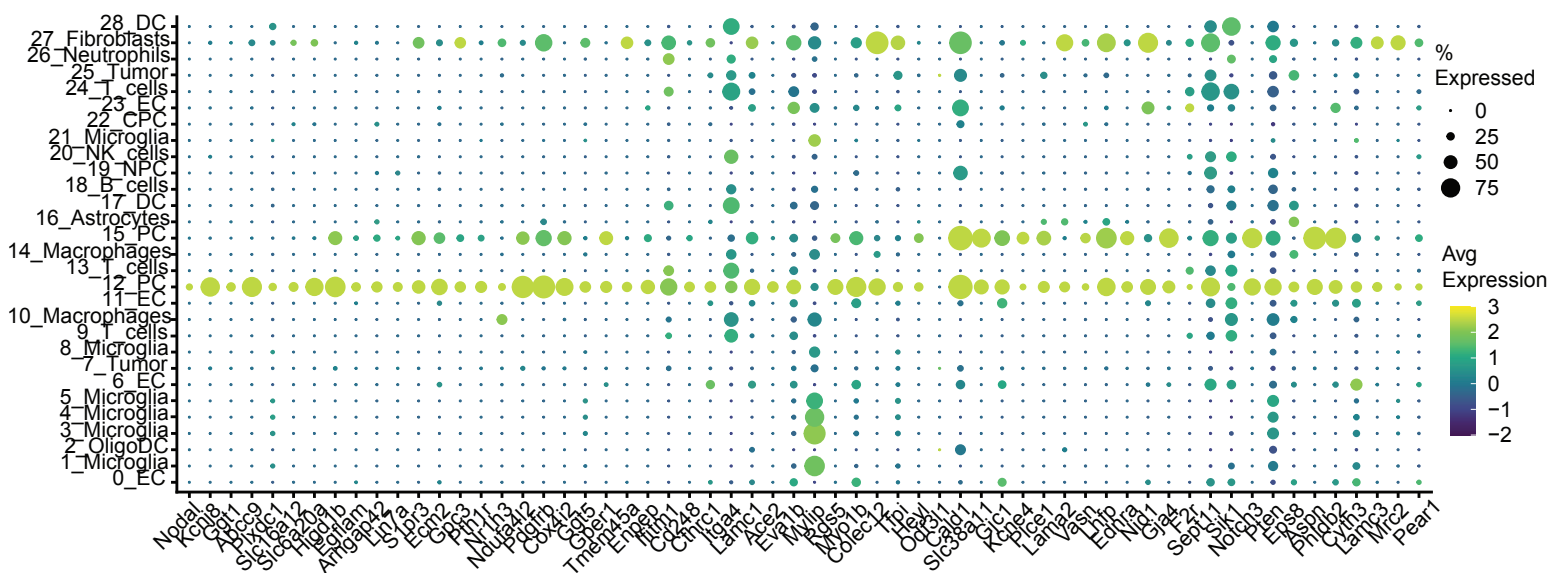

C

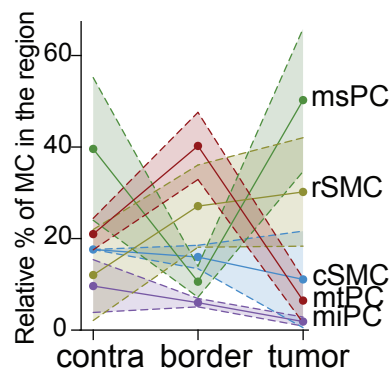

D

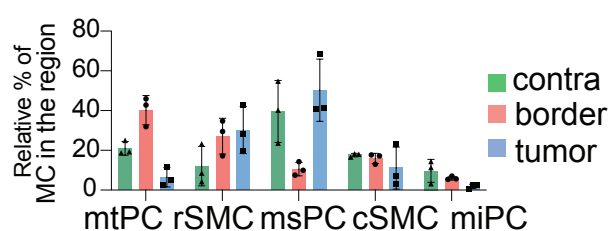

Supplement: Supplementary file 1 — Fig. S1. Percentage distribution of mouse cell clusters, pericytes markers, and mural cell distribution. Proportion of cells within each cluster across the three regions: contralateral (green), border (magenta), and tumor (blue) for the mouse dataset. Bar lengths represent the average percentage per cluster, with each dot corresponding to an individual mouse. Error bars indicate SD. The abundance of each cell cluster was calculated and normalized in percentage for the total cells obtained from each sample. Statistical significance was calculated with two‐way ANOVA followed by Tukey's post hoc comparison. *P < 0.05; **P < 0.01; ***P < 0.001. (B) Dot plot displaying the expression of the pericytes enriched markers from Oudenaarden et al. [1]. Size of the dots represents the percentage of cells expressing a specific gene, while the color represents the average expression of the gene for that percentage of cells. (C) Line plot showing relative distribution of the mural cell clusters. The dots connected by the lines represent the average relative percentage of a specific subcluster in contra, border or tumor for each sample. Dashed lines represent SD. Colors indicate the subclusters. (D) Proportion of cells within each cluster across the three regions. Bar lengths represent the average percentage per cluster, with each dot corresponding to an individual mouse. Error bars indicate SD. SD, standard deviation; DC, dendritic cells; NK, natural killer; PC, pericytes; EC, endothelial cells; Oligo, oligodendrocytes; NPC, neural precursor cells; CPC, choroid plexus cells; mtPC, mouse transport pericytes; msPC, mouse signaling pericytes; cSMC, classical smooth muscle cells, rSMC, reactive smooth muscle cells; miPC, mouse immune pericytes. [1] Oudenaarden, C., Sjolund, J. & Pietras, K. (2022) Upregulated functional gene expression programmes in tumour pericytes mark progression in patients with low‐grade glioma, Mol Oncol. 16, 405–421. [file MOL2-19-2491-s011.pdf]

Suppl. Figure 2

A DEGs mouse microglia and macrophages

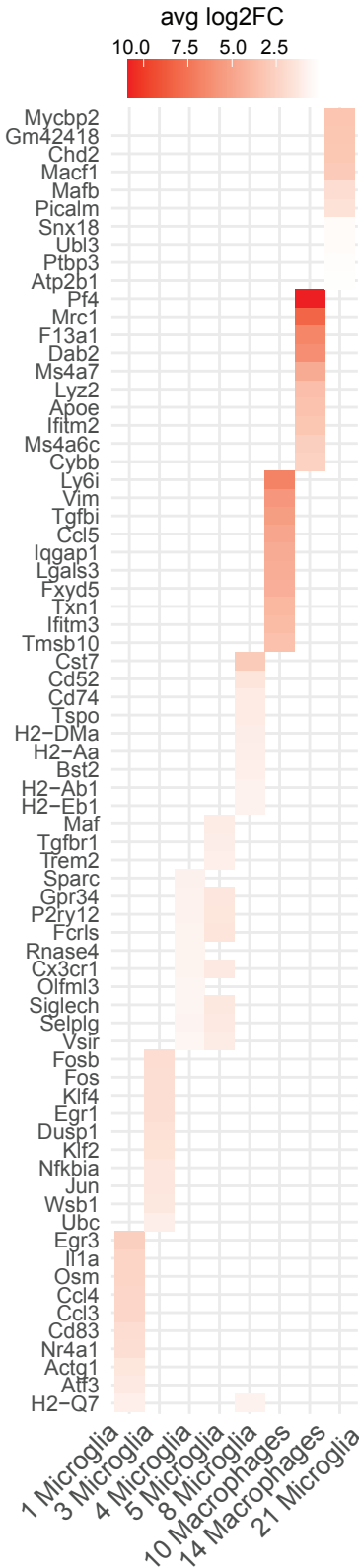

B

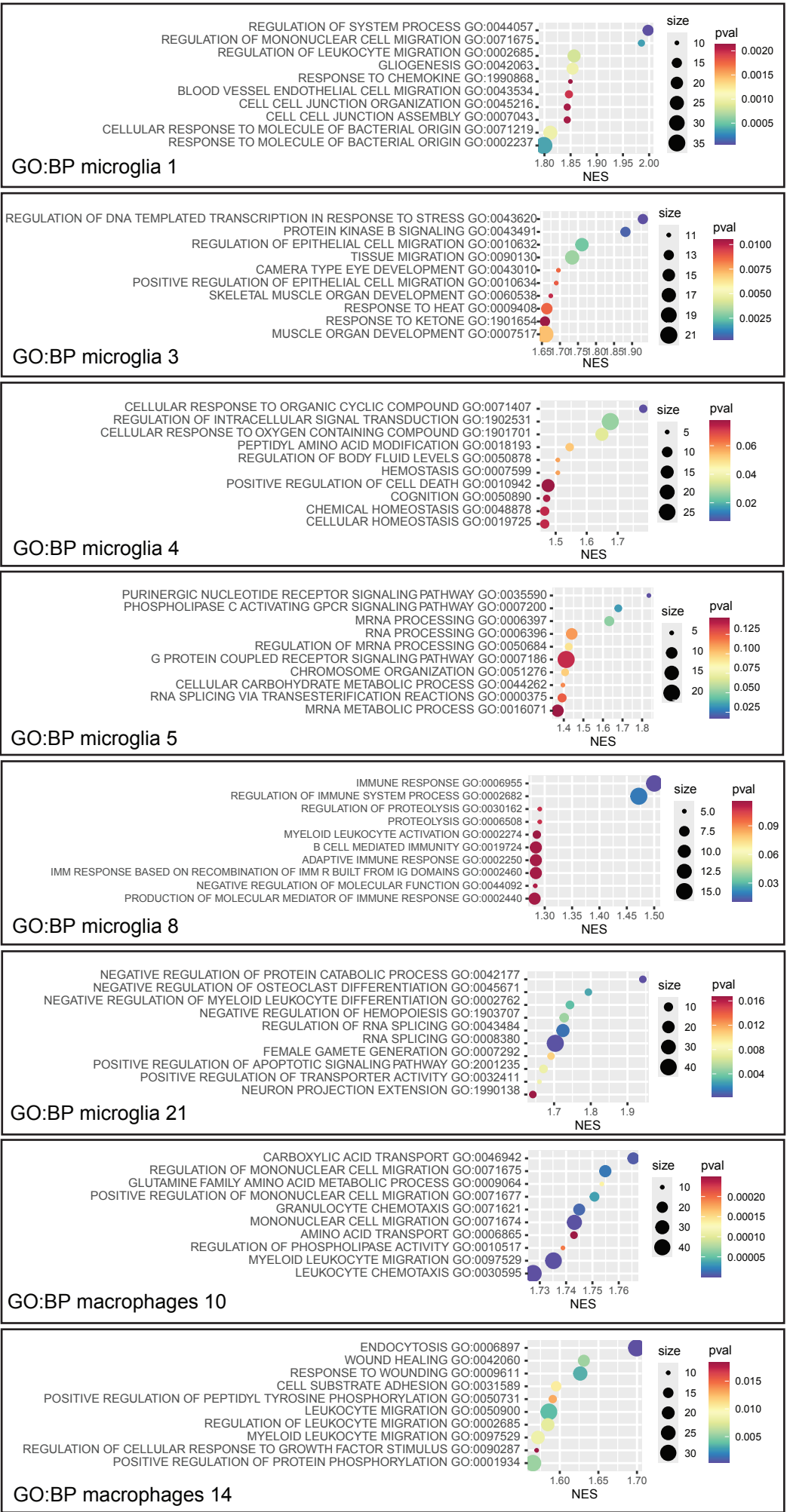

Supplement: Supplementary file 2 — Fig. S2. Mouse microglia and macrophages DEGs and associated pathways. (A) Heatmap of the top 10 upregulated DEGs for mouse microglia and macrophages compared to the other microglia and macrophages clusters. (B) Top 10 GO terms for each cluster are noted in boxed sections. DEGs, differentially expressed genes; avg log2FC, average log2 fold change; NES, normalized enrichment score. [file MOL2-19-2491-s009.pdf]

Suppl. Figure 3

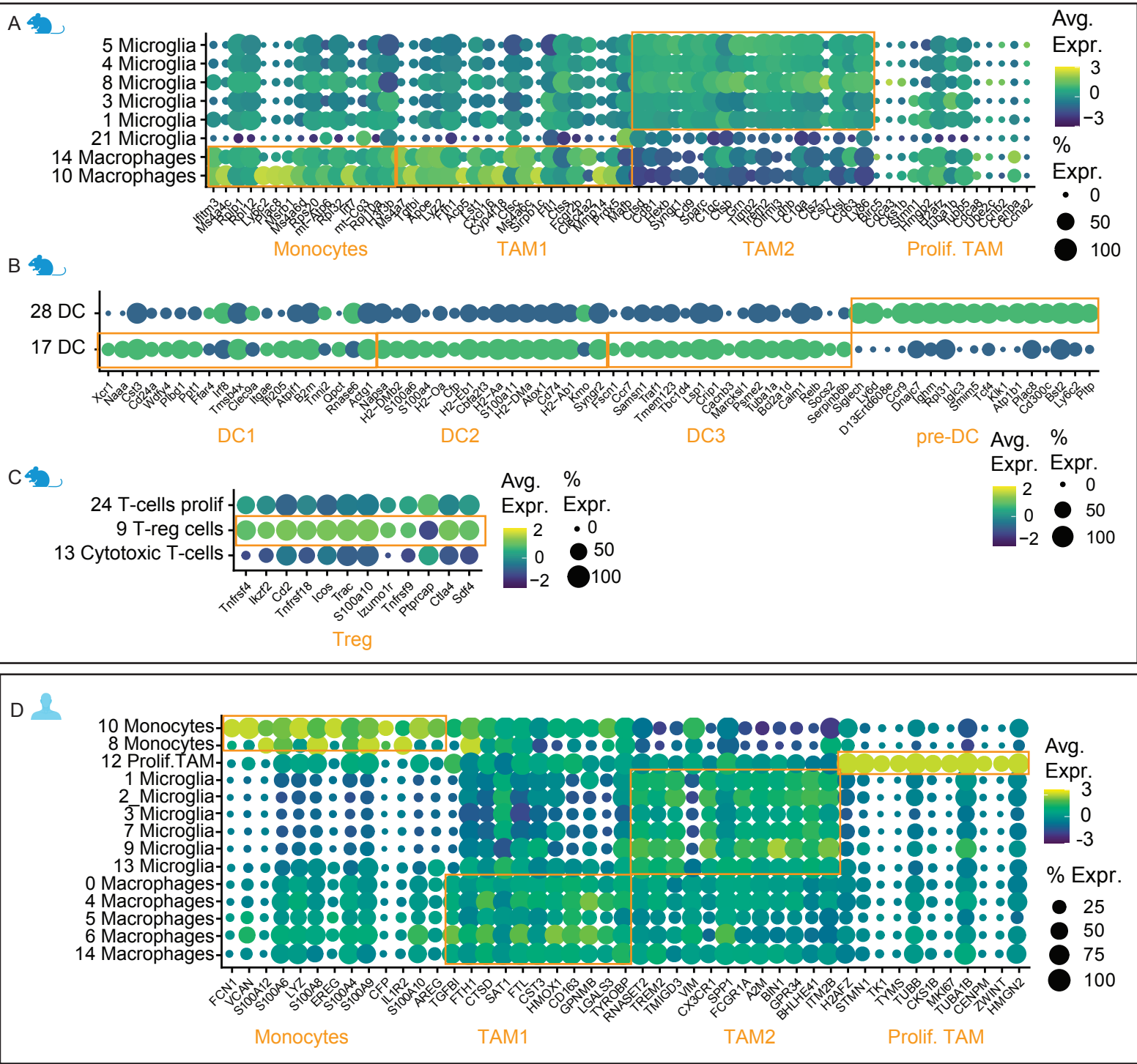

Supplement: Supplementary file 3 — Fig. S3. Mouse and human immune cells classification based on Pombo Antunes et al. 2021. Dot plots displaying the expression of top 20 markers of (A) TAM, (B) dendritic cells, (C) T regulatory signature in the mouse dataset, and (D) TAM signature in the human dataset. Size of the dots represents the % of cells expressing a specific gene, while the color represents the average expression of the gene for that percentage of cells. TAM, tumor‐associated macrophages; prolif. TAM, proliferating TAM; DC, dendritic cells; pre‐DC, DC precursor; T reg, T regulatory cells; avg expr., average expression. [file MOL2-19-2491-s007.pdf]

Suppl. Figure 4

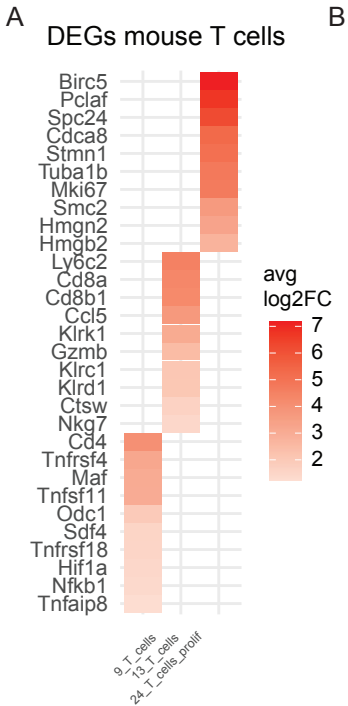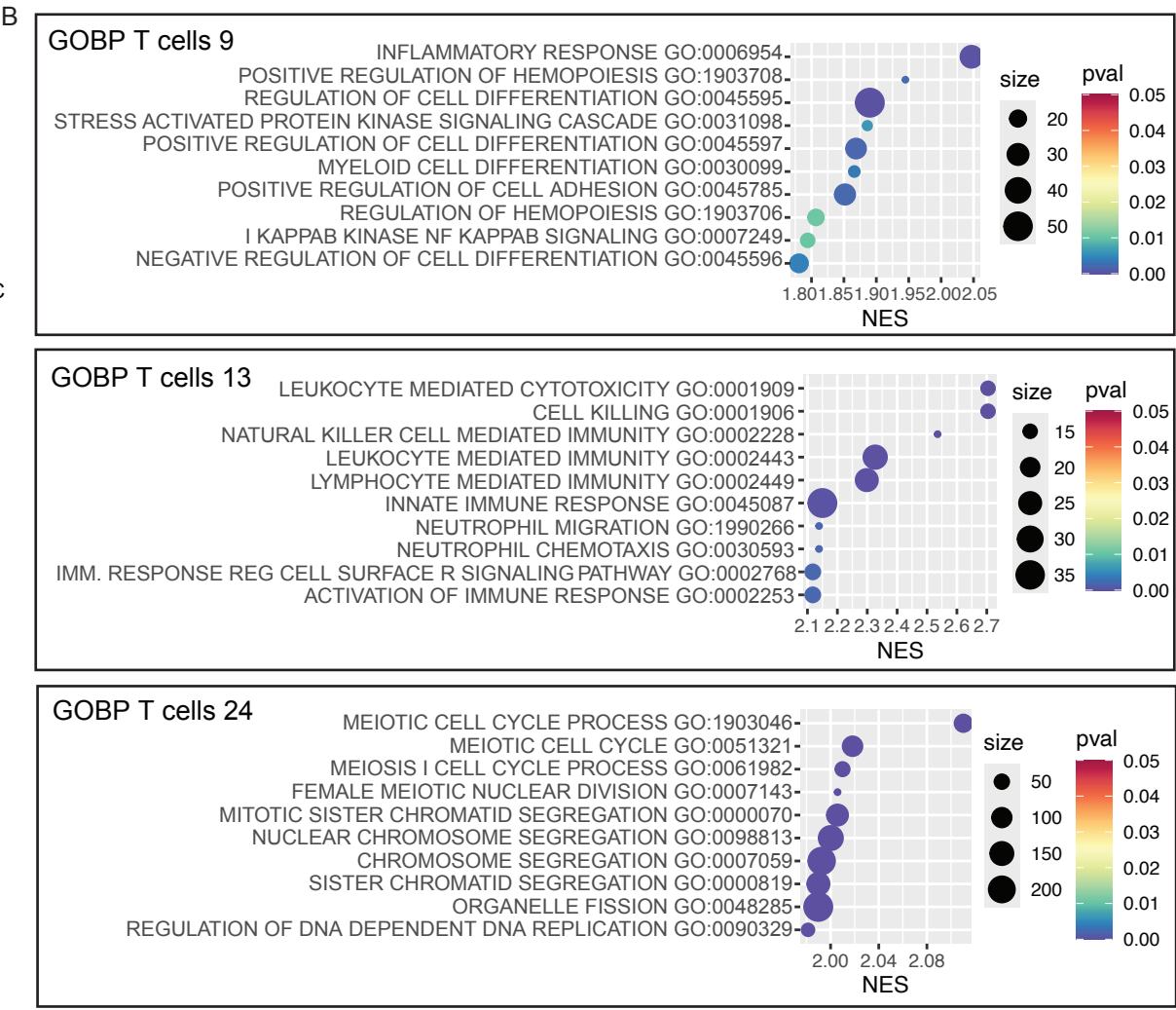

Supplement: Supplementary file 4 — Fig. S4. Mouse T cells DEGs and associated pathways. (A) Heatmap of the top 10 upregulated DEGs for mouse T cells compared to the other T‐cell clusters. (B) Top 10 GO:BP terms for each cluster are noted in boxed sections. DEGs, differentially expressed genes; GO:BP, Gene Ontology Biological Processes; avg log2FC, average log2 fold change; NES, normalized enrichment score; T cells prolif, proliferating T cells. [file MOL2-19-2491-s002.pdf]

Suppl. Figure 5

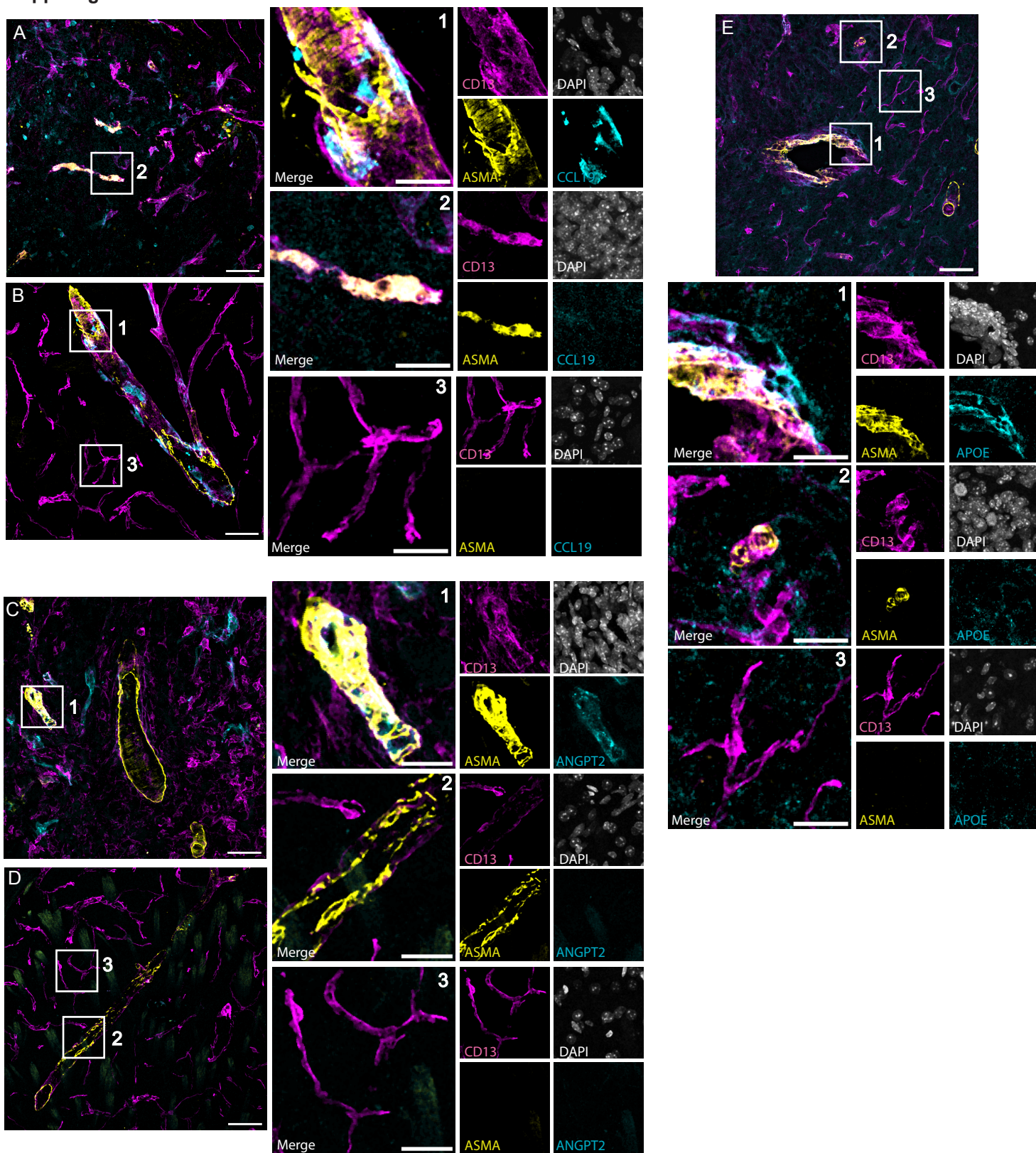

Supplement: Supplementary file 5 — Fig. S5. CCL19, ANGPT2, and APOE are selectively expressed in mouse immune pericytes compared to other mural subtypes. Representative confocal images show that only a subset of CD13+/αSMA+ cells co‐express CCL19 (panel B, subpanel 1), ANGPT2 (panel C, subpanel 1), or APOE (panel E, subpanel 1). These cells are identified as immune PCs. In contrast, CD13+/αSMA+ cells lacking expression of CCL19 (panel A, subpanel 2), ANGPT2 (panel D, subpanel 2), and APOE (panel E, subpanel 2) are classified as smooth muscle cells. CD13+/αSMA− pericytes also do not express CCL19 (panel B, subpanel 3), ANGPT2 (panel D, subpanel 3), or APOE (panel E, subpanel 3). Scale bars: 50 μm overview panels; 20 μm zoomed panels. CCL19, chemokine ligand 19; ANGPT2, angiopoietin‐2; APOE, apolipoprotein E; CD13, aminopeptidase N (pericyte marker); αSMA, alpha smooth muscle actin (smooth muscle cells marker). [file MOL2-19-2491-s012.pdf]

A

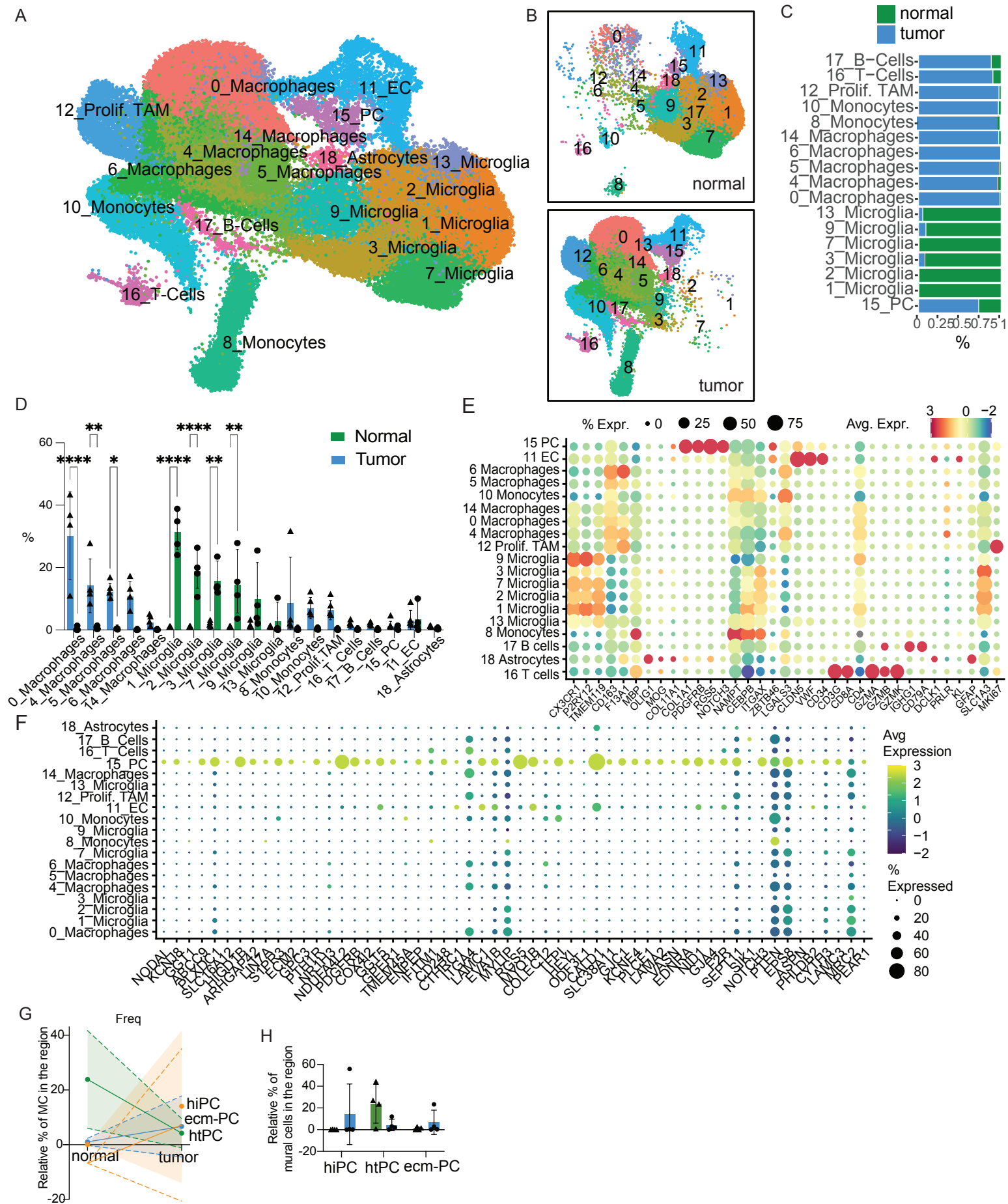

Supplement: Supplementary file 6 — Fig. S6. Human dataset characterization of cell populations in the different regions. (A) UMAP visualization of cells, categorized into distinct clusters and (B) divided by tumor core and non‐malignant regions. The UMAPs are colored by cluster. (C) Bar plot illustrating the relative distribution of each cell type in each region. Green, non‐malignant; blue, tumor. (D) Proportion of cells within each cluster across the two regions. Bar lengths represent the average percentage per cluster, with each dot corresponding to an individual patient. Error bars indicate standard deviation. The abundance of each cell cluster was calculated and normalized in percentage for the total cells obtained from each sample. Statistical significance was calculated with two‐way ANOVA followed by Tukey's post hoc comparison. *P < 0.05; **P < 0.01; ***P < 0.001. (E) Dot plot depicting the expression of genes used to define cell types. Size of the dots represents the % of cells expressing a specific gene, while the color represents the average expression of the gene for that percentage of cells. (F) Dot plot displaying the expression of the pericytes enriched markers from Oudenaarden et al. 2021. (G) Line plot showing the relative distribution of the mural cell clusters. The dots connected by the lines represent the average relative percentage of a specific subcluster in tumor or non‐malignant area for each patient. Dashed lines represent SD. Colors indicate the subclusters. (H) Proportion of cells within each cluster across the two regions. Bar lengths represent the average percentage per cluster, with each dot corresponding to an individual patient. Error bars indicate SD. SD, standard deviation; TAM, tumor‐associated macrophages; prolif. TAM, proliferating TAM; PC, pericytes; EC, endothelial cells; MC, mural cells; hiPC, human immune PC; htPC, human transport PC; ecm‐PC, extracellular matrix PC; avg, average. [file MOL2-19-2491-s004.pdf]

Suppl. Figure 8

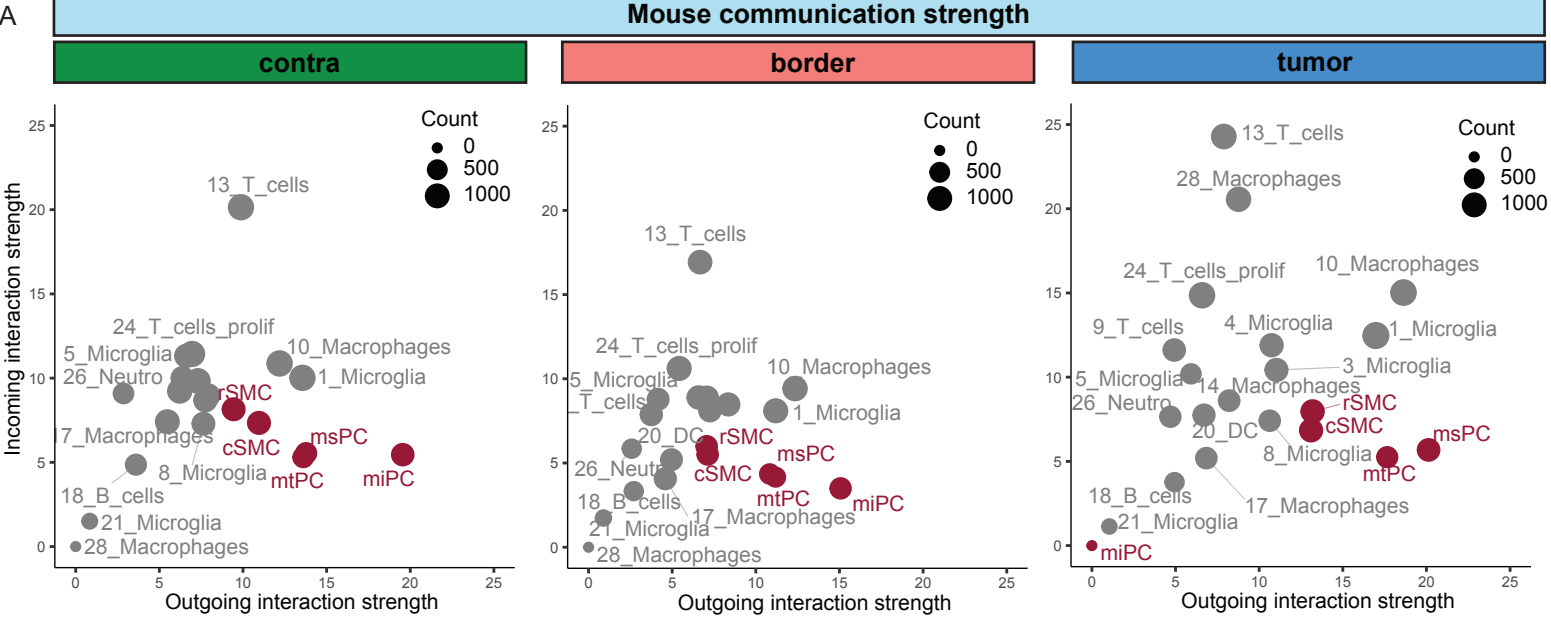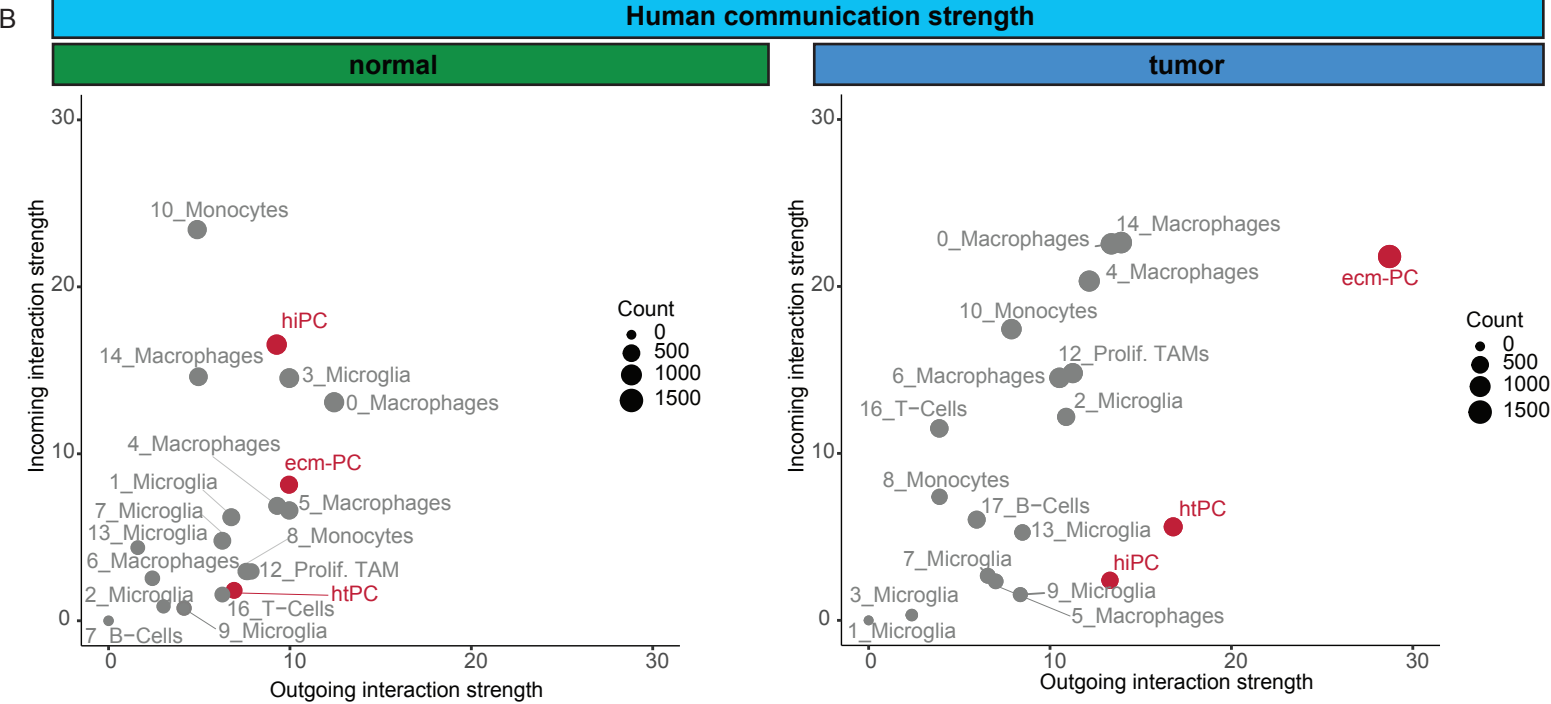

Supplement: Supplementary file 8 — Fig. S8. Cell–cell communication intensity scatterplots. Scatterplots showing the mouse (A) or human (B) total outgoing or incoming communication probability interaction strength for each cell population in the dataset across the contralateral region, border, and tumor (mouse) and non‐malignant and tumor (human). Dot sizes represent the number of inferred connections (outgoing and incoming). Pericytes subclusters are highlighted in red. mtPC, mouse transport pericytes; msPC, mouse signaling pericytes; cSMC, classical smooth muscle cells, rSMC, reactive smooth muscle cells; miPC, mouse immune pericytes. hiPC, human immune pericytes; htPC, human transport pericytes; ecm‐PC, extracellular matrix pericytes; TAM, tumor‐associated macrophages; prolif. TAM, proliferating TAM; DC, dendritic cells; T cells prolif, proliferating T cells; Neutro, neutrophils. [file MOL2-19-2491-s008.pdf]
