# Supplementary material for: Pericytes change function depending on glioblastoma vicinity: emphasis on immune regulation
Source: Mol Oncol. 2025 Jul 17;19(9):2491–514. doi: 10.1002/1878-0261.70095 (PMC12420362; doi:10.1002/1878-0261.70095)
Supplement: Supplementary file 7 — Fig. S7. Comparison of mural cells with previous literature illustrated through dot plots. Specifically, (A) mouse mural cells divided by subclusters, (B) mouse mural cells divided by region, (C) human mural cells divided by subclusters, and (D) human mural cells divided by region. Size of the dots represents the % of cells expressing a specific gene, while the color represents the average expression of the gene for that percentage of cells. PC, pericytes; SMC, smooth muscle cells; mtPC, mouse transport pericytes; msPC, mouse signaling pericytes; cSMC, classical smooth muscle cells, rSMC, reactive smooth muscle cells; miPC, mouse immune pericytes; hiPC, human immune PC; htPC, human transport PC; ecm‐PC, extracellular matrix PC; avg expr., average expression. [file MOL2-19-2491-s006.pdf]

Suppl. Figure 7

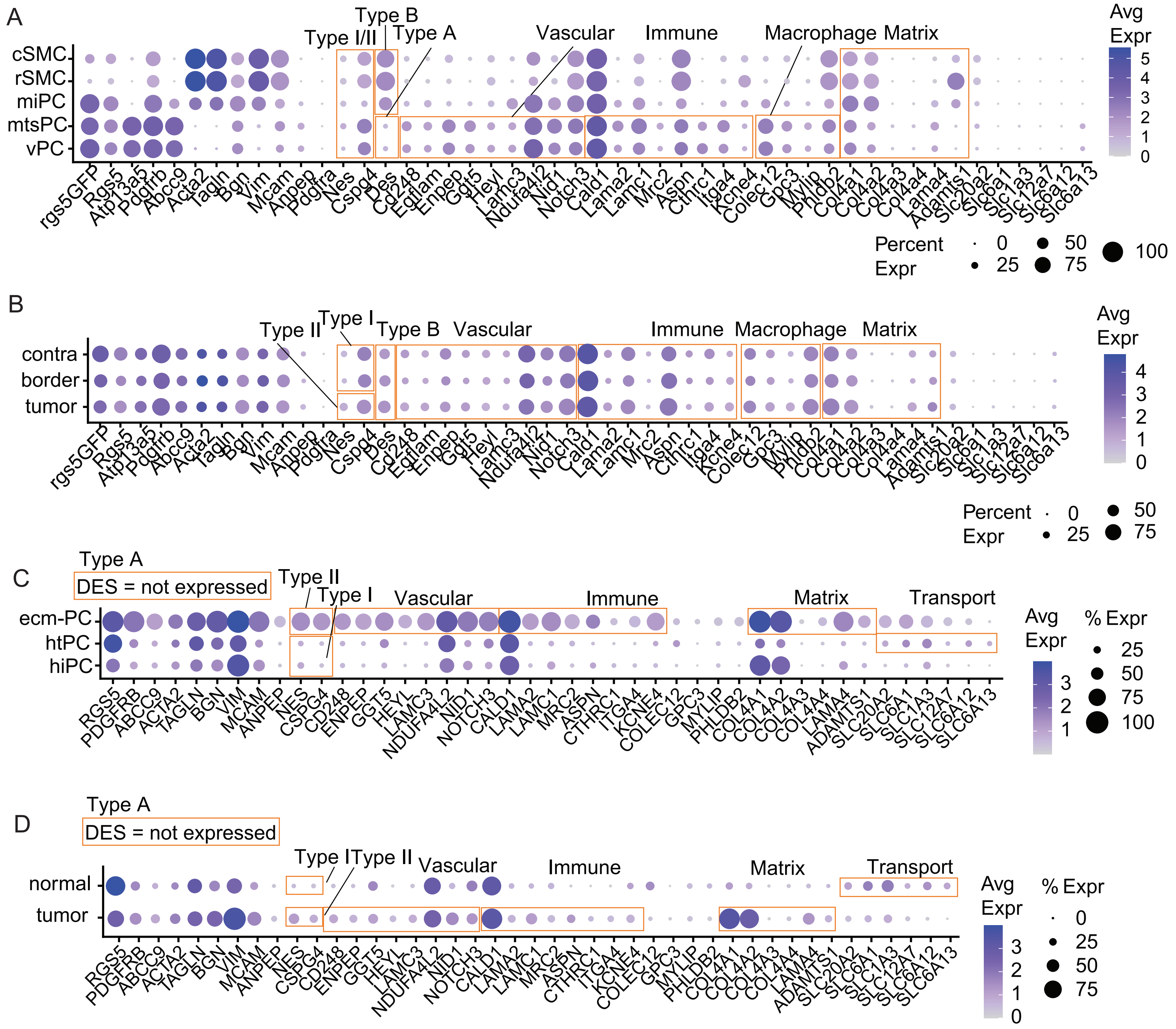

Type A Desmin-

Type B Desmin+, Acta2+

Type I Nestin-, Cspg4+

Type II Nestin+, Cspg4+

Matrix Col4a1-Col4a4+, Lama4+, Adamts1+

Transport Slc20a2+, Slc6a1+, Slc1a3+, Slc12a7+, Slc6a12+, Slc6a13+

Vascular Cd248+, Egflam+, Enpep+, Ggt5+, Heyl+, Lamc3+, Ndufa4l2+, Nid1+, Notch3+

Immune Cald1+, Lama2+, Lamc1+, Mrc2+, Aspn+, Cthrc1+, Itga4+, Kcne4+

Macrophages Colec12+, Gpc3+, Mylip+, Phldb2+
