# Supplementary material for: Pericytes change function depending on glioblastoma vicinity: emphasis on immune regulation
Source: Mol Oncol. 2025 Jul 17;19(9):2491–514. doi: 10.1002/1878-0261.70095 (PMC12420362; doi:10.1002/1878-0261.70095)
Supplement: Supplementary file 9 — Table S1. Mouse sequencing data and cell numbers. [file MOL2-19-2491-s005.docx]

Supplementary Table 1

**A. Mouse sequencing data and cell numbers.**

|  | Mouse 1 | | | Mouse 2 | | | Mouse 3 | | | |
| --- | --- | --- | --- | --- | --- | --- | --- | --- | --- | --- |
|  | **Contra** | **Border** | **Tumor** | **Contra** | **Border** | **Tumor** | **Contra** | **Border** | **Tumor** |  |
| Estimated Number of Cells | 5910 | 7878 | 2662 | 4105 | 15080 | 6802 | 3953 | 7882 | 4637 |  |
| Mean Reads per  Cell | 28430 | 24870 | 83051 | 51525 | 13207 | 32537 | 32169 | 28512 | 40150 |  |
| Median Genes per  Cell | 1558 | 1331 | 1911 | 1657 | 1172 | 1808 | 1762 | 1489 | 1720 |  |
| Number of Cells after Processing | 4799 | 5637 | 3468 | 3070 | 12485 | 5858 | 3376 | 6614 | 3985 |  |
| Number of Mural Cells | 228 | 483 | 41 | 139 | 621 | 135 | 93 | 513 | 39 |  |

Summary of the output data from Cellranger for the mouse dataset, including the estimated number of cells, mean reads per cell, median genes per cell, and the fraction of reads in cells. The table also presents the number of cells retained for analysis after applying CellBender, DoubletFinder, and filtering steps, as well as the number of mural cells identified at the chosen clustering resolution.

**B. Human cell numbers.**

|  | Patient 1 | | Patient 2 | | Patient 3 | | | Patient 4 | |
| --- | --- | --- | --- | --- | --- | --- | --- | --- | --- |
|  | **Non-malignant** | **Tumor** | **Non-malignant** | **Tumor** | | **Non-malignant** | **Tumor** | **Non-malignant** | **Tumor** |
| Number of Cells after Processing | 8673 | 17814 | 9814 | 13038 | | 12239 | 11915 | 14556 | 12948 |
| Number of Mural Cells | 117 | 646 | 2 | 43 | | 79 | 15 | 66 | 4 |

Number of cells retained for analysis after applying CellBender, DoubletFinder, and filtering steps to the Xie et al. [28] dataset, as well as the number of mural cells identified at the chosen clustering resolution.
