# Supplementary material for: Pericytes change function depending on glioblastoma vicinity: emphasis on immune regulation
Source: Mol Oncol. 2025 Jul 17;19(9):2491–514. doi: 10.1002/1878-0261.70095 (PMC12420362; doi:10.1002/1878-0261.70095)
Supplement: Supplementary file 10 — Table S2. Table showing the top 15 upregulated DEGs that characterize each cell population identified in the mouse dataset. [file MOL2-19-2491-s003.docx]

Supplementary Table 2

Table showing the top 15 upregulated DEGs that characterize each cell population identified in the mouse dataset.

| gene | avg_log2FC | p_val_adj | cluster |
| --- | --- | --- | --- |
| Flt1 | 5,376 | 0 | 0_EC |
| Slco1a4 | 4,953 | 0 | 0_EC |
| Spock2 | 4,690 | 0 | 0_EC |
| Cldn5 | 4,393 | 0 | 0_EC |
| Itm2a | 4,322 | 0 | 0_EC |
| Ly6c1 | 4,256 | 0 | 0_EC |
| Pltp | 4,173 | 0 | 0_EC |
| Ramp2 | 4,153 | 0 | 0_EC |
| Slco1c1 | 4,146 | 0 | 0_EC |
| Ptprb | 4,128 | 0 | 0_EC |
| Car4 | 4,057 | 0 | 0_EC |
| Abcg2 | 4,004 | 0 | 0_EC |
| Abcb1a | 3,972 | 0 | 0_EC |
| Adgrl4 | 3,967 | 0 | 0_EC |
| Slc2a1 | 3,958 | 0 | 0_EC |
| Cd83 | 3,563 | 0 | 1_Microglia |
| Ccl3 | 3,539 | 0 | 1_Microglia |
| Ccl4 | 3,455 | 0 | 1_Microglia |
| H2-Aa | 3,442 | 0 | 1_Microglia |
| H2-Eb1 | 3,423 | 0 | 1_Microglia |
| H2-Ab1 | 3,288 | 0 | 1_Microglia |
| Il1a | 3,113 | 0 | 1_Microglia |
| Cd74 | 3,039 | 0 | 1_Microglia |
| Cx3cr1 | 2,903 | 0 | 1_Microglia |
| Atf3 | 2,812 | 0 | 1_Microglia |
| Hexb | 2,777 | 0 | 1_Microglia |
| C1qa | 2,739 | 0 | 1_Microglia |
| C1qc | 2,730 | 0 | 1_Microglia |
| C1qb | 2,684 | 0 | 1_Microglia |
| Trem2 | 2,682 | 0 | 1_Microglia |
| Mobp | 9,241 | 0 | 2_OligoDC |
| Edil3 | 8,072 | 0 | 2_OligoDC |
| Ermn | 7,952 | 0 | 2_OligoDC |
| Mapt | 7,904 | 0 | 2_OligoDC |
| Ugt8a | 7,842 | 0 | 2_OligoDC |
| Mog | 7,822 | 0 | 2_OligoDC |
| Gpr37 | 7,666 | 0 | 2_OligoDC |
| Aspa | 7,650 | 0 | 2_OligoDC |
| Cldn11 | 7,616 | 0 | 2_OligoDC |
| Tspan2 | 7,610 | 0 | 2_OligoDC |
| Mag | 7,595 | 0 | 2_OligoDC |
| Ptprd | 7,567 | 0 | 2_OligoDC |
| Aplp1 | 7,446 | 0 | 2_OligoDC |
| Stmn4 | 7,441 | 0 | 2_OligoDC |
| Mbp | 7,429 | 0 | 2_OligoDC |
| P2ry12 | 1,553 | 0 | 3_Microglia |
| Gpr34 | 1,531 | 0 | 3_Microglia |
| Cx3cr1 | 1,484 | 0 | 3_Microglia |
| Egr1 | 2,545 | 0 | 3_Microglia |
| Siglech | 1,485 | 0 | 3_Microglia |
| Fcrls | 1,756 | 0 | 3_Microglia |
| Fosb | 2,637 | 0 | 3_Microglia |
| Tmem119 | 1,543 | 0 | 3_Microglia |
| Hpgd | 1,936 | 0 | 3_Microglia |
| Mylip | 2,240 | 0 | 3_Microglia |
| Trem2 | 1,221 | 0 | 3_Microglia |
| Cd83 | 2,033 | 0 | 3_Microglia |
| Wsb1 | 2,464 | 0 | 3_Microglia |
| Lpcat2 | 1,275 | 0 | 3_Microglia |
| Olfml3 | 1,386 | 0 | 3_Microglia |
| Gpr34 | 2,481 | 0 | 4_Microglia |
| P2ry12 | 2,282 | 0 | 4_Microglia |
| Siglech | 2,166 | 0 | 4_Microglia |
| Cx3cr1 | 2,163 | 0 | 4_Microglia |
| Tmem119 | 2,144 | 0 | 4_Microglia |
| Olfml3 | 2,132 | 0 | 4_Microglia |
| Fcrls | 2,112 | 0 | 4_Microglia |
| Trem2 | 2,052 | 0 | 4_Microglia |
| Lpcat2 | 2,047 | 0 | 4_Microglia |
| Csf1r | 2,022 | 0 | 4_Microglia |
| Hexb | 2,017 | 0 | 4_Microglia |
| Vsir | 2,010 | 0 | 4_Microglia |
| C1qc | 2,010 | 0 | 4_Microglia |
| C1qa | 2,003 | 0 | 4_Microglia |
| Selplg | 1,999 | 0 | 4_Microglia |
| Gpr34 | 2,868 | 0 | 5_Microglia |
| Fcrls | 2,808 | 0 | 5_Microglia |
| P2ry12 | 2,730 | 0 | 5_Microglia |
| Siglech | 2,693 | 0 | 5_Microglia |
| Cx3cr1 | 2,662 | 0 | 5_Microglia |
| Tmem119 | 2,654 | 0 | 5_Microglia |
| Olfml3 | 2,622 | 0 | 5_Microglia |
| Tgfbr1 | 2,596 | 0 | 5_Microglia |
| Vsir | 2,588 | 0 | 5_Microglia |
| Maf | 2,505 | 0 | 5_Microglia |
| Csf1r | 2,499 | 0 | 5_Microglia |
| Trem2 | 2,443 | 0 | 5_Microglia |
| Lpcat2 | 2,434 | 0 | 5_Microglia |
| Hpgd | 2,424 | 0 | 5_Microglia |
| Hpgds | 2,387 | 0 | 5_Microglia |
| Tm4sf1 | 8,415 | 0 | 6_EC |
| Flt1 | 7,456 | 0 | 6_EC |
| Ptprb | 6,840 | 0 | 6_EC |
| Cldn5 | 6,275 | 0 | 6_EC |
| Adgrf5 | 6,134 | 0 | 6_EC |
| Ly6c1 | 4,785 | 0 | 6_EC |
| Cxcl12 | 4,746 | 0 | 6_EC |
| Spock2 | 4,543 | 0 | 6_EC |
| Timp3 | 4,351 | 0 | 6_EC |
| Adgrl4 | 4,070 | 0 | 6_EC |
| S1pr1 | 3,704 | 0 | 6_EC |
| Itm2a | 3,692 | 0 | 6_EC |
| Sgms1 | 3,667 | 0 | 6_EC |
| Pltp | 3,662 | 0 | 6_EC |
| Slco1c1 | 3,640 | 0 | 6_EC |
| Ttr | 5,857 | 0 | 7_Tumor |
| Hbb-bs | 5,838 | 0 | 7_Tumor |
| Plp1 | 5,806 | 0 | 7_Tumor |
| Ptgds | 5,718 | 0 | 7_Tumor |
| Cryab | 5,607 | 0 | 7_Tumor |
| Spp1 | 5,445 | 0 | 7_Tumor |
| Mcherry | 4,973 | 0 | 7_Tumor |
| Cnp | 4,921 | 0 | 7_Tumor |
| Hba-a1 | 4,878 | 0 | 7_Tumor |
| Car2 | 4,785 | 0 | 7_Tumor |
| Apod | 4,696 | 0 | 7_Tumor |
| AI506816 | 4,590 | 0 | 7_Tumor |
| Hbb-bt | 4,537 | 0 | 7_Tumor |
| Cxcl10 | 4,379 | 0 | 7_Tumor |
| Mbp | 3,636 | 0 | 7_Tumor |
| H2-Aa | 2,741 | 0 | 8_Microglia |
| H2-Eb1 | 2,307 | 0 | 8_Microglia |
| Cd74 | 2,307 | 7.39565464247867e-290 | 8_Microglia |
| H2-Ab1 | 2,219 | 0 | 8_Microglia |
| C1qc | 2,178 | 0 | 8_Microglia |
| C1qa | 2,158 | 0 | 8_Microglia |
| C1qb | 2,145 | 0 | 8_Microglia |
| Hexb | 2,136 | 0 | 8_Microglia |
| Trem2 | 2,124 | 0 | 8_Microglia |
| Ly86 | 2,098 | 0 | 8_Microglia |
| Fcer1g | 2,068 | 0 | 8_Microglia |
| Ctss | 2,047 | 1.53409025716985e-256 | 8_Microglia |
| Tyrobp | 2,042 | 0 | 8_Microglia |
| Lgmn | 2,031 | 0 | 8_Microglia |
| Aif1 | 2,009 | 0 | 8_Microglia |
| Cd3g | 8,428 | 0 | 9_T_cells |
| Trbc2 | 7,921 | 0 | 9_T_cells |
| Cd3d | 7,815 | 0 | 9_T_cells |
| Trac | 7,570 | 0 | 9_T_cells |
| Cd3e | 7,399 | 0 | 9_T_cells |
| Icos | 7,385 | 0 | 9_T_cells |
| Cd2 | 6,663 | 0 | 9_T_cells |
| S100a10 | 6,410 | 0 | 9_T_cells |
| Ctla4 | 6,020 | 0 | 9_T_cells |
| Tnfrsf4 | 5,921 | 0 | 9_T_cells |
| Cd28 | 5,888 | 0 | 9_T_cells |
| Cytip | 5,861 | 0 | 9_T_cells |
| Ptpn22 | 5,807 | 0 | 9_T_cells |
| Rac2 | 5,796 | 0 | 9_T_cells |
| Ltb | 5,746 | 0 | 9_T_cells |
| Tgfbi | 8,316 | 0 | 10_Macrophages |
| Ly6i | 7,613 | 0 | 10_Macrophages |
| Ms4a6c | 7,066 | 0 | 10_Macrophages |
| Cybb | 6,966 | 0 | 10_Macrophages |
| Lgals3 | 6,914 | 0 | 10_Macrophages |
| Cfb | 6,721 | 0 | 10_Macrophages |
| C3 | 6,315 | 0 | 10_Macrophages |
| Ccl5 | 6,236 | 0 | 10_Macrophages |
| Cxcl16 | 6,178 | 0 | 10_Macrophages |
| Ms4a4c | 6,016 | 0 | 10_Macrophages |
| Cxcr4 | 5,909 | 0 | 10_Macrophages |
| Il1rn | 5,886 | 0 | 10_Macrophages |
| Pla2g7 | 5,794 | 0 | 10_Macrophages |
| AB124611 | 5,723 | 0 | 10_Macrophages |
| Il1b | 5,709 | 0 | 10_Macrophages |
| Spock2 | 4,083 | 0 | 11_EC |
| Flt1 | 3,526 | 0 | 11_EC |
| Cldn5 | 3,278 | 0 | 11_EC |
| Itm2a | 3,158 | 0 | 11_EC |
| Ly6c1 | 2,763 | 0 | 11_EC |
| Slco1a4 | 2,711 | 0 | 11_EC |
| Jcad | 2,675 | 0 | 11_EC |
| Pltp | 2,548 | 8.62261926407745e-285 | 11_EC |
| Adgrl4 | 2,533 | 0 | 11_EC |
| Id1 | 2,485 | 0 | 11_EC |
| Abcb1a | 2,409 | 0 | 11_EC |
| Ptprb | 2,388 | 0 | 11_EC |
| Adgrf5 | 2,374 | 0 | 11_EC |
| Sgms1 | 2,341 | 8.21592901040785e-217 | 11_EC |
| Abcg2 | 2,336 | 9.22156847382884e-299 | 11_EC |
| Vtn | 9,790 | 0 | 12_PC |
| Pdgfrb | 9,653 | 0 | 12_PC |
| Atp1a2 | 9,576 | 0 | 12_PC |
| rgs5GFP | 9,286 | 0 | 12_PC |
| Ndufa4l2 | 9,222 | 0 | 12_PC |
| Cald1 | 8,850 | 0 | 12_PC |
| Myl9 | 8,774 | 0 | 12_PC |
| Rgs4 | 8,658 | 0 | 12_PC |
| Gucy1b1 | 8,499 | 0 | 12_PC |
| Higd1b | 8,454 | 0 | 12_PC |
| Abcc9 | 8,424 | 0 | 12_PC |
| Atp13a5 | 8,412 | 0 | 12_PC |
| Kcnj8 | 8,237 | 0 | 12_PC |
| P2ry14 | 8,160 | 0 | 12_PC |
| Mfge8 | 8,131 | 0 | 12_PC |
| Nkg7 | 8,196 | 0 | 13_T_cells |
| Cd3g | 8,052 | 0 | 13_T_cells |
| Ms4a4b | 7,485 | 0 | 13_T_cells |
| Cd3d | 6,275 | 0 | 13_T_cells |
| Trbc2 | 6,001 | 0 | 13_T_cells |
| Cd3e | 5,989 | 0 | 13_T_cells |
| Cd8b1 | 5,786 | 0 | 13_T_cells |
| Trac | 5,586 | 0 | 13_T_cells |
| Ccl5 | 5,247 | 0 | 13_T_cells |
| Cd8a | 5,220 | 0 | 13_T_cells |
| S100a10 | 5,141 | 0 | 13_T_cells |
| Ptpn22 | 5,112 | 0 | 13_T_cells |
| Klrd1 | 5,088 | 0 | 13_T_cells |
| Ctsw | 5,036 | 0 | 13_T_cells |
| Cd2 | 4,978 | 0 | 13_T_cells |
| Ms4a6c | 9,248 | 0 | 14_Macrophages |
| Mrc1 | 8,662 | 0 | 14_Macrophages |
| Ms4a7 | 8,319 | 0 | 14_Macrophages |
| Cybb | 8,042 | 0 | 14_Macrophages |
| Lyz2 | 7,801 | 0 | 14_Macrophages |
| Pf4 | 7,109 | 0 | 14_Macrophages |
| Ifi207 | 6,775 | 0 | 14_Macrophages |
| Dab2 | 6,762 | 0 | 14_Macrophages |
| Ms4a6b | 6,199 | 0 | 14_Macrophages |
| F13a1 | 6,088 | 0 | 14_Macrophages |
| Lst1 | 5,686 | 0 | 14_Macrophages |
| Blvrb | 5,674 | 0 | 14_Macrophages |
| Tmem176b | 5,632 | 0 | 14_Macrophages |
| Fcer1g | 5,516 | 0 | 14_Macrophages |
| Wfdc17 | 5,167 | 0 | 14_Macrophages |
| Myh11 | 10,469 | 0 | 15_PC |
| Tagln | 9,882 | 0 | 15_PC |
| Tpm2 | 8,669 | 0 | 15_PC |
| Acta2 | 9,773 | 0 | 15_PC |
| Mylk | 7,188 | 0 | 15_PC |
| Myl9 | 6,486 | 0 | 15_PC |
| Mustn1 | 8,790 | 0 | 15_PC |
| Pln | 10,415 | 0 | 15_PC |
| Palld | 8,814 | 0 | 15_PC |
| Sncg | 7,752 | 0 | 15_PC |
| Lmod1 | 9,140 | 0 | 15_PC |
| Aspn | 4,668 | 0 | 15_PC |
| Fxyd1 | 5,170 | 0 | 15_PC |
| Cald1 | 3,878 | 0 | 15_PC |
| Pde3a | 6,930 | 0 | 15_PC |
| Slc1a2 | 10,687 | 0 | 16_Astrocytes |
| Ptprz1 | 9,015 | 0 | 16_Astrocytes |
| Atp1a2 | 3,992 | 0 | 16_Astrocytes |
| Nrxn1 | 8,366 | 0 | 16_Astrocytes |
| Ttyh1 | 6,929 | 0 | 16_Astrocytes |
| Dclk1 | 8,107 | 0 | 16_Astrocytes |
| Ntrk2 | 7,882 | 0 | 16_Astrocytes |
| Gpm6a | 7,860 | 0 | 16_Astrocytes |
| Slc4a4 | 10,703 | 0 | 16_Astrocytes |
| Gja1 | 6,539 | 0 | 16_Astrocytes |
| F3 | 7,636 | 0 | 16_Astrocytes |
| Plpp3 | 5,919 | 0 | 16_Astrocytes |
| Lsamp | 6,541 | 0 | 16_Astrocytes |
| Bcan | 8,771 | 0 | 16_Astrocytes |
| Cspg5 | 9,236 | 0 | 16_Astrocytes |
| Lsp1 | 4,047 | 0 | 17_DC |
| Cytip | 3,737 | 0 | 17_DC |
| Traf1 | 3,955 | 0 | 17_DC |
| Napsa | 5,529 | 0 | 17_DC |
| Plbd1 | 5,231 | 0 | 17_DC |
| Pim1 | 2,562 | 0 | 17_DC |
| Slamf7 | 3,646 | 0 | 17_DC |
| S100a11 | 2,154 | 0 | 17_DC |
| Pkib | 3,987 | 0 | 17_DC |
| Crip1 | 2,449 | 0 | 17_DC |
| Ifi30 | 3,180 | 0 | 17_DC |
| H2-Aa | 2,664 | 0 | 17_DC |
| Lmnb1 | 3,126 | 0 | 17_DC |
| Cxcl16 | 2,675 | 0 | 17_DC |
| Syngr2 | 2,860 | 0 | 17_DC |
| Igkc | 13,977 | 0 | 18_B_cells |
| Cd79a | 11,477 | 0 | 18_B_cells |
| Ly6d | 7,484 | 0 | 18_B_cells |
| Plac8 | 2,799 | 0 | 18_B_cells |
| Mzb1 | 10,373 | 0 | 18_B_cells |
| H2-DMb2 | 5,423 | 0 | 18_B_cells |
| Iglc2 | 11,539 | 0 | 18_B_cells |
| Cd79b | 4,879 | 0 | 18_B_cells |
| Ms4a1 | 13,393 | 0 | 18_B_cells |
| Napsa | 4,304 | 0 | 18_B_cells |
| Iglc3 | 8,233 | 0 | 18_B_cells |
| H2-Ob | 3,740 | 0 | 18_B_cells |
| BE692007 | 4,594 | 0 | 18_B_cells |
| Top2a | 4,345 | 0 | 18_B_cells |
| Pclaf | 4,701 | 0 | 18_B_cells |
| Sox11 | 9,921 | 0 | 19_Neural_progenitors |
| Rtn1 | 6,137 | 0 | 19_Neural_progenitors |
| Stmn3 | 7,979 | 0 | 19_Neural_progenitors |
| Map1b | 5,367 | 0 | 19_Neural_progenitors |
| Nnat | 7,072 | 0 | 19_Neural_progenitors |
| Tubb2b | 6,514 | 0 | 19_Neural_progenitors |
| Ncam1 | 4,422 | 0 | 19_Neural_progenitors |
| Meis2 | 7,335 | 0 | 19_Neural_progenitors |
| Stmn2 | 6,257 | 0 | 19_Neural_progenitors |
| Tubb3 | 6,691 | 0 | 19_Neural_progenitors |
| Pbx1 | 4,702 | 0 | 19_Neural_progenitors |
| Map2 | 5,789 | 0 | 19_Neural_progenitors |
| Dcx | 11,609 | 0 | 19_Neural_progenitors |
| Bcl11a | 8,036 | 0 | 19_Neural_progenitors |
| Sox4 | 4,455 | 0 | 19_Neural_progenitors |
| Nkg7 | 8,881 | 0 | 20_NK_cells |
| Klre1 | 8,782 | 0 | 20_NK_cells |
| Il2rb | 8,232 | 0 | 20_NK_cells |
| Ms4a4b | 7,950 | 0 | 20_NK_cells |
| Gzma | 7,868 | 0 | 20_NK_cells |
| Ccl5 | 7,469 | 0 | 20_NK_cells |
| Klrd1 | 7,433 | 0 | 20_NK_cells |
| Klrk1 | 7,291 | 0 | 20_NK_cells |
| Vps37b | 6,901 | 0 | 20_NK_cells |
| Ctsw | 6,768 | 0 | 20_NK_cells |
| Xcl1 | 6,714 | 0 | 20_NK_cells |
| Txk | 6,512 | 0 | 20_NK_cells |
| Ptpn22 | 6,330 | 0 | 20_NK_cells |
| AW112010 | 5,966 | 0 | 20_NK_cells |
| Ugcg | 5,965 | 0 | 20_NK_cells |
| Mef2a | 4,004 | 2.79521573550904e-91 | 21_Microglia |
| Srgap2 | 3,893 | 1.35331780449452e-67 | 21_Microglia |
| Mef2c | 3,884 | 1.53908959136358e-48 | 21_Microglia |
| Mafb | 3,858 | 2.10987881414624e-70 | 21_Microglia |
| Dleu2 | 3,821 | 2.89988758422164e-121 | 21_Microglia |
| Jmjd1c | 3,789 | 2.77846254816e-50 | 21_Microglia |
| Kctd12 | 3,788 | 5.03553668435946e-126 | 21_Microglia |
| Wsb1 | 3,753 | 5.47016938539766e-93 | 21_Microglia |
| Fus | 3,748 | 3.30691448008964e-50 | 21_Microglia |
| Ssh2 | 3,638 | 9.94361758243896e-62 | 21_Microglia |
| Hpgds | 3,552 | 1.08533220999625e-110 | 21_Microglia |
| Tgfbr1 | 3,545 | 1.56081958572693e-74 | 21_Microglia |
| Zfhx3 | 3,542 | 6.345706017444e-93 | 21_Microglia |
| Nav3 | 3,495 | 1.94903119947792e-62 | 21_Microglia |
| Dennd4a | 3,393 | 2.89388031593874e-87 | 21_Microglia |
| Ecrg4 | 9,915 | 0 | 22_Choroid_plexus |
| Chchd10 | 9,913 | 0 | 22_Choroid_plexus |
| Ttr | 9,784 | 0 | 22_Choroid_plexus |
| Calml4 | 9,700 | 0 | 22_Choroid_plexus |
| Clu | 9,692 | 0 | 22_Choroid_plexus |
| Rbp1 | 9,668 | 0 | 22_Choroid_plexus |
| Mt3 | 9,500 | 0 | 22_Choroid_plexus |
| Kl | 9,487 | 0 | 22_Choroid_plexus |
| Kcnj13 | 9,423 | 0 | 22_Choroid_plexus |
| Enpp2 | 9,404 | 0 | 22_Choroid_plexus |
| Fxyd1 | 9,331 | 0 | 22_Choroid_plexus |
| Folr1 | 9,311 | 0 | 22_Choroid_plexus |
| Igfbp2 | 9,127 | 0 | 22_Choroid_plexus |
| 2900040C04Rik | 9,123 | 0 | 22_Choroid_plexus |
| Atp1b1 | 8,970 | 0 | 22_Choroid_plexus |
| Plvap | 10,964 | 0 | 23_EC |
| Plpp1 | 6,413 | 0 | 23_EC |
| Igfbp3 | 8,732 | 0 | 23_EC |
| Plpp3 | 5,303 | 0 | 23_EC |
| Timp3 | 3,664 | 0 | 23_EC |
| Emcn | 3,701 | 0 | 23_EC |
| Kdr | 3,662 | 0 | 23_EC |
| Tcim | 4,897 | 0 | 23_EC |
| Clec14a | 4,457 | 0 | 23_EC |
| Plscr2 | 5,697 | 0 | 23_EC |
| Prss23 | 3,839 | 0 | 23_EC |
| Hspg2 | 4,514 | 0 | 23_EC |
| Cd24a | 6,277 | 0 | 23_EC |
| Fam167b | 4,790 | 0 | 23_EC |
| Col13a1 | 9,284 | 0 | 23_EC |
| Mki67 | 7,041 | 0 | 24_T_cells |
| Birc5 | 7,039 | 0 | 24_T_cells |
| Pclaf | 7,036 | 0 | 24_T_cells |
| Cd3g | 6,939 | 0 | 24_T_cells |
| Top2a | 6,908 | 0 | 24_T_cells |
| Cd3e | 6,836 | 0 | 24_T_cells |
| Trac | 6,792 | 0 | 24_T_cells |
| Cd3d | 6,728 | 0 | 24_T_cells |
| Cdca8 | 6,693 | 0 | 24_T_cells |
| Trbc2 | 6,588 | 0 | 24_T_cells |
| Dut | 6,536 | 0 | 24_T_cells |
| Cd2 | 6,531 | 0 | 24_T_cells |
| Cks1b | 6,459 | 0 | 24_T_cells |
| Ptprcap | 6,454 | 0 | 24_T_cells |
| Smc2 | 6,446 | 0 | 24_T_cells |
| AI506816 | 8,882 | 0 | 25_Tumor |
| Gpnmb | 8,633 | 0 | 25_Tumor |
| Col11a1 | 8,165 | 0 | 25_Tumor |
| H19 | 7,830 | 0 | 25_Tumor |
| Moxd1 | 7,622 | 0 | 25_Tumor |
| Col9a2 | 7,595 | 0 | 25_Tumor |
| Cdkn2a | 7,226 | 0 | 25_Tumor |
| Chl1 | 7,217 | 0 | 25_Tumor |
| Sox6 | 6,985 | 0 | 25_Tumor |
| Pet2 | 6,964 | 0 | 25_Tumor |
| Gm46620 | 6,949 | 0 | 25_Tumor |
| Rorb | 6,857 | 0 | 25_Tumor |
| Neto1 | 6,666 | 0 | 25_Tumor |
| S100b | 6,639 | 0 | 25_Tumor |
| Apol9a | 6,595 | 0 | 25_Tumor |
| S100a9 | 9,253 | 0 | 26_Neutrophils |
| S100a8 | 8,997 | 0 | 26_Neutrophils |
| Slpi | 8,921 | 0 | 26_Neutrophils |
| Hdc | 8,893 | 0 | 26_Neutrophils |
| Hcar2 | 8,786 | 0 | 26_Neutrophils |
| Clec4d | 8,773 | 0 | 26_Neutrophils |
| Cxcr2 | 8,416 | 0 | 26_Neutrophils |
| Slfn4 | 8,355 | 0 | 26_Neutrophils |
| Il1r2 | 8,278 | 0 | 26_Neutrophils |
| Retnlg | 7,999 | 0 | 26_Neutrophils |
| Ccr1 | 7,978 | 0 | 26_Neutrophils |
| Arg2 | 7,924 | 0 | 26_Neutrophils |
| Slfn1 | 7,835 | 0 | 26_Neutrophils |
| G0s2 | 7,614 | 1.74457148155207e-253 | 26_Neutrophils |
| Trim30b | 7,599 | 0 | 26_Neutrophils |
| Dcn | 9,930 | 0 | 27_Fibroblasts |
| Col1a1 | 9,912 | 0 | 27_Fibroblasts |
| Col1a2 | 9,811 | 0 | 27_Fibroblasts |
| Lum | 9,682 | 0 | 27_Fibroblasts |
| Serping1 | 9,313 | 0 | 27_Fibroblasts |
| Efemp1 | 9,159 | 0 | 27_Fibroblasts |
| Bgn | 8,847 | 0 | 27_Fibroblasts |
| Colec12 | 8,834 | 0 | 27_Fibroblasts |
| Nr2f2 | 8,662 | 0 | 27_Fibroblasts |
| Pdgfra | 8,608 | 0 | 27_Fibroblasts |
| Mdk | 8,435 | 0 | 27_Fibroblasts |
| F3 | 8,435 | 0 | 27_Fibroblasts |
| Cped1 | 8,191 | 0 | 27_Fibroblasts |
| Pcolce | 8,083 | 0 | 27_Fibroblasts |
| Igfbp4 | 7,878 | 0 | 27_Fibroblasts |
| Ly6d | 9,864 | 0 | 28_DC |
| Ccr9 | 9,675 | 0 | 28_DC |
| Gm21762 | 9,538 | 0 | 28_DC |
| Cox6a2 | 8,935 | 0 | 28_DC |
| Iglc3 | 8,433 | 0 | 28_DC |
| Sell | 8,179 | 0 | 28_DC |
| Bcl11a | 8,021 | 0 | 28_DC |
| Smim5 | 7,998 | 0 | 28_DC |
| Runx2 | 7,598 | 0 | 28_DC |
| Cd300c | 7,452 | 0 | 28_DC |
| Pacsin1 | 7,208 | 0 | 28_DC |
| Upb1 | 6,865 | 0 | 28_DC |
| Spib | 6,839 | 0 | 28_DC |
| Cd7 | 6,688 | 0 | 28_DC |
| Cdh1 | 6,539 | 0 | 28_DC |
