# Supplementary material for: Pericytes change function depending on glioblastoma vicinity: emphasis on immune regulation
Source: Mol Oncol. 2025 Jul 17;19(9):2491–514. doi: 10.1002/1878-0261.70095 (PMC12420362; doi:10.1002/1878-0261.70095)
Supplement: Supplementary file 11 — Table S3. Table listing the top 15 upregulated DEGs that characterize each cell population identified in the human dataset. [file MOL2-19-2491-s010.docx]

Supplementary Table 3

Table listing the top 15 upregulated DEGs that characterize each cell population identified in the human dataset.

| gene | avg_log2FC | p_val_adj | cluster |
| --- | --- | --- | --- |
| TCHH | 2,483 | 0 | 0_Macrophages |
| MIR155HG | 2,164 | 0 | 0_Macrophages |
| IL6 | 1,943 | 0 | 0_Macrophages |
| RCAN1 | 1,837 | 0 | 0_Macrophages |
| IL1B | 1,789 | 0 | 0_Macrophages |
| ADAMDEC1 | 1,780 | 0 | 0_Macrophages |
| ARL4C | 1,767 | 0 | 0_Macrophages |
| DUSP2 | 1,759 | 0 | 0_Macrophages |
| BIRC3 | 1,653 | 0 | 0_Macrophages |
| RUNX3 | 1,632 | 0 | 0_Macrophages |
| CYB5D1 | 1,617 | 0 | 0_Macrophages |
| PRDM1 | 1,600 | 0 | 0_Macrophages |
| CADM1 | 1,597 | 0 | 0_Macrophages |
| THAP2 | 1,590 | 0 | 0_Macrophages |
| ITGA4 | 1,527 | 0 | 0_Macrophages |
| ACY3 | 2,343 | 0 | 1_Microglia |
| AP005530.1 | 2,227 | 0 | 1_Microglia |
| CH25H | 2,214 | 0 | 1_Microglia |
| SPRY1 | 2,085 | 0 | 1_Microglia |
| RASGEF1C | 2,081 | 0 | 1_Microglia |
| PDK4 | 2,047 | 0 | 1_Microglia |
| LYVE1 | 2,027 | 0 | 1_Microglia |
| CECR2 | 2,007 | 0 | 1_Microglia |
| TNFRSF13C | 2,004 | 0 | 1_Microglia |
| P2RY12 | 1,947 | 0 | 1_Microglia |
| SLC15A2 | 1,892 | 0 | 1_Microglia |
| TAL1 | 1,876 | 0 | 1_Microglia |
| TMIGD2 | 1,853 | 0 | 1_Microglia |
| PDGFB | 1,782 | 0 | 1_Microglia |
| ADAMTSL2 | 1,727 | 0 | 1_Microglia |
| PTCH2 | 2,164 | 0 | 2_Microglia |
| AC084871.2 | 2,143 | 0 | 2_Microglia |
| FP700111.1 | 2,008 | 0 | 2_Microglia |
| AC245014.3 | 1,990 | 0 | 2_Microglia |
| AC007952.4 | 1,856 | 0 | 2_Microglia |
| Z93241.1 | 1,846 | 0 | 2_Microglia |
| LILRA4 | 1,802 | 0 | 2_Microglia |
| TMEM107 | 1,797 | 0 | 2_Microglia |
| AL360012.1 | 1,795 | 0 | 2_Microglia |
| IFIT2 | 1,649 | 0 | 2_Microglia |
| C12orf57 | 1,638 | 0 | 2_Microglia |
| WDR74 | 1,625 | 0 | 2_Microglia |
| SERPINE1 | 1,624 | 0 | 2_Microglia |
| PLA2G7 | 1,586 | 0 | 2_Microglia |
| AC103591.3 | 1,552 | 0 | 2_Microglia |
| EIF4A2 | 0,376 | 2.51276088236061e-21 | 3_Microglia |
| RBMX | 0,363 | 1.38911759252751e-88 | 3_Microglia |
| YME1L1 | 0,355 | 3.05378946943133e-81 | 3_Microglia |
| EIF5 | 0,345 | 2.88885681433766e-19 | 3_Microglia |
| CCDC88A | 0,335 | 4.37164259516979e-32 | 3_Microglia |
| SRSF11 | 0,329 | 1.0689752497851e-77 | 3_Microglia |
| AKAP9 | 0,317 | 5.40889210052332e-80 | 3_Microglia |
| GNB4 | 0,317 | 1.56673625554145e-57 | 3_Microglia |
| NCKAP1L | 0,311 | 3.8205772679007e-85 | 3_Microglia |
| DDX46 | 0,311 | 3.89600559350791e-109 | 3_Microglia |
| C3AR1 | 0,310 | 6.17520632024927e-25 | 3_Microglia |
| ZFHX3 | 0,295 | 2.3042228697766e-46 | 3_Microglia |
| LHFPL2 | 0,293 | 1.9488388478542e-47 | 3_Microglia |
| PARVG | 0,275 | 1.53187085695259e-145 | 3_Microglia |
| RRBP1 | 0,270 | 1.22951685252905e-85 | 3_Microglia |
| ALDH1A1 | 2,590 | 0 | 4_Macrophages |
| ACP5 | 2,106 | 0 | 4_Macrophages |
| ADAMDEC1 | 2,017 | 0 | 4_Macrophages |
| PKIB | 1,899 | 0 | 4_Macrophages |
| SELENOP | 1,823 | 0 | 4_Macrophages |
| CADM1 | 1,714 | 0 | 4_Macrophages |
| TSPAN4 | 1,663 | 0 | 4_Macrophages |
| NUPR1 | 1,535 | 0 | 4_Macrophages |
| FCGR2B | 1,478 | 0 | 4_Macrophages |
| GPNMB | 1,461 | 0 | 4_Macrophages |
| LGALS3 | 1,445 | 0 | 4_Macrophages |
| MS4A6A | 1,383 | 0 | 4_Macrophages |
| PMP22 | 1,378 | 0 | 4_Macrophages |
| SLC40A1 | 1,349 | 0 | 4_Macrophages |
| AZI2 | 1,309 | 0 | 4_Macrophages |
| HNRNPC | 0,365 | 2.79587684680357e-15 | 5_Macrophages |
| ZEB2 | 0,360 | 5.78734971707913e-16 | 5_Macrophages |
| FUS | 0,347 | 1.92490994696668e-08 | 5_Macrophages |
| NUFIP2 | 0,325 | 9.51133993596916e-65 | 5_Macrophages |
| HNRNPH1 | 0,322 | 1.78729485370396e-54 | 5_Macrophages |
| JMJD1C | 0,307 | 5.99055323905101e-61 | 5_Macrophages |
| GOLGA4 | 0,265 | 8.57396366580257e-71 | 5_Macrophages |
| SLC38A2 | 0,250 | 4.29115365530149e-61 | 5_Macrophages |
| DDX18 | 0,249 | 2.51334266804731e-134 | 5_Macrophages |
| CTNNB1 | 0,245 | 8.18188597397511e-101 | 5_Macrophages |
| WTAP | 0,245 | 1.73546119221082e-125 | 5_Macrophages |
| UBE2D3 | 0,221 | 4.80533190933826e-35 | 5_Macrophages |
| SLC16A3 | 0,220 | 1.51347717329522e-63 | 5_Macrophages |
| RNF213 | 0,219 | 6.98938447374949e-69 | 5_Macrophages |
| MAP3K2 | 0,217 | 1.0146083830804e-122 | 5_Macrophages |
| BNIP3 | 2,986 | 0 | 6_Macrophages |
| TGFBI | 2,365 | 0 | 6_Macrophages |
| ADAM8 | 2,326 | 0 | 6_Macrophages |
| RNASE1 | 2,303 | 0 | 6_Macrophages |
| NUPR1 | 2,237 | 0 | 6_Macrophages |
| S100A10 | 2,041 | 0 | 6_Macrophages |
| IL7R | 2,035 | 0 | 6_Macrophages |
| SLC16A10 | 2,006 | 0 | 6_Macrophages |
| FABP5 | 1,986 | 0 | 6_Macrophages |
| CLEC5A | 1,935 | 0 | 6_Macrophages |
| LGALS1 | 1,932 | 0 | 6_Macrophages |
| ANXA2 | 1,881 | 0 | 6_Macrophages |
| F13A1 | 1,871 | 0 | 6_Macrophages |
| CSTB | 1,848 | 0 | 6_Macrophages |
| PMP22 | 1,789 | 0 | 6_Macrophages |
| HSPA1B | 3,290 | 0 | 7_Microglia |
| HSPH1 | 3,249 | 0 | 7_Microglia |
| HSPA1A | 3,178 | 0 | 7_Microglia |
| HSPA6 | 3,096 | 0 | 7_Microglia |
| BAG3 | 2,940 | 0 | 7_Microglia |
| DNAJB1 | 2,852 | 0 | 7_Microglia |
| ZFAND2A | 2,581 | 0 | 7_Microglia |
| HSPB1 | 2,394 | 0 | 7_Microglia |
| HSPD1 | 2,328 | 0 | 7_Microglia |
| HSPE1 | 2,127 | 0 | 7_Microglia |
| DNAJB4 | 2,001 | 0 | 7_Microglia |
| CACNA1A | 1,937 | 0 | 7_Microglia |
| GRID2 | 1,807 | 0 | 7_Microglia |
| CACNB4 | 1,710 | 0 | 7_Microglia |
| CHORDC1 | 1,587 | 0 | 7_Microglia |
| FCGR3B | 7,216 | 0 | 8_Monocytes |
| CMTM2 | 7,026 | 0 | 8_Monocytes |
| S100P | 7,025 | 0 | 8_Monocytes |
| PROK2 | 6,652 | 0 | 8_Monocytes |
| CXCR2 | 6,416 | 0 | 8_Monocytes |
| FPR2 | 6,324 | 0 | 8_Monocytes |
| ADGRG3 | 6,188 | 0 | 8_Monocytes |
| IL18R1 | 6,182 | 0 | 8_Monocytes |
| RIPOR2 | 5,910 | 0 | 8_Monocytes |
| IL1R2 | 5,880 | 0 | 8_Monocytes |
| SMIM25 | 5,647 | 0 | 8_Monocytes |
| IFITM2 | 5,550 | 0 | 8_Monocytes |
| VNN2 | 5,375 | 0 | 8_Monocytes |
| HCAR3 | 5,305 | 0 | 8_Monocytes |
| S100A12 | 5,296 | 0 | 8_Monocytes |
| P2RY12 | 1,948 | 0 | 9_Microglia |
| BIN1 | 1,904 | 0 | 9_Microglia |
| CX3CR1 | 1,650 | 0 | 9_Microglia |
| C12orf75 | 1,973 | 0 | 9_Microglia |
| FCN1 | 5,167 | 0 | 10_Monocytes |
| CXCL5 | 5,069 | 0 | 10_Monocytes |
| SERPINB2 | 4,992 | 0 | 10_Monocytes |
| VCAN | 4,919 | 0 | 10_Monocytes |
| LGALS2 | 4,697 | 0 | 10_Monocytes |
| RETN | 4,629 | 0 | 10_Monocytes |
| EREG | 4,538 | 0 | 10_Monocytes |
| CD300E | 4,387 | 0 | 10_Monocytes |
| TIMP1 | 4,243 | 0 | 10_Monocytes |
| CFP | 4,225 | 0 | 10_Monocytes |
| ANPEP | 4,111 | 0 | 10_Monocytes |
| MARCO | 3,977 | 0 | 10_Monocytes |
| PID1 | 3,941 | 0 | 10_Monocytes |
| CD52 | 3,900 | 0 | 10_Monocytes |
| MGST1 | 3,823 | 0 | 10_Monocytes |
| CLEC14A | 6,909 | 0 | 11_EC |
| KDR | 6,804 | 0 | 11_EC |
| ADGRL4 | 6,780 | 0 | 11_EC |
| MECOM | 6,761 | 0 | 11_EC |
| SRARP | 6,727 | 0 | 11_EC |
| EMCN | 6,705 | 0 | 11_EC |
| CLDN5 | 6,679 | 0 | 11_EC |
| MYCT1 | 6,638 | 0 | 11_EC |
| TM4SF18 | 6,631 | 0 | 11_EC |
| CDH5 | 6,600 | 0 | 11_EC |
| ADGRL2 | 6,593 | 0 | 11_EC |
| CXorf36 | 6,593 | 0 | 11_EC |
| ABCB1 | 6,587 | 0 | 11_EC |
| SOX18 | 6,570 | 0 | 11_EC |
| ITM2A | 6,540 | 0 | 11_EC |
| TOP2A | 6,099 | 0 | 12_Prolif.TAM |
| MKI67 | 6,061 | 0 | 12_Prolif.TAM |
| CDC20 | 6,014 | 0 | 12_Prolif.TAM |
| DLGAP5 | 5,957 | 0 | 12_Prolif.TAM |
| UBE2C | 5,935 | 0 | 12_Prolif.TAM |
| HMMR | 5,920 | 0 | 12_Prolif.TAM |
| ASPM | 5,878 | 0 | 12_Prolif.TAM |
| BIRC5 | 5,824 | 0 | 12_Prolif.TAM |
| NCAPG | 5,692 | 0 | 12_Prolif.TAM |
| MYBL2 | 5,664 | 0 | 12_Prolif.TAM |
| AURKB | 5,612 | 0 | 12_Prolif.TAM |
| RRM2 | 5,579 | 0 | 12_Prolif.TAM |
| ANLN | 5,558 | 0 | 12_Prolif.TAM |
| GTSE1 | 5,557 | 0 | 12_Prolif.TAM |
| CEP55 | 5,540 | 0 | 12_Prolif.TAM |
| GNLY | 2,034 | 0 | 13_Microglia |
| CCL5 | 1,613 | 0 | 13_Microglia |
| C1orf56 | 1,366 | 4.06291176238928e-177 | 13_Microglia |
| BCAS2 | 1,315 | 4.91987408090429e-121 | 13_Microglia |
| CKB | 1,302 | 3.25475840719765e-184 | 13_Microglia |
| PIH1D1 | 1,268 | 1.96337185336539e-161 | 13_Microglia |
| GRASP | 1,234 | 3.70783674523186e-196 | 13_Microglia |
| RPS4Y1 | 1,029 | 1.78233240911876e-131 | 13_Microglia |
| RGS16 | 1,012 | 1.20006846584599e-143 | 13_Microglia |
| LINC01736 | 1,000 | 5.05103273074105e-95 | 13_Microglia |
| PADI2 | 0,988 | 2.16844556600744e-93 | 13_Microglia |
| RHOB | 0,983 | 0 | 13_Microglia |
| NR4A1 | 0,976 | 1.75388968175938e-240 | 13_Microglia |
| CFD | 0,974 | 5.0466872028916e-188 | 13_Microglia |
| JDP2 | 0,966 | 5.1967786130077e-131 | 13_Microglia |
| CXCL10 | 5,335 | 0 | 14_Macrophages |
| IFIT1 | 4,894 | 0 | 14_Macrophages |
| RSAD2 | 4,865 | 0 | 14_Macrophages |
| ISG15 | 4,463 | 0 | 14_Macrophages |
| IFIT3 | 4,106 | 0 | 14_Macrophages |
| USP18 | 3,808 | 0 | 14_Macrophages |
| MX1 | 3,629 | 0 | 14_Macrophages |
| IFI6 | 3,580 | 0 | 14_Macrophages |
| OAS3 | 3,571 | 0 | 14_Macrophages |
| LY6E | 3,510 | 0 | 14_Macrophages |
| CMPK2 | 3,342 | 0 | 14_Macrophages |
| OAS2 | 3,143 | 0 | 14_Macrophages |
| IFI44L | 3,056 | 0 | 14_Macrophages |
| GBP1 | 2,885 | 0 | 14_Macrophages |
| OASL | 2,791 | 0 | 14_Macrophages |
| PTH1R | 8,523 | 0 | 15_PC |
| SLC6A1 | 8,467 | 0 | 15_PC |
| DCN | 8,458 | 0 | 15_PC |
| ABCC9 | 8,421 | 0 | 15_PC |
| HIGD1B | 8,395 | 0 | 15_PC |
| COL3A1 | 8,389 | 0 | 15_PC |
| PRELP | 8,344 | 0 | 15_PC |
| ENPEP | 8,328 | 0 | 15_PC |
| ASPN | 8,290 | 0 | 15_PC |
| PLAC9 | 8,180 | 0 | 15_PC |
| SCG2 | 8,178 | 0 | 15_PC |
| FAM162B | 8,157 | 0 | 15_PC |
| NDUFA4L2 | 8,143 | 0 | 15_PC |
| LAMC3 | 8,084 | 0 | 15_PC |
| CD248 | 8,081 | 0 | 15_PC |
| CD3E | 9,965 | 0 | 16_T_cells |
| LINC01871 | 9,933 | 0 | 16_T_cells |
| TRBC2 | 9,779 | 0 | 16_T_cells |
| CD3D | 9,775 | 0 | 16_T_cells |
| GZMH | 9,680 | 0 | 16_T_cells |
| TRBC1 | 9,658 | 0 | 16_T_cells |
| SAMD3 | 9,649 | 0 | 16_T_cells |
| SH2D1A | 9,599 | 0 | 16_T_cells |
| CD3G | 9,553 | 0 | 16_T_cells |
| CD2 | 9,326 | 0 | 16_T_cells |
| CD8A | 9,312 | 0 | 16_T_cells |
| GZMM | 9,035 | 0 | 16_T_cells |
| LCK | 9,009 | 0 | 16_T_cells |
| IL2RB | 8,950 | 0 | 16_T_cells |
| BCL11B | 8,946 | 0 | 16_T_cells |
| CD79A | 7,854 | 0 | 17_B_cells |
| IGKC | 7,833 | 2.03202654382765e-75 | 17_B_cells |
| JCHAIN | 7,779 | 0 | 17_B_cells |
| IGHG1 | 7,639 | 5.02948877205707e-276 | 17_B_cells |
| FCER1A | 6,624 | 0 | 17_B_cells |
| AFF3 | 5,544 | 0 | 17_B_cells |
| IRF4 | 4,903 | 0 | 17_B_cells |
| BCL11A | 4,272 | 0 | 17_B_cells |
| SLC38A1 | 4,154 | 0 | 17_B_cells |
| SEL1L3 | 3,434 | 2.34437738920469e-182 | 17_B_cells |
| AREG | 3,349 | 4.91080715290422e-249 | 17_B_cells |
| PLAC8 | 3,247 | 4.74004943281644e-159 | 17_B_cells |
| ACAP1 | 2,824 | 1.06008059501084e-203 | 17_B_cells |
| CD52 | 2,735 | 2.20522118618536e-160 | 17_B_cells |
| BIRC3 | 2,540 | 8.39423317786221e-155 | 17_B_cells |
| FABP7 | 8,250 | 0 | 18_Astrocytes |
| GPM6A | 7,920 | 0 | 18_Astrocytes |
| TUBB2B | 7,434 | 0 | 18_Astrocytes |
| C1orf61 | 7,422 | 0 | 18_Astrocytes |
| MT3 | 7,413 | 0 | 18_Astrocytes |
| PCSK1N | 7,228 | 0 | 18_Astrocytes |
| CLU | 6,895 | 0 | 18_Astrocytes |
| GPM6B | 6,189 | 0 | 18_Astrocytes |
| MAP1B | 5,868 | 4.0709023494999e-202 | 18_Astrocytes |
| PTN | 5,609 | 0 | 18_Astrocytes |
| CRYAB | 5,587 | 4.7211833477178e-198 | 18_Astrocytes |
| IGFBP2 | 5,574 | 0 | 18_Astrocytes |
| CPE | 5,238 | 2.3429959750241e-199 | 18_Astrocytes |
| HOPX | 5,220 | 8.77218546803532e-141 | 18_Astrocytes |
| CNN3 | 4,629 | 1.3161799769553e-155 | 18_Astrocytes |
