# Supplementary material for: Pericytes change function depending on glioblastoma vicinity: emphasis on immune regulation
Source: Mol Oncol. 2025 Jul 17;19(9):2491–514. doi: 10.1002/1878-0261.70095 (PMC12420362; doi:10.1002/1878-0261.70095)
Supplement: Supplementary file 12 — Table S4. (A) Table listing the DEGs between mural cells in the tumor core and the contralateral hemisphere. (B) Table listing the upregulated DEGs in human mural cells in the tumor core vs non‐malignant region. (C) Table listing the 228 shared genes between mouse and human mural cells. [file MOL2-19-2491-s001.docx]

Supplementary Table 4

A) Table listing the DEGs between mural cells in the tumor core and the contralateral hemisphere.

| gene | avg_log2FC | p_val_adj |
| --- | --- | --- |
| 2900097C17Rik | 0,208 | 1 |
| 4632427E13Rik | 0,483 | 1 |
| 4833420G17Rik | 3,432 | 1 |
| 4932438A13Rik | 0,253 | 1 |
| 9930111J21Rik2 | 2,302 | 1 |
| Abcc5 | 3,667 | 1 |
| Abhd2 | 1,875 | 1 |
| Ablim1 | 1,454 | 1 |
| Abr | 0,751 | 1 |
| Acin1 | 0,698 | 1 |
| Acsl3 | 0,778 | 1 |
| Actn1 | 0,240 | 1 |
| Adamts1 | 1,275 | 1 |
| Adipor1 | 1,045 | 1 |
| Afap1 | 0,308 | 1 |
| Aftph | 2,353 | 1 |
| Ahsa1 | 0,337 | 1 |
| Akap13 | 1,036 | 1 |
| Akap8 | 1,853 | 1 |
| Akap8l | 3,620 | 1 |
| Akap9 | 0,483 | 1 |
| Akr1a1 | 0,734 | 1 |
| Ank2 | 1,305 | 1 |
| Ankhd1 | 1,436 | 1 |
| Ankrd10 | 0,658 | 1 |
| Ankrd11 | 0,717 | 1 |
| Ankrd17 | 0,938 | 1 |
| Ankrd40 | 1,059 | 1 |
| Ap2b1 | 1,633 | 1 |
| Apcdd1 | 0,896 | 1 |
| Apold1 | 0,752 | 1 |
| App | 0,279 | 1 |
| Arcn1 | 0,350 | 1 |
| Arf1 | 0,408 | 1 |
| Arf4 | 0,802 | 1 |
| Arhgap17 | 5,856 | 1 |
| Arhgap31 | 1,182 | 1 |
| Arhgap5 | 1,339 | 1 |
| Arhgef1 | 4,376 | 1 |
| Arhgef12 | 0,130 | 1 |
| Arhgef2 | 0,751 | 1 |
| Arid1a | 0,895 | 1 |
| Arid5a | 0,947 | 1 |
| Arl13b | 1,590 | 1 |
| Arl2bp | 1,026 | 1 |
| Arl4a | 1,219 | 1 |
| Arl5b | 1,508 | 1 |
| Arntl | 0,650 | 1 |
| Arpc1a | 1,190 | 1 |
| Arpc3 | 0,648 | 1 |
| Arrdc3 | 1,223 | 1 |
| As3mt | 2,162 | 1 |
| Asah1 | 0,983 | 1 |
| Ash1l | 0,500 | 1 |
| Asxl2 | 1,535 | 1 |
| Atad2b | 2,036 | 1 |
| Atf3 | 0,754 | 1 |
| Atf4 | 0,966 | 1 |
| Atf7ip | 1,002 | 1 |
| Atl3 | 0,543 | 1 |
| Atp10a | 3,125 | 1 |
| Atp11a | 2,624 | 1 |
| Atp1a1 | 1,475 | 1 |
| Atp2b4 | 0,144 | 1 |
| Atp6v0a1 | 4,359 | 1 |
| Atp6v0b | 1,740 | 1 |
| Atp6v1h | 0,756 | 1 |
| Atrx | 0,390 | 1 |
| Atxn2l | 1,619 | 1 |
| Avpi1 | 0,447 | 1 |
| AY036118 | 0,800 | 1 |
| Azin1 | 1,909 | 1 |
| B2m | 1,369 | 1 |
| Bach1 | 0,803 | 1 |
| Baz1b | 0,667 | 1 |
| Baz2b | 0,998 | 1 |
| BC005537 | 1,710 | 1 |
| Bcas2 | 0,855 | 1 |
| Bclaf1 | 0,875 | 1 |
| Becn1 | 1,564 | 1 |
| Bhlhe40 | 1,192 | 1 |
| Bin1 | 6,328 | 1 |
| Bmpr2 | 0,554 | 1 |
| Bptf | 1,621 | 1 |
| Brd2 | 1,219 | 1 |
| Brd9 | 2,313 | 1 |
| Bsdc1 | 3,249 | 1 |
| Bsg | 1,429 | 1 |
| Btg2 | 0,943 | 1 |
| Calm2 | 0,285 | 1 |
| Calr | 0,198 | 1 |
| Calu | 0,454 | 1 |
| Camk1 | 0,469 | 1 |
| Canx | 0,372 | 1 |
| Capn2 | 0,718 | 1 |
| Capza2 | 1,291 | 1 |
| Cbx4 | 1,531 | 1 |
| Ccar1 | 0,725 | 1 |
| Ccdc141 | 1,807 | 1 |
| Ccdc50 | 0,572 | 1 |
| Ccnl1 | 2,282 | 1 |
| Ccnl2 | 0,948 | 1 |
| Ccnt1 | 1,714 | 1 |
| Cct6a | 0,175 | 1 |
| Cd164 | 0,611 | 1 |
| Cd2ap | 2,250 | 1 |
| Cdc37 | 0,436 | 1 |
| Cdc37l1 | 1,133 | 1 |
| Cdc42 | 0,118 | 1 |
| Cdc73 | 1,194 | 1 |
| Cdk11b | 2,044 | 1 |
| Cdk12 | 0,921 | 1 |
| Cdkn1a | 1,622 | 1 |
| Cebpb | 1,263 | 1 |
| Cebpd | 0,298 | 1 |
| Cebpg | 2,740 | 1 |
| Cep170 | 1,475 | 1 |
| Cers2 | 0,651 | 1 |
| Cfl2 | 0,570 | 1 |
| Chchd3 | 0,434 | 1 |
| Chd2 | 0,687 | 1 |
| Chd6 | 0,396 | 1 |
| Chd8 | 1,142 | 1 |
| Chd9 | 3,357 | 1 |
| Cherp | 1,224 | 1 |
| Chka | 7,263 | 1 |
| Chmp3 | 0,407 | 1 |
| Chuk | 5,849 | 1 |
| Ckb | 0,142 | 1 |
| Clasp2 | 1,068 | 1 |
| Clip1 | 1,754 | 1 |
| Clk1 | 1,936 | 1 |
| Clk4 | 1,825 | 1 |
| Cltb | 0,448 | 1 |
| Cltc | 1,547 | 1 |
| Cmtm6 | 2,065 | 1 |
| Cnbp | 0,251 | 1 |
| Col27a1 | 2,506 | 1 |
| Col4a2 | 0,183 | 1 |
| Col4a5 | 0,686 | 1 |
| Colgalt1 | 1,388 | 1 |
| Coq10b | 0,882 | 1 |
| Cr1l | 1,165 | 1 |
| Crispld2 | 2,107 | 1 |
| Crk | 0,842 | 1 |
| Csde1 | 0,435 | 1 |
| Csnk1a1 | 0,437 | 1 |
| Csrnp1 | 1,309 | 1 |
| Ctnnb1 | 0,697 | 1 |
| Ctsa | 1,095 | 1 |
| Ctsb | 2,428 | 1 |
| Cul1 | 1,266 | 1 |
| Cux1 | 1,032 | 1 |
| Cxcl12 | 2,154 | 1 |
| Cxxc5 | 2,271 | 1 |
| Cyfip1 | 2,270 | 1 |
| Dab2ip | 0,431 | 1 |
| Dbi | 0,333 | 1 |
| Ddit3 | 1,162 | 1 |
| Ddit4 | 1,008 | 1 |
| Ddx17 | 0,479 | 1 |
| Ddx21 | 0,723 | 1 |
| Ddx39b | 0,747 | 1 |
| Ddx3x | 0,747 | 1 |
| Ddx3y | 0,828 | 1 |
| Ddx5 | 1,147 | 8.02177445414327e-05 |
| Ddx6 | 0,282 | 1 |
| Dennd4c | 1,087 | 1 |
| Dennd5b | 0,978 | 1 |
| Derl1 | 0,177 | 1 |
| Des | 0,311 | 1 |
| Dgkb | 0,468 | 1 |
| Dhx9 | 1,397 | 1 |
| Dip2b | 3,784 | 1 |
| Dleu2 | 2,762 | 1 |
| Dmxl1 | 0,676 | 1 |
| Dnaja1 | 1,367 | 0.0668072200573992 |
| Dnajb1 | 2,271 | 1 |
| Dnajb4 | 1,130 | 1 |
| Dnajb6 | 0,485 | 1 |
| Dnajc10 | 0,535 | 1 |
| Dnm2 | 3,946 | 1 |
| Dnmt3a | 1,746 | 1 |
| Dock10 | 7,956 | 0.469820864404929 |
| Dock9 | 6,590 | 1 |
| Dot1l | 2,061 | 1 |
| Dpp8 | 1,171 | 1 |
| Dpy19l4 | 2,260 | 1 |
| Dpy30 | 0,951 | 1 |
| Dst | 0,664 | 1 |
| Dusp1 | 0,497 | 1 |
| Dusp11 | 0,893 | 1 |
| Dync1h1 | 0,785 | 1 |
| Dync1li2 | 0,819 | 1 |
| Dynlt3 | 0,173 | 1 |
| Dyrk2 | 1,002 | 1 |
| Egr1 | 0,791 | 1 |
| Ehd4 | 0,308 | 1 |
| Eif1a | 0,979 | 1 |
| Eif4a2 | 0,648 | 1 |
| Eif4g1 | 0,608 | 1 |
| Eif4g2 | 0,784 | 1 |
| Eif5 | 0,893 | 1 |
| Eif5a | 0,400 | 1 |
| Emcn | 1,052 | 1 |
| Emd | 0,830 | 1 |
| Eml4 | 1,484 | 1 |
| Entr1 | 2,670 | 1 |
| Eny2 | 0,565 | 1 |
| Epb41l2 | 1,721 | 1 |
| Epc1 | 0,635 | 1 |
| Epn2 | 0,344 | 1 |
| Eprs | 0,930 | 1 |
| Erbin | 2,175 | 1 |
| Errfi1 | 1,292 | 1 |
| Etnk1 | 1,064 | 1 |
| Ewsr1 | 2,121 | 1 |
| Exoc5 | 2,581 | 1 |
| Exoc6b | 1,878 | 1 |
| Extl3 | 0,871 | 1 |
| Fam13c | 1,160 | 1 |
| Fam76a | 0,311 | 1 |
| Fam76b | 1,497 | 1 |
| Fbxl5 | 2,365 | 1 |
| Fbxo32 | 1,029 | 1 |
| Fcho2 | 1,923 | 1 |
| Fchsd2 | 1,684 | 1 |
| Fgf1 | 0,592 | 1 |
| Flna | 0,266 | 1 |
| Flt1 | 4,244 | 1 |
| Fmnl3 | 3,091 | 1 |
| Fos | 0,262 | 1 |
| Fosb | 1,269 | 1 |
| Fosl2 | 1,629 | 1 |
| Foxn3 | 2,298 | 1 |
| Frmd4a | 6,133 | 0.0338731843750804 |
| Frmd4b | 3,774 | 1 |
| Fryl | 1,303 | 1 |
| Fubp1 | 0,663 | 1 |
| Fus | 1,386 | 0.0512189640986303 |
| Fxr1 | 0,766 | 1 |
| Gabarapl2 | 0,367 | 1 |
| Gabbr1 | 2,647 | 1 |
| Gadd45b | 0,881 | 1 |
| Gadd45g | 1,278 | 1 |
| Gapvd1 | 1,425 | 1 |
| Gcc1 | 1,660 | 1 |
| Gcc2 | 1,479 | 1 |
| Gcnt2 | 0,170 | 1 |
| Gem | 1,119 | 1 |
| Ggnbp2 | 1,131 | 1 |
| Gigyf1 | 2,277 | 1 |
| Glg1 | 0,322 | 1 |
| Gls | 1,564 | 1 |
| Glul | 1,608 | 1 |
| Glyr1 | 1,433 | 1 |
| Gm26532 | 0,675 | 1 |
| Gm37494 | 2,358 | 1 |
| Gnai2 | 0,287 | 1 |
| Gnaq | 1,523 | 1 |
| Gnb2 | 0,277 | 1 |
| Gns | 1,516 | 1 |
| Golga4 | 1,679 | 1 |
| Golph3 | 0,465 | 1 |
| Gpbp1 | 0,911 | 1 |
| Gpcpd1 | 1,212 | 1 |
| Gpr146 | 1,862 | 1 |
| Gripap1 | 1,310 | 1 |
| Gtf2a2 | 0,313 | 1 |
| Gtf2i | 0,347 | 1 |
| H2-D1 | 1,102 | 1 |
| H2-K1 | 2,712 | 1 |
| Hacd2 | 0,122 | 1 |
| Hccs | 1,625 | 1 |
| Hdlbp | 1,015 | 1 |
| Hectd1 | 1,323 | 1 |
| Herc2 | 3,982 | 1 |
| Hip1 | 0,643 | 1 |
| Hist1h2bc | 0,658 | 1 |
| Hmgxb4 | 1,001 | 1 |
| Hnrnpa2b1 | 0,883 | 0.00798027901585931 |
| Hnrnpa3 | 0,278 | 1 |
| Hnrnpc | 0,759 | 1 |
| Hnrnpdl | 0,605 | 1 |
| Hnrnpf | 0,247 | 1 |
| Hnrnph1 | 0,944 | 1 |
| Hnrnph2 | 0,554 | 1 |
| Hnrnph3 | 0,843 | 1 |
| Hnrnpl | 0,337 | 1 |
| Hnrnpm | 0,571 | 1 |
| Hnrnpu | 0,732 | 1 |
| Hook3 | 0,478 | 1 |
| Hsp90aa1 | 1,818 | 0.00182937893579609 |
| Hspa1a | 1,291 | 1 |
| Hspa1b | 1,654 | 1 |
| Hspa4 | 0,289 | 1 |
| Hspa8 | 0,654 | 1 |
| Hspd1 | 0,642 | 1 |
| Hsph1 | 3,328 | 0.409911567010555 |
| Huwe1 | 0,320 | 1 |
| Id1 | 0,618 | 1 |
| Id3 | 0,725 | 1 |
| Ier2 | 0,776 | 1 |
| Ier3 | 0,662 | 1 |
| Ier5 | 1,130 | 1 |
| Ifi27 | 0,820 | 1 |
| Ifnar1 | 3,034 | 1 |
| Igf1r | 2,484 | 1 |
| Igf2 | 0,611 | 1 |
| Il6st | 0,138 | 1 |
| Impact | 1,763 | 1 |
| Impdh2 | 1,128 | 1 |
| Ino80d | 1,312 | 1 |
| Ints6 | 1,307 | 1 |
| Ints6l | 7,672 | 1 |
| Ipo8 | 1,190 | 1 |
| Irf1 | 1,349 | 1 |
| Irf2bp2 | 0,709 | 1 |
| Irs2 | 1,236 | 1 |
| Itch | 2,202 | 1 |
| Itgb5 | 1,250 | 1 |
| Itih5 | 1,593 | 1 |
| Itpr2 | 0,228 | 1 |
| Itsn2 | 2,493 | 1 |
| Jmjd1c | 2,318 | 0.153230800301193 |
| Jun | 0,879 | 1 |
| Junb | 0,548 | 1 |
| Jund | 1,064 | 1 |
| Jup | 1,568 | 1 |
| Kansl1l | 2,244 | 1 |
| Kat6a | 1,930 | 1 |
| Kcne4 | 0,239 | 1 |
| Kcnq1ot1 | 0,388 | 1 |
| Kdm2a | 1,220 | 1 |
| Kdm6b | 1,105 | 1 |
| Kdm7a | 3,185 | 1 |
| Kif13b | 2,201 | 1 |
| Kif1b | 0,946 | 1 |
| Kif5b | 0,225 | 1 |
| Kitl | 0,551 | 1 |
| Klf13 | 1,059 | 1 |
| Klf2 | 1,102 | 1 |
| Klf3 | 1,706 | 1 |
| Klf4 | 1,066 | 1 |
| Klf6 | 0,497 | 1 |
| Klf7 | 0,709 | 1 |
| Klhl9 | 0,651 | 1 |
| Kmt2c | 0,904 | 1 |
| Kmt2e | 1,311 | 1 |
| Kmt5b | 1,285 | 1 |
| Kpna1 | 1,437 | 1 |
| Lama4 | 0,138 | 1 |
| Lamp2 | 0,635 | 1 |
| Lars2 | 0,703 | 1 |
| Lcmt2 | 1,668 | 1 |
| Lcorl | 3,307 | 1 |
| Leprot | 0,671 | 1 |
| Litaf | 1,344 | 1 |
| Lmna | 0,384 | 1 |
| Lrch3 | 1,285 | 1 |
| Lsm8 | 0,658 | 1 |
| Luc7l2 | 0,517 | 1 |
| Macf1 | 0,927 | 1 |
| Maff | 1,587 | 1 |
| Mafg | 3,970 | 1 |
| Mafk | 0,721 | 1 |
| Magoh | 0,571 | 1 |
| Man1a2 | 1,219 | 1 |
| Man2a2 | 3,448 | 1 |
| Map1lc3b | 0,276 | 1 |
| Map3k8 | 2,481 | 1 |
| Map4 | 0,144 | 1 |
| Map4k4 | 1,874 | 1 |
| Mapk1 | 0,740 | 1 |
| Mapk8ip3 | 0,800 | 1 |
| Mapre1 | 0,852 | 1 |
| 07-Mar | 0,293 | 1 |
| Mat2a | 0,953 | 1 |
| Mcl1 | 1,773 | 1 |
| Mdm4 | 0,506 | 1 |
| Mecp2 | 0,471 | 1 |
| Med14 | 2,112 | 1 |
| Mef2a | 1,693 | 1 |
| Mertk | 1,624 | 1 |
| Mff | 0,701 | 1 |
| Mfsd14a | 1,416 | 1 |
| Mfsd14b | 1,768 | 1 |
| Mgat1 | 1,624 | 1 |
| Mgat4a | 2,651 | 1 |
| Mia3 | 1,004 | 1 |
| Mical2 | 0,703 | 1 |
| Midn | 1,322 | 1 |
| Mindy2 | 0,652 | 1 |
| Mir22hg | 1,956 | 1 |
| Mlec | 0,744 | 1 |
| Mlf1 | 1,528 | 1 |
| Mob4 | 0,400 | 1 |
| Mon2 | 3,356 | 1 |
| Morf4l2 | 0,913 | 1 |
| Mpc1 | 0,279 | 1 |
| Mpp7 | 0,464 | 1 |
| Mrfap1 | 0,350 | 1 |
| Msl1 | 1,212 | 1 |
| mt-Co1 | 0,384 | 1 |
| mt-Nd1 | 0,425 | 1 |
| Mt1 | 0,720 | 1 |
| Mtfr1l | 0,926 | 1 |
| Mtus1 | 3,560 | 0.616026828024923 |
| Mycbp2 | 4,058 | 0.493128887719252 |
| Mylip | 0,633 | 1 |
| Mylk | 0,283 | 1 |
| Myo1e | 0,710 | 1 |
| Myo6 | 2,122 | 1 |
| Mysm1 | 0,993 | 1 |
| N4bp2l2 | 1,599 | 1 |
| Naa35 | 2,760 | 1 |
| Naa50 | 1,652 | 1 |
| Nars | 1,555 | 1 |
| Ncl | 0,203 | 1 |
| Ncor1 | 0,419 | 1 |
| Ndel1 | 2,066 | 1 |
| Ndfip2 | 1,429 | 1 |
| Neat1 | 2,100 | 0.17984995493015 |
| Nfat5 | 0,248 | 1 |
| Nfe2l1 | 0,234 | 1 |
| Nfic | 1,338 | 1 |
| Nfkbia | 1,682 | 1 |
| Nfkbiz | 1,631 | 1 |
| Nisch | 0,648 | 1 |
| Nktr | 0,672 | 1 |
| Nr3c1 | 0,348 | 1 |
| Nr4a1 | 0,892 | 1 |
| Nr4a2 | 1,557 | 1 |
| Nr4a3 | 2,244 | 1 |
| Nrd1 | 0,470 | 1 |
| Nrip1 | 1,904 | 1 |
| Nrp1 | 1,698 | 1 |
| Nsd1 | 1,004 | 1 |
| Nsmce2 | 1,402 | 1 |
| Nub1 | 0,543 | 1 |
| Nufip2 | 1,102 | 1 |
| Numa1 | 0,712 | 1 |
| Numb | 7,790 | 1 |
| Nup98 | 1,336 | 1 |
| Odc1 | 1,158 | 1 |
| Olfml2a | 0,586 | 1 |
| Opa3 | 1,170 | 1 |
| Osbpl1a | 3,559 | 1 |
| Osmr | 0,462 | 1 |
| P4ha1 | 0,629 | 1 |
| Pabpc1 | 0,565 | 1 |
| Pafah1b1 | 0,500 | 1 |
| Paip2 | 0,241 | 1 |
| Papola | 0,493 | 1 |
| Pcbp1 | 0,625 | 1 |
| Pcf11 | 1,574 | 1 |
| Pcgf5 | 1,051 | 1 |
| Pcm1 | 0,335 | 1 |
| Pcmtd1 | 1,020 | 1 |
| Pcnp | 0,413 | 1 |
| Pcp4l1 | 0,195 | 1 |
| Pde4b | 0,876 | 1 |
| Pdhb | 1,530 | 1 |
| Pdlim5 | 0,875 | 1 |
| Pds5a | 1,689 | 1 |
| Pdxk | 0,613 | 1 |
| Pea15a | 1,331 | 1 |
| Picalm | 0,683 | 1 |
| Pik3c2a | 0,876 | 1 |
| Pik3r1 | 0,339 | 1 |
| Pim3 | 1,081 | 1 |
| Pip4p1 | 4,085 | 1 |
| Plekha1 | 1,229 | 1 |
| Plekhg3 | 0,384 | 1 |
| Pln | 0,205 | 1 |
| Plod1 | 1,725 | 1 |
| Plod2 | 0,586 | 1 |
| Pmepa1 | 2,836 | 1 |
| Pnisr | 0,108 | 1 |
| Pnn | 0,575 | 1 |
| Pnpla2 | 0,298 | 1 |
| Polg | 2,369 | 1 |
| Pon2 | 1,050 | 1 |
| Ppard | 1,150 | 1 |
| Ppp1cc | 0,531 | 1 |
| Ppp1r12a | 0,164 | 1 |
| Ppp1r15a | 0,861 | 1 |
| Ppp1r2 | 0,383 | 1 |
| Ppp2r5a | 0,215 | 1 |
| Ppp3ca | 1,240 | 1 |
| Ppp6r3 | 2,031 | 1 |
| Prnp | 3,002 | 1 |
| Prpf39 | 0,732 | 1 |
| Prrc2c | 0,412 | 1 |
| Psap | 0,340 | 1 |
| Psma1 | 0,399 | 1 |
| Psmd7 | 0,521 | 1 |
| Ptprg | 3,688 | 1 |
| Puf60 | 1,264 | 1 |
| Pum1 | 0,434 | 1 |
| Pum2 | 0,659 | 1 |
| Pura | 0,682 | 1 |
| Pwwp2a | 2,403 | 1 |
| Pxk | 3,445 | 1 |
| Qdpr | 2,323 | 1 |
| Qk | 2,148 | 0.00363687513402631 |
| Qrich1 | 3,260 | 1 |
| R3hdm1 | 0,330 | 1 |
| Rab10 | 0,413 | 1 |
| Rab22a | 0,465 | 1 |
| Rab5a | 0,527 | 1 |
| Rab5b | 0,912 | 1 |
| Rabgap1 | 0,589 | 1 |
| Rabgef1 | 1,715 | 1 |
| Ralb | 1,839 | 1 |
| Ran | 0,134 | 1 |
| Rap1a | 0,345 | 1 |
| Rapgef6 | 3,253 | 1 |
| Rasa1 | 2,692 | 1 |
| Rasd1 | 1,116 | 1 |
| Rasgrp3 | 3,438 | 1 |
| Rassf1 | 1,272 | 1 |
| Rbbp4 | 1,164 | 1 |
| Rbm25 | 1,445 | 1 |
| Rbm39 | 0,356 | 1 |
| Rbm5 | 0,441 | 1 |
| Rbm6 | 0,617 | 1 |
| Reep3 | 0,234 | 1 |
| Relb | 2,864 | 1 |
| Rheb | 0,250 | 1 |
| Rhoa | 0,227 | 1 |
| Rhob | 1,153 | 0.0147499237280545 |
| Rhoc | 0,107 | 1 |
| Rictor | 0,162 | 1 |
| Ripk1 | 2,060 | 1 |
| Rnd1 | 1,923 | 1 |
| Rnf14 | 0,337 | 1 |
| Rnf150 | 0,546 | 1 |
| Rnf152 | 0,312 | 1 |
| Rnf19a | 1,254 | 1 |
| Rnf20 | 1,254 | 1 |
| Rnf216 | 2,843 | 1 |
| Rnf8 | 2,036 | 1 |
| Rprd2 | 0,736 | 1 |
| Rrbp1 | 0,895 | 1 |
| Rsf1 | 0,909 | 1 |
| Rsrc2 | 1,145 | 1 |
| Rsrp1 | 0,644 | 1 |
| Runx1 | 4,951 | 1 |
| Safb2 | 2,729 | 1 |
| Sar1a | 1,019 | 1 |
| Saraf | 0,635 | 1 |
| Sash1 | 0,128 | 1 |
| Sat1 | 1,262 | 1 |
| Sbds | 0,857 | 1 |
| Sbf2 | 1,033 | 1 |
| Sbno1 | 1,070 | 1 |
| Sbno2 | 1,987 | 1 |
| Scaf11 | 0,328 | 1 |
| Scd2 | 1,826 | 1 |
| Scoc | 3,207 | 1 |
| Scp2 | 0,359 | 1 |
| Sdc4 | 0,686 | 1 |
| Sdcbp | 0,847 | 1 |
| Selenop | 2,306 | 1 |
| Sema4c | 3,661 | 1 |
| Senp2 | 7,662 | 0.469820864404929 |
| Serbp1 | 0,327 | 1 |
| Serpine2 | 0,305 | 1 |
| Sertad1 | 1,052 | 1 |
| Setd2 | 2,709 | 1 |
| Setd3 | 0,686 | 1 |
| Sf3b1 | 0,931 | 1 |
| Sf3b2 | 0,262 | 1 |
| Sfpq | 1,425 | 1 |
| Sgk1 | 1,883 | 1 |
| Sgms1 | 5,126 | 1 |
| Sh3gl1 | 1,818 | 1 |
| Siah2 | 2,591 | 1 |
| Sik1 | 0,339 | 1 |
| Sik2 | 0,883 | 1 |
| Sik3 | 0,387 | 1 |
| Sike1 | 0,236 | 1 |
| Sipa1 | 2,532 | 1 |
| Ski | 0,827 | 1 |
| Skil | 7,217 | 1 |
| Skp1a | 0,556 | 1 |
| Slbp | 0,729 | 1 |
| Slc25a25 | 1,046 | 1 |
| Slc2a1 | 3,949 | 1 |
| Slc30a9 | 2,313 | 1 |
| Slc38a2 | 1,063 | 1 |
| Slc39a10 | 1,918 | 1 |
| Slc3a2 | 1,647 | 1 |
| Slc5a3 | 1,308 | 1 |
| Slc7a1 | 7,065 | 1 |
| Slc9a3r2 | 0,485 | 1 |
| Slmap | 0,598 | 1 |
| Smad7 | 4,091 | 1 |
| Smarca5 | 0,673 | 1 |
| Smchd1 | 0,875 | 1 |
| Smg1 | 0,868 | 1 |
| Smim10l1 | 0,244 | 1 |
| Sncg | 0,407 | 1 |
| Snhg1 | 0,505 | 1 |
| Snrnp70 | 2,213 | 1 |
| Snx17 | 0,697 | 1 |
| Snx3 | 0,544 | 1 |
| Socs3 | 0,654 | 1 |
| Son | 0,659 | 1 |
| Sp1 | 0,704 | 1 |
| Spag9 | 0,485 | 1 |
| Spint2 | 0,631 | 1 |
| Sptan1 | 0,547 | 1 |
| Sptbn1 | 0,598 | 1 |
| Sptssa | 0,410 | 1 |
| Srek1 | 0,682 | 1 |
| Srpk2 | 0,573 | 1 |
| Srrm1 | 1,076 | 1 |
| Srrm2 | 0,838 | 1 |
| Srrt | 2,167 | 1 |
| Srsf1 | 1,085 | 1 |
| Srsf11 | 1,626 | 1 |
| Srsf2 | 1,078 | 1 |
| Srsf3 | 0,753 | 1 |
| Srsf5 | 0,504 | 1 |
| Srsf6 | 2,012 | 1 |
| Srsf7 | 0,672 | 1 |
| Stag2 | 0,496 | 1 |
| Stat3 | 1,125 | 1 |
| Stip1 | 0,561 | 1 |
| Stk38 | 2,122 | 1 |
| Stk38l | 0,959 | 1 |
| Stom | 0,801 | 1 |
| Stx12 | 0,960 | 1 |
| Stx4a | 0,865 | 1 |
| Stx5a | 2,014 | 1 |
| Stxbp3 | 0,741 | 1 |
| Sumo1 | 0,346 | 1 |
| Sun2 | 0,665 | 1 |
| Swap70 | 4,378 | 1 |
| Swt1 | 1,089 | 1 |
| Syne1 | 0,306 | 1 |
| Syne2 | 0,359 | 1 |
| Synm | 0,639 | 1 |
| Tab2 | 2,030 | 1 |
| Tacc1 | 0,305 | 1 |
| Taf15 | 1,402 | 1 |
| Taf1d | 5,347 | 0.959623356518994 |
| Taok3 | 1,140 | 1 |
| Tardbp | 0,632 | 1 |
| Tasor | 1,180 | 1 |
| Taz | 1,495 | 1 |
| Tcf25 | 0,832 | 1 |
| Tdp2 | 1,280 | 1 |
| Tet3 | 0,595 | 1 |
| Tfrc | 3,904 | 1 |
| Tgfbr2 | 1,034 | 1 |
| Tgoln1 | 0,809 | 1 |
| Thbs1 | 2,303 | 1 |
| Thoc2 | 0,352 | 1 |
| Thrap3 | 0,825 | 1 |
| Tial1 | 1,310 | 1 |
| Tifa | 3,180 | 1 |
| Tiparp | 0,628 | 1 |
| Tle3 | 3,337 | 1 |
| Tm2d1 | 0,836 | 1 |
| Tm2d2 | 0,834 | 1 |
| Tm9sf2 | 0,313 | 1 |
| Tm9sf3 | 0,417 | 1 |
| Tmed9 | 0,438 | 1 |
| Tmem63a | 6,826 | 1 |
| Tmpo | 0,805 | 1 |
| Tmx3 | 0,335 | 1 |
| Tmx4 | 1,338 | 1 |
| Tnfrsf1a | 0,659 | 1 |
| Tnpo1 | 0,553 | 1 |
| Tnpo3 | 1,053 | 1 |
| Tnrc6a | 1,164 | 1 |
| Tob1 | 0,900 | 1 |
| Tob2 | 1,163 | 1 |
| Tpm3 | 1,822 | 1 |
| Tpm4 | 0,265 | 1 |
| Tpp1 | 1,176 | 1 |
| Tra2a | 1,491 | 1 |
| Tra2b | 2,181 | 0.0190263511408106 |
| Trim8 | 3,006 | 1 |
| Trip11 | 0,237 | 1 |
| Trpc4ap | 2,371 | 1 |
| Trps1 | 0,172 | 1 |
| Trrap | 2,785 | 1 |
| Tsc22d2 | 3,756 | 0.260372180580308 |
| Tsc22d3 | 1,738 | 1 |
| Tsc22d4 | 1,500 | 1 |
| Tsn | 0,336 | 1 |
| Tubgcp5 | 3,165 | 1 |
| Tut4 | 1,367 | 1 |
| Tut7 | 2,876 | 1 |
| Txnrd1 | 1,106 | 1 |
| Uaca | 0,376 | 1 |
| Uap1 | 2,926 | 1 |
| Ubap2l | 0,808 | 1 |
| Ubc | 1,088 | 1 |
| Ube2d3 | 0,889 | 1 |
| Ube2q1 | 2,224 | 1 |
| Ube2r2 | 0,849 | 1 |
| Ubl3 | 2,595 | 1 |
| Ubn2 | 2,224 | 1 |
| Ubr2 | 0,535 | 1 |
| Ubr4 | 1,130 | 1 |
| Ubtf | 1,805 | 1 |
| Ufc1 | 0,906 | 1 |
| Upf3b | 1,048 | 1 |
| Uri1 | 1,302 | 1 |
| Usp14 | 1,615 | 1 |
| Usp16 | 1,185 | 1 |
| Usp19 | 0,625 | 1 |
| Usp24 | 1,971 | 1 |
| Usp34 | 0,636 | 1 |
| Usp37 | 0,791 | 1 |
| Usp7 | 1,830 | 1 |
| Usp9x | 0,820 | 1 |
| Uvrag | 2,386 | 1 |
| Vmp1 | 1,032 | 1 |
| Vps13d | 2,030 | 1 |
| Washc2 | 1,985 | 1 |
| Washc4 | 4,697 | 1 |
| Wdr1 | 0,277 | 1 |
| Wdr26 | 0,546 | 1 |
| Wdr44 | 3,713 | 1 |
| Wdr70 | 1,887 | 1 |
| Wnk1 | 1,931 | 1 |
| Wrn | 0,696 | 1 |
| Wsb1 | 3,018 | 0.000679121681383032 |
| Yap1 | 0,681 | 1 |
| Ybx1 | 0,125 | 1 |
| Yme1l1 | 1,055 | 1 |
| Ypel5 | 1,413 | 1 |
| Ythdc1 | 1,434 | 1 |
| Ythdf3 | 1,222 | 1 |
| Yy1 | 0,728 | 1 |
| Zbtb7a | 1,094 | 1 |
| Zc3h14 | 0,924 | 1 |
| Zc3h18 | 0,958 | 1 |
| Zc3h7a | 1,666 | 1 |
| Zcchc7 | 2,135 | 1 |
| Zeb2 | 0,246 | 1 |
| Zfand5 | 1,939 | 1 |
| Zfand6 | 0,779 | 1 |
| Zfhx3 | 0,344 | 1 |
| Zfp148 | 0,904 | 1 |
| Zfp207 | 0,916 | 1 |
| Zfp280d | 1,186 | 1 |
| Zfp292 | 1,116 | 1 |
| Zfp36 | 0,459 | 1 |
| Zfp36l1 | 0,618 | 1 |
| Zfp36l2 | 0,290 | 1 |
| Zfp445 | 1,652 | 1 |
| Zfp451 | 1,741 | 1 |
| Zfp638 | 1,199 | 1 |
| Zfp800 | 1,361 | 1 |
| Zfp869 | 1,340 | 1 |
| Zfr | 0,850 | 1 |
| Zkscan3 | 1,767 | 1 |
| Zmiz1 | 2,444 | 1 |
| Zyg11b | 0,590 | 1 |
| Zyx | 1,214 | 1 |

B) Table listing the upregulated DEGs in human mural cells in the tumor core vs non-malignant region.

| gene | avg_log2FC | p_val_adj |
| --- | --- | --- |
| A1BG | 0,527 | 1 |
| ABCC9 | 0,382 | 1 |
| ABCE1 | 0,362 | 1 |
| ABCF1 | 1,077 | 1 |
| ABCG2 | 2,397 | 1 |
| ABHD5 | 0,850 | 1 |
| ABL2 | 1,494 | 0,507045157 |
| ABRACL | 2,626 | 0,059052443 |
| AC005261,1 | 0,799 | 1 |
| AC020656,1 | 5,136 | 0,413463609 |
| AC020916,1 | 0,414 | 1 |
| AC068491,3 | 2,833 | 1 |
| AC092069,1 | 2,270 | 1 |
| AC108463,3 | 3,605 | 1 |
| AC245595,1 | 2,696 | 1 |
| ACADVL | 0,196 | 1 |
| ACBD3 | 0,504 | 1 |
| ACIN1 | 0,109 | 1 |
| ACLY | 1,779 | 0,769989263 |
| ACOT13 | 1,224 | 1 |
| ACOT9 | 0,946 | 1 |
| ACP1 | 0,699 | 1 |
| ACP5 | 2,288 | 1 |
| ACSL4 | 0,827 | 1 |
| ACTA2 | 0,291 | 1 |
| ACTG1 | 0,685 | 0,067812262 |
| ACTN1 | 1,607 | 0,007673163 |
| ACTR1A | 0,720 | 1 |
| ACTR2 | 0,628 | 1 |
| ACTR3 | 1,314 | 0,001285006 |
| ADA | 0,849 | 1 |
| ADAM10 | 0,938 | 1 |
| ADAM12 | 4,227 | 0,000200399 |
| ADAM17 | 0,649 | 1 |
| ADAM19 | 5,886 | 1 |
| ADAM9 | 1,643 | 0,592449874 |
| ADAMTS1 | 0,524 | 1 |
| ADAMTS4 | 2,878 | 0,051300717 |
| ADAMTS9 | 2,316 | 0,982636283 |
| ADD3 | 1,437 | 0,074021237 |
| ADGRE2 | 1,851 | 1 |
| ADGRF5 | 1,398 | 1 |
| ADGRL4 | 4,528 | 1 |
| ADM | 2,405 | 1 |
| ADNP | 1,003 | 1 |
| ADORA2B | 0,740 | 1 |
| ADRM1 | 0,809 | 1 |
| AEBP1 | 3,212 | 0,001310739 |
| AFAP1 | 1,027 | 1 |
| AGFG1 | 0,936 | 1 |
| AGPAT5 | 0,908 | 1 |
| AGRN | 0,733 | 1 |
| AHCTF1 | 0,730 | 1 |
| AHCY | 1,219 | 1 |
| AHR | 2,991 | 0,015238767 |
| AHSA1 | 0,573 | 1 |
| AIDA | 0,232 | 1 |
| AK3 | 0,748 | 1 |
| AKAP12 | 1,255 | 1 |
| AKIRIN2 | 0,515 | 1 |
| AKR1A1 | 0,509 | 1 |
| AKT1 | 0,957 | 1 |
| AKT1S1 | 1,507 | 1 |
| ALCAM | 1,267 | 1 |
| ALOX5AP | 1,556 | 0,359875221 |
| ALYREF | 1,735 | 1 |
| AMOTL1 | 1,689 | 0,101549441 |
| ANAPC11 | 0,193 | 1 |
| ANAPC5 | 1,065 | 1 |
| ANGPT2 | 4,585 | 1,03E-05 |
| ANGPTL4 | 1,490 | 0,008658771 |
| ANKH | 0,981 | 1 |
| ANKIB1 | 1,027 | 1 |
| ANKLE2 | 0,903 | 1 |
| ANKRD10 | 0,334 | 1 |
| ANKRD11 | 0,257 | 1 |
| ANKRD28 | 3,966 | 0,054615932 |
| ANKRD40 | 1,151 | 1 |
| ANP32E | 0,537 | 1 |
| ANTXR1 | 1,885 | 0,122718906 |
| ANXA1 | 3,529 | 2,76E-08 |
| ANXA2 | 1,780 | 1,43E-05 |
| ANXA5 | 0,304 | 1 |
| ANXA6 | 0,472 | 1 |
| AP1M1 | 1,599 | 1 |
| AP1S1 | 1,476 | 1 |
| AP2A1 | 0,102 | 1 |
| AP2S1 | 1,586 | 0,004626441 |
| AP3B1 | 0,208 | 1 |
| AP3S1 | 1,196 | 1 |
| APC | 0,378 | 1 |
| APEX1 | 0,733 | 1 |
| APH1A | 0,477 | 1 |
| APLN | 6,124 | 1 |
| APLP2 | 0,652 | 1 |
| APOC1 | 1,846 | 1,17E-08 |
| APOE | 0,573 | 1 |
| APOPT1 | 1,860 | 1 |
| APP | 1,156 | 1 |
| APRT | 0,888 | 0,974823776 |
| ARCN1 | 0,393 | 1 |
| AREG | 1,547 | 1 |
| ARF1 | 0,191 | 1 |
| ARF4 | 0,657 | 1 |
| ARF6 | 0,601 | 1 |
| ARFGAP3 | 1,178 | 1 |
| ARFGEF1 | 0,637 | 1 |
| ARHGAP15 | 0,864 | 1 |
| ARHGAP18 | 3,505 | 0,000120651 |
| ARHGAP21 | 0,387 | 1 |
| ARHGAP26 | 0,904 | 1 |
| ARHGDIA | 0,785 | 1 |
| ARHGDIB | 0,526 | 1 |
| ARHGEF12 | 0,680 | 1 |
| ARHGEF7 | 0,699 | 1 |
| ARID1A | 0,294 | 1 |
| ARIH1 | 0,865 | 1 |
| ARL2BP | 0,215 | 1 |
| ARL3 | 0,166 | 1 |
| ARL4A | 1,199 | 1 |
| ARL4C | 1,724 | 1 |
| ARL6IP1 | 0,407 | 1 |
| ARL8B | 0,850 | 1 |
| ARMCX3 | 0,195 | 1 |
| ARMT1 | 1,196 | 1 |
| ARPC1B | 0,380 | 1 |
| ARPC2 | 0,988 | 1 |
| ARPC3 | 0,812 | 0,066135515 |
| ARPC4 | 1,161 | 0,063268874 |
| ARPC5 | 0,843 | 0,009963829 |
| ARPC5L | 0,494 | 1 |
| ASAH1 | 0,188 | 1 |
| ASAP1 | 1,206 | 1 |
| ASAP2 | 0,415 | 1 |
| ASCC3 | 0,586 | 1 |
| ASPH | 0,869 | 1 |
| ASPN | 3,818 | 0,068823408 |
| ASXL1 | 1,062 | 1 |
| ATAD2 | 1,506 | 1 |
| ATF5 | 1,249 | 1 |
| ATF6 | 1,021 | 1 |
| ATG12 | 1,083 | 1 |
| ATG3 | 0,378 | 1 |
| ATL3 | 0,688 | 1 |
| ATOX1 | 0,865 | 1 |
| ATP13A3 | 1,426 | 1 |
| ATP1A1 | 0,909 | 1 |
| ATP1B3 | 0,593 | 1 |
| ATP2B1 | 0,902 | 1 |
| ATP2B4 | 0,923 | 1 |
| ATP2C1 | 0,749 | 1 |
| ATP5F1A | 0,132 | 1 |
| ATP5F1C | 0,598 | 1 |
| ATP5F1E | 0,177 | 1 |
| ATP5MC2 | 0,321 | 1 |
| ATP5MF | 0,387 | 1 |
| ATP6AP2 | 0,540 | 1 |
| ATP6V0B | 0,929 | 0,15959534 |
| ATP6V0D1 | 0,211 | 1 |
| ATP6V1A | 0,949 | 1 |
| ATP6V1C1 | 0,585 | 1 |
| ATP6V1D | 0,115 | 0,885212359 |
| ATP6V1G1 | 0,221 | 1 |
| AUP1 | 0,521 | 1 |
| BACE2 | 0,710 | 1 |
| BACH1 | 0,155 | 1 |
| BAG6 | 0,813 | 1 |
| BASP1 | 1,712 | 1 |
| BAX | 0,513 | 1 |
| BAZ1A | 1,438 | 1 |
| BAZ1B | 0,136 | 1 |
| BCAP29 | 0,248 | 1 |
| BCAP31 | 1,355 | 0,412895145 |
| BCAT1 | 2,183 | 1 |
| BCKDK | 0,390 | 1 |
| BCL2A1 | 1,048 | 0,77885353 |
| BCL7C | 0,541 | 1 |
| BCLAF1 | 0,607 | 1 |
| BGN | 1,355 | 0,068819606 |
| BHLHE40 | 2,634 | 0,065121987 |
| BID | 0,789 | 1 |
| BIRC6 | 0,723 | 1 |
| BLOC1S2 | 0,889 | 1 |
| BMP1 | 2,904 | 0,781033223 |
| BMPR2 | 0,514 | 1 |
| BOLA3 | 1,183 | 1 |
| BRK1 | 0,268 | 1 |
| BTF3L4 | 0,705 | 1 |
| BUB3 | 0,337 | 1 |
| BUD31 | 0,421 | 1 |
| BZW1 | 1,355 | 0,511398715 |
| BZW2 | 2,776 | 1 |
| C15orf48 | 4,430 | 1 |
| C16orf72 | 0,219 | 1 |
| C19orf24 | 1,232 | 1 |
| C19orf53 | 0,146 | 1 |
| C1orf122 | 1,262 | 0,000238029 |
| C1orf54 | 0,191 | 1 |
| C1orf56 | 0,726 | 1 |
| C1QA | 0,127 | 1 |
| C1QB | 0,592 | 1 |
| C1QBP | 0,665 | 1 |
| C1QTNF6 | 3,596 | 1 |
| C1R | 1,421 | 1 |
| C4orf48 | 1,650 | 0,004940344 |
| C5AR1 | 0,428 | 1 |
| C5orf15 | 1,210 | 1 |
| C6orf62 | 0,164 | 1 |
| C8orf33 | 0,526 | 1 |
| C9orf16 | 1,113 | 1 |
| CA2 | 0,815 | 1 |
| CADM1 | 2,844 | 0,000569775 |
| CADM4 | 1,430 | 1 |
| CALCRL | 2,638 | 1 |
| CALM1 | 0,393 | 1 |
| CALR | 2,096 | 2,78E-12 |
| CALU | 1,233 | 0,022912524 |
| CAMSAP2 | 0,478 | 1 |
| CANX | 0,932 | 0,165344813 |
| CAP1 | 0,761 | 1 |
| CAPG | 1,344 | 1 |
| CAPN2 | 0,311 | 1 |
| CAPNS1 | 0,695 | 1 |
| CAPRIN1 | 0,544 | 1 |
| CAPZA1 | 1,368 | 1 |
| CAPZA2 | 0,612 | 1 |
| CARHSP1 | 0,659 | 1 |
| CASK | 1,606 | 1 |
| CASP3 | 1,387 | 1 |
| CBLB | 1,488 | 1 |
| CBX3 | 0,676 | 1 |
| CCAR1 | 0,475 | 1 |
| CCDC102B | 2,293 | 0,000360333 |
| CCDC124 | 0,251 | 1 |
| CCDC167 | 2,982 | 1 |
| CCDC3 | 0,847 | 1 |
| CCDC6 | 0,782 | 1 |
| CCDC80 | 0,483 | 1 |
| CCDC88A | 0,354 | 1 |
| CCL2 | 2,511 | 9,29E-06 |
| CCL20 | 4,510 | 5,12E-07 |
| CCND2 | 0,311 | 1 |
| CCNL2 | 1,074 | 1 |
| CCNY | 0,360 | 1 |
| CCPG1 | 0,673 | 1 |
| CCRL2 | 2,333 | 1 |
| CCT2 | 0,807 | 1 |
| CCT3 | 0,322 | 1 |
| CCT4 | 0,567 | 1 |
| CCT5 | 1,018 | 1 |
| CCT6A | 0,690 | 1 |
| CCT8 | 0,830 | 1 |
| CD14 | 0,150 | 1 |
| CD163 | 2,838 | 4,73E-09 |
| CD164 | 0,398 | 1 |
| CD248 | 1,758 | 0,425289629 |
| CD276 | 1,253 | 0,918339206 |
| CD40 | 0,582 | 1 |
| CD44 | 1,702 | 4,70E-06 |
| CD55 | 1,978 | 1 |
| CD68 | 1,015 | 1 |
| CD93 | 5,780 | 0,661533913 |
| CD99 | 0,288 | 1 |
| CDC26 | 0,459 | 1 |
| CDC27 | 0,554 | 1 |
| CDC37 | 0,518 | 1 |
| CDC42BPA | 0,233 | 1 |
| CDC42EP1 | 0,587 | 1 |
| CDC42EP5 | 1,918 | 1 |
| CDC42SE1 | 0,490 | 1 |
| CDC5L | 0,314 | 1 |
| CDH11 | 1,349 | 1 |
| CDH6 | 0,596 | 1 |
| CDK12 | 0,621 | 1 |
| CDK16 | 1,917 | 1 |
| CDV3 | 0,486 | 1 |
| CENPB | 0,359 | 1 |
| CENPF | 2,739 | 1 |
| CEP170 | 0,253 | 1 |
| CFAP20 | 1,326 | 1 |
| CFDP1 | 0,126 | 1 |
| CFL1 | 0,639 | 1 |
| CFLAR | 0,327 | 1 |
| CHCHD1 | 0,661 | 1 |
| CHD1 | 0,667 | 1 |
| CHD3 | 0,294 | 1 |
| CHD8 | 0,972 | 1 |
| CHML | 1,443 | 1 |
| CHMP1B | 0,942 | 1 |
| CHN1 | 0,573 | 1 |
| CHP1 | 0,560 | 1 |
| CHPF | 2,269 | 0,546230341 |
| CHRAC1 | 1,118 | 1 |
| CHST2 | 0,681 | 1 |
| CIAPIN1 | 0,739 | 1 |
| CIB1 | 0,403 | 1 |
| CISD1 | 0,924 | 1 |
| CISD2 | 1,194 | 1 |
| CKAP2 | 2,471 | 1 |
| CKAP4 | 1,247 | 0,317608208 |
| CKS1B | 2,666 | 1 |
| CKS2 | 1,224 | 1 |
| CLDN5 | 1,700 | 1 |
| CLEC11A | 0,841 | 1 |
| CLEC2B | 1,336 | 1 |
| CLEC5A | 3,842 | 1 |
| CLIC1 | 1,195 | 0,017980824 |
| CLIC4 | 0,801 | 1 |
| CLINT1 | 0,718 | 1 |
| CLPP | 0,147 | 1 |
| CLPTM1 | 0,567 | 1 |
| CLTC | 0,612 | 0,005909573 |
| CMC2 | 0,531 | 1 |
| CMPK1 | 0,732 | 1 |
| CMTM3 | 1,034 | 1 |
| CMTM6 | 0,184 | 1 |
| CNDP2 | 0,409 | 1 |
| CNIH1 | 0,710 | 1 |
| CNIH4 | 0,941 | 1 |
| CNN3 | 0,228 | 1 |
| CNOT2 | 0,192 | 1 |
| CNP | 0,166 | 1 |
| COA4 | 0,782 | 1 |
| COL12A1 | 4,083 | 1 |
| COL18A1 | 2,628 | 9,16E-07 |
| COL1A1 | 5,433 | 4,70E-20 |
| COL1A2 | 2,542 | 9,70E-10 |
| COL3A1 | 4,078 | 2,49E-11 |
| COL4A1 | 3,839 | 5,41E-20 |
| COL4A2 | 3,774 | 1,92E-21 |
| COL5A1 | 2,575 | 0,00268713 |
| COL5A2 | 3,539 | 3,28E-05 |
| COL5A3 | 1,073 | 1 |
| COL6A1 | 2,493 | 1,61E-08 |
| COL6A2 | 1,847 | 0,000389593 |
| COL6A3 | 5,708 | 0,000146953 |
| COL7A1 | 2,284 | 1 |
| COLGALT1 | 1,699 | 1 |
| COPA | 0,157 | 1 |
| COPB1 | 0,854 | 1 |
| COPB2 | 0,740 | 1 |
| COPE | 0,119 | 1 |
| COPG1 | 0,604 | 1 |
| COPS2 | 0,795 | 1 |
| COPS9 | 0,172 | 1 |
| COPZ1 | 0,638 | 1 |
| CORO1C | 0,826 | 1 |
| COTL1 | 0,922 | 1 |
| COX4I1 | 0,142 | 1 |
| COX5A | 0,815 | 1 |
| CREB3L2 | 0,609 | 1 |
| CREG1 | 0,397 | 1 |
| CRELD2 | 0,933 | 1 |
| CREM | 1,214 | 1 |
| CRIP2 | 0,122 | 1 |
| CRISPLD2 | 0,417 | 1 |
| CRK | 0,358 | 1 |
| CRTAP | 0,282 | 1 |
| CSNK2B | 0,163 | 1 |
| CSPG4 | 1,622 | 1 |
| CSRP1 | 1,162 | 0,332289281 |
| CST3 | 0,772 | 0,010235551 |
| CSTB | 1,212 | 0,00045365 |
| CTBP1 | 0,251 | 1 |
| CTDNEP1 | 0,321 | 1 |
| CTGF | 1,697 | 0,000131562 |
| CTHRC1 | 3,112 | 1 |
| CTNNA1 | 0,924 | 1 |
| CTNNB1 | 1,013 | 0,001878297 |
| CTSA | 0,249 | 1 |
| CTSB | 1,069 | 0,007407166 |
| CTSC | 0,808 | 1 |
| CTSD | 1,603 | 2,20E-05 |
| CTSH | 0,657 | 1 |
| CTSK | 3,135 | 1 |
| CTSL | 1,933 | 5,26E-05 |
| CTSS | 0,922 | 0,077129718 |
| CTSZ | 0,854 | 1 |
| CUL3 | 0,530 | 1 |
| CUX1 | 0,281 | 1 |
| CXCL2 | 3,385 | 4,78E-06 |
| CXCL3 | 3,455 | 0,016227527 |
| CXCL8 | 2,794 | 3,81E-16 |
| CXXC5 | 0,568 | 1 |
| CYB5D1 | 3,206 | 1 |
| CYBA | 0,387 | 1 |
| CYBB | 1,003 | 1 |
| CYGB | 4,642 | 1 |
| CYR61 | 2,514 | 1,95E-06 |
| CYTH2 | 0,228 | 1 |
| CYTOR | 3,375 | 1,29E-15 |
| DAB2 | 1,374 | 0,22168995 |
| DAD1 | 0,266 | 1 |
| DAP | 1,428 | 1 |
| DAPK3 | 0,948 | 1 |
| DBN1 | 2,325 | 1 |
| DBNL | 0,191 | 1 |
| DCBLD2 | 1,493 | 1 |
| DCTN1 | 0,153 | 1 |
| DCTPP1 | 0,888 | 1 |
| DDOST | 0,428 | 1 |
| DDX1 | 0,120 | 1 |
| DDX21 | 0,326 | 1 |
| DDX39A | 1,083 | 1 |
| DDX3X | 0,324 | 1 |
| DDX49 | 2,874 | 1 |
| DDX52 | 0,736 | 1 |
| DECR1 | 0,787 | 1 |
| DEF8 | 0,599 | 1 |
| DEGS1 | 0,590 | 1 |
| DENND2A | 0,671 | 1 |
| DENR | 0,226 | 1 |
| DERL1 | 0,175 | 1 |
| DERL2 | 0,397 | 1 |
| DESI2 | 1,393 | 1 |
| DGKH | 0,632 | 1 |
| DGUOK | 0,308 | 1 |
| DHX15 | 0,813 | 1 |
| DHX29 | 0,671 | 1 |
| DHX9 | 0,157 | 1 |
| DLG1 | 0,635 | 1 |
| DLGAP4 | 1,586 | 1 |
| DMWD | 1,224 | 0,191859186 |
| DNAJA1 | 0,631 | 1 |
| DNAJA2 | 0,268 | 1 |
| DNAJB11 | 1,192 | 1 |
| DNAJB9 | 0,758 | 1 |
| DNAJC1 | 0,153 | 1 |
| DNAJC15 | 0,589 | 1 |
| DNAJC2 | 0,188 | 1 |
| DNAJC3 | 1,186 | 1 |
| DNMT1 | 1,548 | 1 |
| DNPH1 | 0,473 | 1 |
| DNTTIP2 | 0,940 | 1 |
| DOCK1 | 0,257 | 1 |
| DOCK10 | 0,139 | 1 |
| DOCK6 | 0,748 | 1 |
| DPM2 | 1,296 | 1 |
| DST | 0,290 | 1 |
| DSTN | 0,127 | 1 |
| DTYMK | 2,187 | 1 |
| DUSP2 | 1,496 | 1 |
| DUSP23 | 1,088 | 1 |
| DUSP5 | 1,853 | 1 |
| DUSP6 | 1,404 | 1 |
| DVL3 | 0,890 | 1 |
| DYNC1H1 | 0,233 | 1 |
| DYNC1LI1 | 0,817 | 1 |
| DYNC1LI2 | 0,443 | 1 |
| DYNLL1 | 0,275 | 1 |
| DYNLT1 | 0,526 | 1 |
| E2F3 | 1,995 | 1 |
| ECE1 | 0,508 | 1 |
| ECHDC1 | 1,159 | 1 |
| ECM1 | 0,947 | 1 |
| EDNRA | 2,027 | 0,45143624 |
| EDNRB | 0,313 | 1 |
| EEA1 | 0,467 | 1 |
| EEF1B2 | 0,481 | 1 |
| EEF2 | 0,476 | 1 |
| EFHD2 | 1,011 | 1 |
| EFTUD2 | 2,205 | 1 |
| EGFL7 | 0,769 | 1 |
| EHD1 | 1,086 | 1 |
| EIF2AK4 | 0,580 | 1 |
| EIF2S1 | 2,080 | 1 |
| EIF3A | 0,776 | 1 |
| EIF3B | 0,945 | 1 |
| EIF3H | 0,142 | 0,620235183 |
| EIF3I | 0,647 | 1 |
| EIF3J | 0,468 | 1 |
| EIF3M | 0,438 | 1 |
| EIF4A1 | 0,411 | 1 |
| EIF4A3 | 0,817 | 1 |
| EIF4E | 0,811 | 1 |
| EIF4E2 | 0,616 | 0,261254137 |
| EIF4EBP1 | 1,891 | 1 |
| EIF4G1 | 1,758 | 0,362402875 |
| EIF4G2 | 0,895 | 0,002582993 |
| EIF4G3 | 0,872 | 0,768284985 |
| EIF5A | 1,339 | 0,001693872 |
| EIF5B | 0,480 | 1 |
| ELAVL1 | 0,455 | 1 |
| ELK3 | 1,556 | 1 |
| ELOA | 1,111 | 1 |
| ELOC | 0,221 | 1 |
| ELOVL1 | 1,544 | 0,022176726 |
| ELOVL5 | 0,833 | 1 |
| EMC10 | 1,174 | 1 |
| EMC6 | 0,572 | 1 |
| EMC7 | 0,416 | 1 |
| EMILIN1 | 0,824 | 1 |
| EML4 | 0,693 | 1 |
| EMP1 | 4,771 | 9,08E-06 |
| EMP3 | 0,876 | 0,171648604 |
| ENAH | 0,666 | 1 |
| ENC1 | 3,383 | 0,126524707 |
| ENG | 1,802 | 0,014061048 |
| ENO1 | 2,262 | 5,27E-12 |
| ENOPH1 | 2,102 | 1 |
| ENPEP | 0,990 | 1 |
| ENTPD1 | 0,411 | 1 |
| ENY2 | 0,451 | 1 |
| EPB41L1 | 0,224 | 1 |
| EPRS | 1,363 | 1 |
| EPS15 | 0,588 | 1 |
| EPSTI1 | 0,663 | 1 |
| ERBIN | 0,974 | 1 |
| EREG | 3,496 | 1 |
| ERGIC1 | 0,143 | 1 |
| ERGIC2 | 0,309 | 1 |
| ERGIC3 | 0,520 | 1 |
| ERO1A | 1,589 | 1 |
| ERP29 | 0,181 | 1 |
| ERP44 | 0,542 | 1 |
| ERRFI1 | 2,663 | 1 |
| ESAM | 0,360 | 1 |
| ESF1 | 1,278 | 1 |
| ETF1 | 0,451 | 1 |
| ETS1 | 0,483 | 1 |
| EVA1B | 0,395 | 1 |
| EXOC5 | 0,184 | 1 |
| EXOSC6 | 0,535 | 1 |
| EZR | 0,246 | 1 |
| F2R | 3,794 | 0,002597401 |
| F3 | 2,459 | 0,077854168 |
| FABP5 | 1,772 | 0,000171711 |
| FAF2 | 0,846 | 1 |
| FAM107B | 0,594 | 1 |
| FAM114A1 | 1,346 | 1 |
| FAM120A | 0,414 | 1 |
| FAM13C | 0,595 | 1 |
| FAM177A1 | 0,292 | 1 |
| FAM207A | 0,894 | 1 |
| FAM20C | 0,697 | 1 |
| FAM234A | 1,141 | 1 |
| FAM49B | 0,628 | 1 |
| FAM50A | 0,201 | 1 |
| FAM91A1 | 0,457 | 1 |
| FAM98A | 1,148 | 1 |
| FARP1 | 0,801 | 1 |
| FAT1 | 1,123 | 0,037079142 |
| FBLIM1 | 1,886 | 0,510419115 |
| FBLN1 | 0,485 | 1 |
| FBN1 | 0,533 | 1 |
| FBXO32 | 0,292 | 1 |
| FCER1G | 1,826 | 0,000227477 |
| FCGRT | 0,198 | 1 |
| FCHO2 | 0,744 | 1 |
| FDPS | 0,569 | 1 |
| FDX1 | 0,189 | 0,415052692 |
| FH | 0,379 | 1 |
| FHL1 | 0,728 | 1 |
| FHL3 | 1,326 | 1 |
| FHOD1 | 1,448 | 1 |
| FIBP | 0,395 | 1 |
| FILIP1 | 0,938 | 1 |
| FILIP1L | 0,457 | 1 |
| FJX1 | 3,032 | 0,006550397 |
| FKBP10 | 1,643 | 0,923404555 |
| FKBP14 | 1,056 | 1 |
| FKBP1A | 1,626 | 0,004172542 |
| FKBP3 | 0,826 | 0,015764372 |
| FKBP7 | 1,639 | 1 |
| FKBP9 | 0,937 | 1 |
| FLII | 0,617 | 1 |
| FLOT1 | 0,603 | 1 |
| FLT1 | 2,908 | 0,002701664 |
| FN1 | 3,180 | 4,15E-16 |
| FNDC3A | 0,327 | 1 |
| FNDC3B | 2,121 | 0,186399051 |
| FNIP2 | 0,482 | 1 |
| FOSL2 | 0,896 | 1 |
| FOXP1 | 0,688 | 1 |
| FOXS1 | 0,855 | 1 |
| FRMD4B | 0,726 | 1 |
| FRMD8 | 1,697 | 1 |
| FSCN1 | 0,359 | 1 |
| FSTL1 | 2,457 | 0,000735742 |
| FUCA2 | 0,691 | 1 |
| FURIN | 2,247 | 1 |
| FUS | 0,336 | 1 |
| FXYD5 | 1,537 | 1 |
| FYN | 0,908 | 1 |
| G0S2 | 4,163 | 2,09E-13 |
| G3BP1 | 0,315 | 1 |
| G3BP2 | 0,872 | 1 |
| GADD45B | 0,214 | 1 |
| GALNT1 | 1,050 | 1 |
| GANAB | 0,520 | 1 |
| GAPDH | 0,815 | 1,10E-05 |
| GARS | 0,655 | 1 |
| GATAD2A | 1,433 | 1 |
| GBE1 | 1,066 | 1 |
| GBP2 | 0,410 | 1 |
| GCLM | 1,321 | 1 |
| GCSH | 0,671 | 1 |
| GDE1 | 0,807 | 1 |
| GDI2 | 1,128 | 1 |
| GFPT1 | 1,093 | 1 |
| GGCT | 1,236 | 1 |
| GIGYF2 | 0,475 | 1 |
| GINM1 | 0,303 | 1 |
| GJC1 | 0,125 | 1 |
| GLA | 1,818 | 1 |
| GLDN | 0,753 | 1 |
| GLG1 | 0,129 | 1 |
| GLIPR2 | 1,081 | 0,328327588 |
| GLRX | 0,102 | 1 |
| GLRX3 | 1,163 | 1 |
| GLS | 0,224 | 1 |
| GLUD1 | 0,387 | 1 |
| GLUL | 0,352 | 1 |
| GLYR1 | 0,909 | 1 |
| GMFG | 0,656 | 1 |
| GNA12 | 0,377 | 1 |
| GNAI2 | 0,804 | 1 |
| GNAI3 | 0,127 | 1 |
| GNB2 | 0,473 | 1 |
| GNG12 | 0,369 | 1 |
| GNG2 | 3,298 | 1 |
| GNG5 | 0,819 | 1 |
| GNL3 | 0,994 | 1 |
| GNS | 0,648 | 1 |
| GOLGA2 | 0,330 | 1 |
| GOLGA3 | 0,191 | 1 |
| GOLGA4 | 0,249 | 1 |
| GOLIM4 | 0,320 | 1 |
| GOLM1 | 1,109 | 1 |
| GOLPH3 | 0,622 | 1 |
| GOLT1B | 1,419 | 1 |
| GPATCH4 | 1,554 | 1 |
| GPC1 | 2,476 | 1 |
| GPCPD1 | 0,266 | 1 |
| GPI | 0,490 | 1 |
| GPM6B | 1,217 | 1 |
| GPNMB | 1,234 | 1 |
| GPR183 | 0,751 | 1 |
| GPX7 | 1,530 | 1 |
| GPX8 | 1,383 | 1 |
| GRB2 | 0,164 | 1 |
| GRN | 0,892 | 1 |
| GRPEL1 | 1,314 | 1 |
| GSK3B | 0,247 | 1 |
| GSPT1 | 0,856 | 1 |
| GSS | 2,207 | 1 |
| GSTK1 | 0,335 | 1 |
| GSTO1 | 1,354 | 0,033272541 |
| GTF2F1 | 0,688 | 1 |
| GTF3A | 0,208 | 1 |
| GTF3C6 | 0,457 | 1 |
| GTPBP4 | 0,652 | 1 |
| GUCY1A2 | 1,122 | 1 |
| GUCY1B1 | 0,331 | 1 |
| GUK1 | 0,550 | 1 |
| H2AFV | 0,399 | 1 |
| H2AFX | 1,278 | 1 |
| H2AFY | 0,710 | 1 |
| H2AFZ | 0,911 | 0,116224737 |
| HADHA | 0,273 | 1 |
| HADHB | 0,151 | 1 |
| HAMP | 1,019 | 1 |
| HARS | 1,300 | 1 |
| HBEGF | 1,304 | 1 |
| HDAC2 | 0,563 | 1 |
| HDGF | 0,714 | 1 |
| HDLBP | 0,613 | 1 |
| HEG1 | 1,061 | 1 |
| HELLS | 4,729 | 0,3570556 |
| HES1 | 0,777 | 1 |
| HEXA | 0,605 | 1 |
| HEXB | 0,182 | 1 |
| HEYL | 2,639 | 1 |
| HIF1A | 1,065 | 1 |
| HIGD2A | 0,170 | 1 |
| HIKESHI | 0,676 | 1 |
| HINT1 | 0,126 | 1 |
| HIP1 | 0,270 | 1 |
| HIPK2 | 0,703 | 1 |
| HIVEP3 | 1,279 | 1 |
| HK1 | 0,450 | 1 |
| HLA-A | 0,913 | 1 |
| HLA-DRB5 | 0,469 | 1 |
| HM13 | 0,842 | 1 |
| HMGCS1 | 1,393 | 1 |
| HMGN1 | 0,910 | 1 |
| HMGXB3 | 1,228 | 1 |
| HMOX1 | 3,040 | 1,08E-08 |
| HMOX2 | 0,783 | 1 |
| HNRNPA1 | 0,138 | 1 |
| HNRNPAB | 1,049 | 0,002320835 |
| HNRNPC | 0,491 | 1 |
| HNRNPF | 0,144 | 1 |
| HNRNPK | 0,242 | 1 |
| HNRNPM | 0,115 | 1 |
| HOMER3 | 1,021 | 1 |
| HPCAL1 | 2,407 | 1 |
| HSBP1 | 0,677 | 1 |
| HSD17B10 | 0,872 | 1 |
| HSD17B12 | 0,672 | 1 |
| HSP90AB1 | 0,481 | 1 |
| HSP90B1 | 1,301 | 0,00526236 |
| HSPA5 | 1,820 | 2,41E-05 |
| HSPA8 | 0,471 | 0,092244233 |
| HSPA9 | 0,348 | 1 |
| HSPD1 | 0,878 | 0,462139513 |
| HSPE1 | 0,530 | 1 |
| HSPG2 | 4,642 | 1 |
| HTATSF1 | 0,511 | 1 |
| HTRA1 | 0,113 | 1 |
| HYAL2 | 1,715 | 1 |
| HYOU1 | 0,902 | 1 |
| IARS | 2,120 | 1 |
| IARS2 | 0,282 | 1 |
| IBSP | 1,930 | 1 |
| ICAM1 | 1,117 | 1 |
| ICMT | 0,843 | 1 |
| ID1 | 1,551 | 1 |
| ID2 | 0,324 | 0,941979912 |
| ID3 | 0,658 | 1 |
| IDH1 | 1,464 | 1 |
| IDH2 | 0,702 | 1 |
| IER3 | 1,336 | 7,15E-05 |
| IER5L | 0,627 | 1 |
| IFNAR1 | 0,154 | 1 |
| IFNGR2 | 0,409 | 1 |
| IFT57 | 0,449 | 1 |
| IGFBP2 | 1,584 | 0,024811414 |
| IGFBP3 | 5,786 | 1 |
| IGFBP4 | 2,237 | 4,50E-07 |
| IKBIP | 1,212 | 0,559824695 |
| IL1A | 3,376 | 1 |
| IL1B | 1,894 | 1,47E-07 |
| IL1R1 | 0,274 | 1 |
| IL1RN | 1,851 | 0,599047679 |
| IL32 | 4,119 | 0,006142921 |
| IL6 | 2,537 | 1 |
| ILF2 | 0,428 | 1 |
| IMP4 | 0,905 | 1 |
| IMPAD1 | 0,634 | 1 |
| IMPDH2 | 0,469 | 1 |
| INAFM1 | 0,859 | 0,105279134 |
| INHBA | 2,146 | 1 |
| INSIG1 | 1,169 | 1 |
| INSR | 1,738 | 1 |
| IPO7 | 0,776 | 1 |
| IQGAP1 | 1,472 | 1 |
| IQSEC1 | 1,039 | 1 |
| IRAK1 | 1,490 | 1 |
| IRF1 | 1,234 | 1 |
| ISG15 | 0,542 | 1 |
| ISG20 | 2,946 | 1 |
| ISG20L2 | 0,682 | 1 |
| ISLR | 0,929 | 1 |
| ISOC2 | 1,844 | 1 |
| ITGA1 | 1,716 | 4,21E-05 |
| ITGA11 | 1,222 | 1 |
| ITGA4 | 3,010 | 1 |
| ITGA5 | 2,938 | 0,132008167 |
| ITGA6 | 1,560 | 1 |
| ITGAE | 0,412 | 1 |
| ITGAV | 1,049 | 1 |
| ITGB1 | 1,198 | 5,93E-05 |
| ITPA | 0,507 | 1 |
| ITPRIPL2 | 0,763 | 1 |
| IVNS1ABP | 0,720 | 1 |
| JMJD1C | 0,954 | 1 |
| JOSD2 | 0,425 | 1 |
| JPT1 | 0,779 | 1 |
| JTB | 1,217 | 1 |
| KALRN | 1,126 | 1 |
| KCMF1 | 0,881 | 1 |
| KCNJ8 | 0,810 | 1 |
| KCNQ1OT1 | 2,155 | 0,067655317 |
| KCTD20 | 1,348 | 1 |
| KDELR1 | 0,939 | 1 |
| KDELR2 | 1,273 | 1 |
| KDELR3 | 1,504 | 1 |
| KDM5B | 1,158 | 1 |
| KDM6B | 1,027 | 0,98343921 |
| KHDRBS1 | 0,662 | 1 |
| KIAA2013 | 0,302 | 1 |
| KIF1B | 1,109 | 1 |
| KLF10 | 0,325 | 1 |
| KLF4 | 0,239 | 1 |
| KLHDC3 | 1,131 | 1 |
| KLHL5 | 0,254 | 1 |
| KNOP1 | 0,280 | 1 |
| KPNA2 | 0,707 | 1 |
| KPNB1 | 0,761 | 1 |
| KRT10 | 0,805 | 0,11824659 |
| LACTB | 0,673 | 1 |
| LAMA2 | 0,158 | 1 |
| LAMA4 | 2,363 | 3,91E-07 |
| LAMB1 | 4,334 | 1,34E-05 |
| LAMC1 | 1,331 | 0,002410604 |
| LAMC3 | 1,518 | 1 |
| LAMP2 | 0,873 | 1 |
| LAMTOR1 | 0,726 | 1 |
| LAMTOR2 | 1,492 | 0,142925712 |
| LAMTOR5 | 0,146 | 1 |
| LAP3 | 0,427 | 1 |
| LAPTM4B | 1,231 | 1 |
| LAPTM5 | 0,767 | 1 |
| LARP1 | 1,628 | 1 |
| LARS | 1,040 | 1 |
| LASP1 | 0,484 | 1 |
| LAYN | 2,828 | 1 |
| LBR | 0,635 | 1 |
| LCP1 | 1,042 | 1 |
| LCP2 | 0,708 | 1 |
| LDHA | 1,626 | 1,67E-08 |
| LDHB | 0,191 | 1 |
| LDLR | 1,860 | 1 |
| LDLRAD3 | 2,340 | 1 |
| LEPROTL1 | 0,833 | 1 |
| LGALS1 | 0,638 | 0,003694828 |
| LGALS3 | 1,311 | 0,000892154 |
| LGALS3BP | 0,803 | 1 |
| LGMN | 0,229 | 1 |
| LHFPL2 | 0,196 | 1 |
| LIMA1 | 1,250 | 0,430209791 |
| LIMS1 | 1,252 | 0,025639352 |
| LINGO1 | 0,952 | 1 |
| LIPA | 0,596 | 0,726267163 |
| LITAF | 1,299 | 1 |
| LLPH | 0,679 | 1 |
| LMAN1 | 0,873 | 0,810158961 |
| LMAN2 | 0,654 | 1 |
| LMNA | 0,915 | 0,005334553 |
| LONP1 | 1,407 | 1 |
| LOXL2 | 4,621 | 4,35E-07 |
| LPGAT1 | 0,567 | 1 |
| LPL | 0,132 | 1 |
| LRRC32 | 1,347 | 1 |
| LRRC59 | 1,823 | 1 |
| LRRC75A | 1,613 | 1 |
| LRRC8A | 0,596 | 1 |
| LRRFIP1 | 1,003 | 0,001514049 |
| LRRFIP2 | 1,362 | 1 |
| LSM12 | 0,602 | 1 |
| LSM4 | 0,711 | 1 |
| LSP1 | 2,460 | 1 |
| LTBP4 | 4,385 | 0,914470403 |
| LTBR | 1,341 | 1 |
| LUCAT1 | 3,238 | 1 |
| LUM | 2,033 | 0,002834857 |
| LUZP1 | 0,242 | 1 |
| LYPLA1 | 0,907 | 1 |
| LYZ | 3,395 | 1,22E-13 |
| M6PR | 0,232 | 1 |
| MACF1 | 0,826 | 1 |
| MACO1 | 0,480 | 1 |
| MAFB | 0,643 | 1 |
| MAFF | 1,210 | 1 |
| MAGED1 | 0,674 | 1 |
| MAN1A2 | 0,650 | 1 |
| MANF | 0,992 | 0,501069031 |
| MAP1B | 0,650 | 1 |
| MAP1LC3B | 0,757 | 0,290126183 |
| MAP2K2 | 0,294 | 1 |
| MAP2K3 | 1,237 | 1 |
| MAP3K2 | 0,485 | 1 |
| MAP3K7CL | 1,545 | 1 |
| MAP3K8 | 1,114 | 1 |
| MAP4 | 0,781 | 0,15272398 |
| MAP4K4 | 0,428 | 1 |
| MAP4K5 | 0,711 | 1 |
| MAPK1 | 0,224 | 1 |
| MAPK1IP1L | 0,346 | 1 |
| MAPK6 | 2,359 | 0,080357797 |
| MAPKAP1 | 1,085 | 1 |
| MAPKAPK2 | 1,685 | 1 |
| MAPRE1 | 0,657 | 1 |
| MARCH2 | 0,280 | 1 |
| MARCKS | 0,789 | 0,310160733 |
| MARCKSL1 | 1,729 | 0,00090177 |
| MAST4 | 0,565 | 1 |
| MAZ | 0,590 | 1 |
| MBTPS1 | 0,693 | 1 |
| MCAM | 0,921 | 0,306845119 |
| MCM4 | 3,845 | 1 |
| MCRIP1 | 0,924 | 1 |
| MCTS1 | 1,031 | 1 |
| MDFI | 2,307 | 1 |
| MDFIC | 1,920 | 1 |
| MDK | 2,183 | 1 |
| MDM2 | 0,159 | 1 |
| ME2 | 0,852 | 1 |
| MED8 | 1,009 | 1 |
| MEG3 | 2,794 | 0,000281191 |
| MESD | 0,478 | 1 |
| MEST | 1,720 | 1 |
| METRN | 1,169 | 0,442543165 |
| METRNL | 1,215 | 1 |
| MFSD12 | 1,642 | 1 |
| MGAT1 | 0,228 | 1 |
| MGAT4B | 1,173 | 1 |
| MGP | 2,264 | 0,000103255 |
| MICAL2 | 3,809 | 1 |
| MICALL2 | 1,670 | 1 |
| MICU1 | 0,186 | 1 |
| MIDN | 0,371 | 1 |
| MIEN1 | 0,285 | 1 |
| MIF | 0,402 | 1 |
| MIR22HG | 0,958 | 1 |
| MIR4435-2HG | 3,546 | 9,21E-14 |
| MKI67 | 7,491 | 0,054615932 |
| MLEC | 0,983 | 1 |
| MLLT11 | 2,294 | 0,102613309 |
| MMP11 | 6,105 | 1 |
| MMP14 | 2,574 | 1 |
| MMP16 | 3,746 | 1 |
| MORF4L2 | 0,938 | 1 |
| MPDU1 | 1,053 | 1 |
| MRPL12 | 0,432 | 1 |
| MRPL14 | 1,361 | 1 |
| MRPL17 | 0,354 | 1 |
| MRPL32 | 0,659 | 1 |
| MRPL36 | 1,131 | 1 |
| MRPL4 | 0,777 | 1 |
| MRPL41 | 0,194 | 1 |
| MRPL42 | 0,193 | 1 |
| MRPL51 | 0,207 | 1 |
| MRPL52 | 0,130 | 1 |
| MRPS11 | 1,070 | 1 |
| MRPS12 | 0,820 | 1 |
| MRPS15 | 0,451 | 1 |
| MRTO4 | 2,083 | 1 |
| MRVI1 | 0,312 | 1 |
| MS4A6A | 0,830 | 1 |
| MSANTD3 | 1,848 | 0,581192591 |
| MSL3 | 0,311 | 1 |
| MSN | 0,900 | 1 |
| MSR1 | 1,179 | 1 |
| MT-ND5 | 0,214 | 1 |
| MT1X | 0,725 | 1 |
| MTCH2 | 0,752 | 1 |
| MTDH | 0,335 | 1 |
| MTHFD2 | 0,465 | 1 |
| MTIF3 | 0,409 | 1 |
| MTMR6 | 0,709 | 1 |
| MTPN | 0,612 | 1 |
| MTUS1 | 0,504 | 1 |
| MVP | 1,516 | 1 |
| MXRA5 | 3,810 | 1 |
| MXRA8 | 0,694 | 1 |
| MYADM | 0,381 | 1 |
| MYC | 0,856 | 1 |
| MYDGF | 0,888 | 1 |
| MYH9 | 0,483 | 1 |
| MYL12A | 0,597 | 1 |
| MYL6 | 0,305 | 1 |
| MYL6B | 0,550 | 1 |
| MYO10 | 3,600 | 1 |
| MYO1B | 0,238 | 1 |
| MYO1E | 0,604 | 1 |
| MYOF | 0,387 | 1 |
| N4BP1 | 0,870 | 1 |
| NAA50 | 0,912 | 1 |
| NAMPT | 0,645 | 1 |
| NAP1L1 | 0,407 | 1 |
| NAP1L4 | 0,213 | 1 |
| NARS | 0,196 | 1 |
| NASP | 0,413 | 1 |
| NAV1 | 1,265 | 1 |
| NBN | 0,713 | 1 |
| NCBP2 | 0,158 | 1 |
| NCK2 | 0,496 | 1 |
| NCL | 0,841 | 1 |
| NCLN | 1,710 | 1 |
| NCOA7 | 0,856 | 1 |
| NDUFA11 | 0,374 | 1 |
| NDUFA4 | 0,703 | 1 |
| NDUFA6 | 0,217 | 1 |
| NDUFAB1 | 0,339 | 1 |
| NDUFAF8 | 0,863 | 1 |
| NDUFB11 | 0,254 | 1 |
| NDUFB2 | 0,107 | 1 |
| NDUFB5 | 0,589 | 1 |
| NDUFS6 | 0,229 | 1 |
| NDUFV2 | 0,645 | 1 |
| NDUFV3 | 0,661 | 1 |
| NEAT1 | 0,331 | 1 |
| NECAP2 | 0,781 | 1 |
| NECTIN2 | 1,084 | 1 |
| NENF | 0,434 | 1 |
| NES | 1,792 | 0,001502515 |
| NEU1 | 0,530 | 1 |
| NF1 | 0,442 | 1 |
| NFE2L2 | 0,413 | 1 |
| NFIB | 0,971 | 1 |
| NFIC | 0,130 | 1 |
| NFIL3 | 0,295 | 1 |
| NFKB1 | 0,368 | 1 |
| NFKBIA | 0,548 | 1 |
| NFKBIZ | 0,700 | 1 |
| NHP2 | 0,862 | 1 |
| NID1 | 2,242 | 0,007309605 |
| NID2 | 3,107 | 0,02041865 |
| NIFK | 0,890 | 1 |
| NIN | 0,944 | 1 |
| NINJ1 | 0,560 | 1 |
| NIPBL | 0,209 | 1 |
| NIT2 | 1,411 | 1 |
| NMD3 | 0,474 | 1 |
| NME1 | 1,950 | 0,000914254 |
| NME4 | 0,653 | 1 |
| NNMT | 2,851 | 1 |
| NODAL | 2,809 | 1 |
| NOLC1 | 1,018 | 1 |
| NONO | 0,506 | 1 |
| NOP10 | 0,754 | 1 |
| NOP56 | 0,346 | 1 |
| NPC2 | 0,726 | 1 |
| NPM1 | 0,858 | 1 |
| NQO1 | 1,259 | 1 |
| NR4A2 | 0,387 | 0,439625977 |
| NRARP | 1,231 | 1 |
| NRAS | 0,669 | 1 |
| NRBP1 | 0,652 | 1 |
| NREP | 4,679 | 0,255664058 |
| NRIP1 | 0,170 | 1 |
| NRP1 | 3,053 | 0,000971178 |
| NRP2 | 0,702 | 1 |
| NSD3 | 0,266 | 1 |
| NT5DC2 | 0,298 | 1 |
| NTAN1 | 0,493 | 1 |
| NTM | 1,537 | 0,755367505 |
| NTMT1 | 0,925 | 1 |
| NTPCR | 0,848 | 1 |
| NUBP2 | 0,848 | 1 |
| NUCB1 | 0,403 | 1 |
| NUCB2 | 0,670 | 1 |
| NUDT5 | 1,686 | 1 |
| NUDT9 | 1,202 | 1 |
| NUFIP2 | 0,418 | 1 |
| NUTF2 | 0,867 | 1 |
| OAZ1 | 0,416 | 0,018328412 |
| OAZ2 | 0,248 | 1 |
| ODF2L | 0,806 | 1 |
| OLFML2A | 1,759 | 1 |
| OLFML2B | 1,392 | 1 |
| OLFML3 | 1,329 | 1 |
| ORAI2 | 1,169 | 1 |
| OS9 | 0,897 | 0,183362838 |
| OSMR | 3,871 | 1 |
| OST4 | 0,387 | 1 |
| OSTC | 1,338 | 1 |
| OSTF1 | 0,411 | 1 |
| OXSR1 | 1,252 | 1 |
| P3H1 | 0,935 | 1 |
| P3H4 | 3,524 | 1 |
| P4HA1 | 1,317 | 1 |
| P4HB | 2,238 | 3,81E-08 |
| PA2G4 | 0,474 | 1 |
| PABPC1 | 0,536 | 1 |
| PAFAH1B2 | 0,689 | 1 |
| PAG1 | 0,231 | 1 |
| PAK2 | 0,342 | 1 |
| PAM | 1,082 | 1 |
| PAMR1 | 1,705 | 1 |
| PAPSS1 | 0,381 | 1 |
| PAPSS2 | 1,200 | 1 |
| PARP1 | 0,531 | 1 |
| PARVA | 0,948 | 1 |
| PARVB | 0,426 | 1 |
| PCBP1 | 0,161 | 1 |
| PCDH18 | 0,467 | 1 |
| PCLAF | 6,665 | 1 |
| PCM1 | 0,232 | 1 |
| PCNA | 1,177 | 1 |
| PCOLCE | 2,709 | 5,19E-05 |
| PCYT1A | 0,387 | 1 |
| PDCD5 | 0,179 | 1 |
| PDCD6IP | 0,259 | 1 |
| PDCL | 0,789 | 1 |
| PDE4B | 1,120 | 1 |
| PDE8A | 0,391 | 1 |
| PDGFA | 1,156 | 1 |
| PDIA3 | 1,382 | 0,003412395 |
| PDIA4 | 1,699 | 0,0981301 |
| PDIA6 | 1,770 | 0,244897765 |
| PDLIM1 | 1,596 | 0,098754688 |
| PDLIM3 | 1,606 | 1 |
| PDLIM5 | 0,944 | 1 |
| PDS5A | 0,756 | 1 |
| PDS5B | 0,322 | 1 |
| PDXK | 0,528 | 1 |
| PDZD11 | 0,981 | 1 |
| PEA15 | 0,151 | 1 |
| PEAK1 | 0,569 | 1 |
| PECAM1 | 1,926 | 1 |
| PELO | 0,305 | 1 |
| PFKL | 1,001 | 1 |
| PFKP | 1,817 | 1 |
| PFN1 | 1,511 | 1,07E-07 |
| PGAM1 | 0,538 | 0,327101734 |
| PGD | 1,600 | 1 |
| PGF | 0,422 | 1 |
| PGK1 | 0,896 | 0,817429897 |
| PGLS | 0,509 | 1 |
| PGM2L1 | 1,194 | 1 |
| PHACTR1 | 0,530 | 1 |
| PHB | 0,506 | 1 |
| PHC2 | 1,035 | 1 |
| PHF20L1 | 0,107 | 1 |
| PHKG1 | 1,540 | 1 |
| PHLDA1 | 1,092 | 0,001031739 |
| PHLDA2 | 2,625 | 0,005569199 |
| PHLDB1 | 0,680 | 1 |
| PIEZO2 | 2,304 | 0,661533913 |
| PIM1 | 4,426 | 0,064930198 |
| PIM3 | 0,506 | 1 |
| PIP5K1C | 2,075 | 1 |
| PITPNA | 1,342 | 1 |
| PKM | 1,032 | 0,098353576 |
| PLAT | 2,295 | 1 |
| PLAU | 2,446 | 0,386807159 |
| PLAUR | 1,619 | 0,609744679 |
| PLD3 | 0,264 | 1 |
| PLEC | 0,387 | 1 |
| PLEKHA1 | 0,993 | 1 |
| PLEKHB2 | 2,261 | 0,356498825 |
| PLEKHG1 | 2,336 | 1 |
| PLEKHG2 | 0,777 | 1 |
| PLEKHM2 | 1,616 | 0,006576867 |
| PLIN2 | 2,445 | 0,000863888 |
| PLIN3 | 1,338 | 0,578214828 |
| PLK2 | 1,609 | 1 |
| PLOD1 | 0,659 | 1 |
| PLOD2 | 2,535 | 1 |
| PLOD3 | 2,145 | 1 |
| PLP2 | 1,574 | 1 |
| PLS3 | 0,196 | 1 |
| PLTP | 0,752 | 1 |
| PLXDC1 | 0,320 | 1 |
| PLXND1 | 1,482 | 0,698563215 |
| PMAIP1 | 0,701 | 1 |
| PML | 2,254 | 1 |
| PMP22 | 0,606 | 1 |
| PNP | 3,703 | 1 |
| PNPLA8 | 0,448 | 1 |
| POLR1D | 0,154 | 1 |
| POLR2B | 0,402 | 1 |
| POLR2L | 0,190 | 1 |
| POLR3D | 1,998 | 1 |
| POMP | 0,655 | 0,170499424 |
| PON2 | 0,923 | 1 |
| POP7 | 0,377 | 1 |
| POR | 0,584 | 1 |
| PPA1 | 0,674 | 1 |
| PPCS | 0,602 | 1 |
| PPFIA1 | 1,329 | 0,22418603 |
| PPFIBP1 | 0,383 | 1 |
| PPIA | 0,735 | 0,012849803 |
| PPIB | 1,120 | 9,83E-07 |
| PPIC | 2,032 | 1 |
| PPIF | 1,393 | 1 |
| PPM1G | 0,600 | 0,026759344 |
| PPP1CA | 0,582 | 1 |
| PPP1CC | 0,336 | 1 |
| PPP1R14B | 0,981 | 1 |
| PPP1R15A | 0,548 | 1 |
| PPP1R15B | 0,570 | 1 |
| PPP1R18 | 1,007 | 1 |
| PPP1R2 | 0,486 | 1 |
| PPP2CA | 0,609 | 1 |
| PPP2R1A | 0,267 | 1 |
| PPP2R2A | 0,761 | 1 |
| PPP2R5C | 0,127 | 1 |
| PPP3CA | 0,328 | 1 |
| PPP4C | 0,397 | 1 |
| PPP6C | 0,441 | 1 |
| PPP6R1 | 0,453 | 1 |
| PPT1 | 0,429 | 1 |
| PRCC | 0,778 | 1 |
| PRDM1 | 1,891 | 1 |
| PRDX1 | 0,533 | 0,176565118 |
| PRDX3 | 0,523 | 1 |
| PRDX4 | 0,339 | 1 |
| PRELID1 | 0,672 | 1 |
| PRF1 | 2,426 | 1 |
| PRKAR2A | 0,564 | 1 |
| PRKCB | 1,039 | 1 |
| PRKCSH | 0,383 | 1 |
| PRKDC | 0,548 | 1 |
| PRKG1 | 0,659 | 1 |
| PRNP | 1,082 | 1 |
| PRPF40A | 0,194 | 1 |
| PRPF4B | 0,366 | 1 |
| PRR14L | 0,339 | 1 |
| PRRC1 | 1,515 | 1 |
| PRRC2C | 0,217 | 1 |
| PRSS23 | 4,379 | 0,001168162 |
| PSAP | 1,070 | 0,004194446 |
| PSMA3 | 0,970 | 1 |
| PSMA4 | 0,888 | 1 |
| PSMA5 | 0,409 | 1 |
| PSMB3 | 0,598 | 1 |
| PSMB6 | 0,321 | 1 |
| PSMB8 | 0,213 | 1 |
| PSMC2 | 0,848 | 1 |
| PSMD1 | 0,991 | 1 |
| PSMD11 | 0,406 | 1 |
| PSMD14 | 1,120 | 1 |
| PSMD2 | 0,716 | 1 |
| PSMD7 | 0,188 | 1 |
| PSMD8 | 0,173 | 1 |
| PSME2 | 0,883 | 1 |
| PSME4 | 0,342 | 1 |
| PSMG3 | 0,626 | 1 |
| PTBP1 | 0,212 | 1 |
| PTBP3 | 0,618 | 1 |
| PTGFRN | 4,136 | 1 |
| PTGIR | 3,735 | 0,3570556 |
| PTGS2 | 0,590 | 1 |
| PTMA | 0,611 | 0,001245104 |
| PTP4A1 | 0,702 | 1 |
| PTP4A2 | 0,314 | 1 |
| PTP4A3 | 3,114 | 0,000952638 |
| PTPN1 | 0,706 | 1 |
| PTPN12 | 0,109 | 1 |
| PTPRA | 0,706 | 1 |
| PTPRE | 0,339 | 1 |
| PTRHD1 | 0,660 | 1 |
| PTTG1IP | 0,380 | 1 |
| PURB | 0,354 | 1 |
| PXDC1 | 0,983 | 1 |
| PXDN | 2,999 | 2,94E-05 |
| PXN | 3,036 | 1 |
| PYCARD | 0,466 | 1 |
| QARS | 1,058 | 1 |
| QPCT | 2,490 | 1 |
| RAB10 | 0,771 | 1 |
| RAB11A | 0,233 | 1 |
| RAB13 | 1,645 | 0,065419183 |
| RAB18 | 0,346 | 1 |
| RAB1B | 0,347 | 1 |
| RAB22A | 0,678 | 1 |
| RAB2A | 0,125 | 1 |
| RAB31 | 0,669 | 1 |
| RAB32 | 0,506 | 1 |
| RAB4A | 0,328 | 1 |
| RAB5C | 0,712 | 1 |
| RAB5IF | 1,648 | 1 |
| RAB6A | 0,351 | 1 |
| RAB7A | 0,394 | 1 |
| RABEP1 | 0,472 | 1 |
| RABL6 | 0,256 | 1 |
| RACK1 | 0,211 | 1 |
| RAD21 | 0,334 | 1 |
| RAD23B | 0,692 | 1 |
| RAI14 | 2,820 | 0,001111967 |
| RALA | 1,457 | 0,09579604 |
| RALB | 1,766 | 0,17565105 |
| RAN | 0,602 | 1 |
| RANBP1 | 0,206 | 1 |
| RANBP2 | 0,556 | 1 |
| RAP1B | 0,544 | 1 |
| RAP2B | 1,549 | 1 |
| RAPGEF2 | 0,636 | 1 |
| RARS | 0,390 | 1 |
| RASGEF1B | 0,789 | 1 |
| RASL12 | 0,148 | 1 |
| RBBP4 | 0,473 | 1 |
| RBBP6 | 0,823 | 1 |
| RBM17 | 0,177 | 1 |
| RBM25 | 0,374 | 1 |
| RBM3 | 1,492 | 1 |
| RBMS1 | 0,240 | 1 |
| RCAN1 | 1,543 | 0,193191031 |
| RCC2 | 0,195 | 1 |
| RCN1 | 1,406 | 0,66824419 |
| RCN2 | 0,545 | 1 |
| RCN3 | 1,640 | 1 |
| RDX | 0,525 | 1 |
| RECQL | 1,472 | 1 |
| REEP3 | 1,269 | 1 |
| RER1 | 0,567 | 1 |
| REV3L | 0,312 | 1 |
| REXO2 | 0,270 | 1 |
| RFLNB | 2,490 | 1 |
| RFTN1 | 0,279 | 1 |
| RGCC | 0,212 | 1 |
| RGS3 | 3,814 | 1 |
| RHBDD2 | 0,863 | 1 |
| RHOA | 0,206 | 1 |
| RHOC | 0,241 | 1 |
| RHOG | 0,347 | 1 |
| RIF1 | 0,360 | 1 |
| RILPL2 | 0,184 | 1 |
| RIOK3 | 0,590 | 1 |
| RIT1 | 1,033 | 1 |
| RLF | 1,602 | 1 |
| RMDN1 | 1,910 | 1 |
| RMDN3 | 0,675 | 1 |
| RNASE1 | 1,868 | 1 |
| RNF10 | 0,534 | 1 |
| RNF11 | 0,170 | 1 |
| RNF145 | 1,049 | 1 |
| RNF19A | 0,210 | 1 |
| RNF24 | 1,740 | 0,079496245 |
| RNMT | 0,603 | 1 |
| RNPEP | 1,153 | 1 |
| RP2 | 1,085 | 1 |
| RPA3 | 0,661 | 1 |
| RPF2 | 1,580 | 1 |
| RPL10A | 0,230 | 1 |
| RPL12 | 0,213 | 1 |
| RPL18A | 0,151 | 1 |
| RPL22L1 | 1,591 | 0,15007715 |
| RPL23 | 0,226 | 1 |
| RPL26L1 | 0,616 | 1 |
| RPL27 | 0,430 | 1 |
| RPL28 | 0,600 | 1 |
| RPL29 | 0,143 | 1 |
| RPL39 | 0,192 | 1 |
| RPL4 | 0,373 | 1 |
| RPL8 | 0,141 | 1 |
| RPLP0 | 0,928 | 7,30E-05 |
| RPN1 | 0,683 | 1 |
| RPN2 | 0,639 | 1 |
| RPS17 | 0,188 | 1 |
| RPS2 | 0,508 | 1 |
| RPS20 | 0,177 | 1 |
| RPS26 | 0,309 | 1 |
| RPS27L | 0,323 | 1 |
| RPSA | 0,435 | 1 |
| RRBP1 | 1,672 | 1 |
| RRP36 | 0,904 | 1 |
| RSL1D1 | 0,199 | 1 |
| RSRC1 | 1,101 | 1 |
| RSRC2 | 0,148 | 1 |
| RSU1 | 0,681 | 1 |
| RTN3 | 0,639 | 1 |
| RUNX1 | 0,448 | 1 |
| RUVBL1 | 2,222 | 1 |
| RYBP | 0,333 | 1 |
| S100A10 | 3,161 | 1,45E-09 |
| S100A11 | 1,384 | 2,81E-07 |
| S100A4 | 2,422 | 0,000181736 |
| S100A8 | 2,493 | 4,44E-06 |
| S100A9 | 2,153 | 1,24E-05 |
| S1PR3 | 1,067 | 1 |
| SAE1 | 0,791 | 1 |
| SAMHD1 | 0,727 | 1 |
| SAP18 | 0,531 | 1 |
| SAP30 | 0,601 | 1 |
| SAR1A | 0,607 | 1 |
| SAR1B | 0,471 | 1 |
| SCAMP3 | 1,816 | 1 |
| SCAND1 | 0,458 | 1 |
| SCARB2 | 0,377 | 1 |
| SCG2 | 5,138 | 2,07E-05 |
| SCG5 | 1,888 | 1 |
| SCP2 | 0,434 | 1 |
| SCPEP1 | 0,206 | 1 |
| SDC2 | 1,634 | 0,240243314 |
| SDCBP | 0,825 | 1 |
| SDF2L1 | 1,649 | 1 |
| SDF4 | 0,631 | 1 |
| SDHC | 0,304 | 1 |
| SEC11A | 0,218 | 1 |
| SEC13 | 0,174 | 1 |
| SEC22B | 1,017 | 1 |
| SEC23A | 1,774 | 1 |
| SEC24A | 1,958 | 1 |
| SEC24D | 1,738 | 1 |
| SEC31A | 0,984 | 1 |
| SEC61A1 | 1,148 | 1 |
| SEC61B | 0,787 | 0,101764331 |
| SEC61G | 0,251 | 1 |
| SEL1L | 0,393 | 1 |
| SELENOF | 0,220 | 1 |
| SELENOT | 0,380 | 1 |
| SEMA5A | 1,185 | 1 |
| SEPHS2 | 0,710 | 1 |
| SEPT10 | 0,559 | 1 |
| SEPT11 | 1,213 | 0,03816619 |
| SEPT2 | 0,352 | 0,028526995 |
| SEPT9 | 0,471 | 0,072538234 |
| SERBP1 | 0,590 | 0,021054506 |
| SERF2 | 1,129 | 7,51E-12 |
| SERPINB1 | 0,700 | 1 |
| SERPINB2 | 7,662 | 1 |
| SERPINE1 | 0,144 | 1 |
| SERPINH1 | 2,464 | 0,000107052 |
| SET | 0,797 | 0,00090584 |
| SF3A3 | 0,131 | 1 |
| SF3B2 | 0,473 | 1 |
| SF3B5 | 0,353 | 1 |
| SFPQ | 0,396 | 1 |
| SFT2D1 | 0,567 | 1 |
| SH3BGRL3 | 1,360 | 1,01E-06 |
| SH3GLB1 | 0,456 | 1 |
| SH3PXD2A | 1,658 | 0,686247936 |
| SHC1 | 3,260 | 0,465266467 |
| SHMT2 | 1,076 | 1 |
| SLBP | 0,825 | 1 |
| SLC12A2 | 0,389 | 1 |
| SLC16A1 | 1,976 | 1 |
| SLC16A10 | 3,112 | 1 |
| SLC16A3 | 0,904 | 1 |
| SLC20A1 | 1,187 | 1 |
| SLC25A24 | 1,049 | 1 |
| SLC25A3 | 0,738 | 0,015579166 |
| SLC25A5 | 1,068 | 0,005159632 |
| SLC26A2 | 1,714 | 1 |
| SLC29A1 | 0,646 | 1 |
| SLC30A7 | 2,210 | 1 |
| SLC31A1 | 1,480 | 0,6631969 |
| SLC35B1 | 2,127 | 1 |
| SLC35E1 | 0,577 | 1 |
| SLC39A1 | 0,571 | 1 |
| SLC39A7 | 0,207 | 1 |
| SLC39A8 | 4,215 | 1 |
| SLC3A2 | 0,763 | 1 |
| SLC40A1 | 0,841 | 1 |
| SLC52A2 | 1,665 | 1 |
| SLC7A5 | 2,208 | 1 |
| SLFN5 | 1,616 | 1 |
| SLTM | 0,205 | 1 |
| SMAP1 | 0,723 | 1 |
| SMARCA1 | 0,668 | 1 |
| SMARCA4 | 0,571 | 0,231325151 |
| SMARCAD1 | 1,120 | 1 |
| SMARCB1 | 0,467 | 1 |
| SMARCC1 | 0,953 | 1 |
| SMC1A | 0,875 | 1 |
| SMC2 | 2,876 | 1 |
| SMC4 | 0,863 | 1 |
| SMC5 | 1,059 | 1 |
| SMC6 | 0,973 | 1 |
| SMCHD1 | 0,938 | 1 |
| SMIM10L1 | 0,268 | 1 |
| SMIM30 | 0,375 | 1 |
| SNAI2 | 2,037 | 1 |
| SNAP29 | 0,601 | 1 |
| SND1 | 1,175 | 1 |
| SNRPB | 0,429 | 1 |
| SNRPB2 | 0,451 | 1 |
| SNRPC | 0,251 | 1 |
| SNRPD1 | 0,829 | 1 |
| SNRPE | 0,388 | 1 |
| SNTB2 | 2,055 | 1 |
| SNU13 | 0,364 | 1 |
| SNX10 | 1,195 | 1 |
| SNX17 | 0,336 | 1 |
| SNX3 | 0,299 | 1 |
| SNX4 | 0,826 | 1 |
| SNX6 | 1,038 | 1 |
| SOCS2 | 2,889 | 1 |
| SOCS3 | 0,466 | 1 |
| SOCS4 | 0,955 | 1 |
| SOD2 | 1,992 | 0,000606705 |
| SOGA1 | 1,145 | 1 |
| SOX4 | 1,349 | 0,043061627 |
| SPAG9 | 0,507 | 1 |
| SPARC | 1,808 | 1,02E-10 |
| SPARCL1 | 0,416 | 1 |
| SPCS1 | 0,417 | 1 |
| SPCS3 | 0,851 | 1 |
| SPEN | 0,462 | 1 |
| SPG21 | 1,238 | 1 |
| SPHK1 | 1,292 | 1 |
| SPIN1 | 0,302 | 1 |
| SPNS1 | 0,601 | 1 |
| SPON2 | 4,513 | 3,23E-08 |
| SPP1 | 1,086 | 0,005432435 |
| SPPL2A | 0,365 | 1 |
| SPRED1 | 0,522 | 1 |
| SPRY1 | 0,204 | 1 |
| SPRY4 | 2,504 | 1 |
| SPSB1 | 1,271 | 1 |
| SPTAN1 | 0,243 | 1 |
| SPTBN1 | 0,100 | 1 |
| SPTSSA | 0,416 | 1 |
| SPTY2D1 | 1,197 | 1 |
| SQLE | 2,034 | 1 |
| SRA1 | 0,345 | 1 |
| SRD5A3 | 2,664 | 1 |
| SREK1 | 0,148 | 1 |
| SRM | 0,842 | 1 |
| SRP54 | 1,240 | 1 |
| SRP72 | 0,170 | 1 |
| SRPK2 | 0,777 | 1 |
| SRPRB | 2,622 | 0,316011593 |
| SRPX2 | 4,778 | 1 |
| SRRT | 0,724 | 1 |
| SRSF2 | 0,315 | 1 |
| SRSF9 | 0,345 | 1 |
| SSFA2 | 0,864 | 0,763580892 |
| SSNA1 | 0,582 | 1 |
| SSR2 | 0,354 | 1 |
| SSR3 | 0,863 | 1 |
| SSR4 | 1,283 | 1 |
| SSRP1 | 2,252 | 0,101164387 |
| STAB1 | 1,329 | 1 |
| STARD13 | 1,379 | 1 |
| STARD3NL | 0,171 | 1 |
| STAT1 | 0,153 | 1 |
| STAT2 | 1,401 | 1 |
| STAU1 | 0,151 | 1 |
| STC1 | 6,629 | 0,102613309 |
| STK24 | 0,635 | 1 |
| STK25 | 0,565 | 1 |
| STK3 | 1,181 | 1 |
| STK38L | 1,534 | 1 |
| STK39 | 1,130 | 1 |
| STMN1 | 1,388 | 0,925286007 |
| STMP1 | 0,770 | 1 |
| STOML2 | 1,062 | 1 |
| STRAP | 0,159 | 1 |
| STRN3 | 0,632 | 1 |
| STT3B | 0,518 | 1 |
| STX18 | 0,723 | 1 |
| STX2 | 0,401 | 1 |
| SUB1 | 0,189 | 1 |
| SUGT1 | 0,500 | 1 |
| SUPT16H | 0,447 | 1 |
| SURF4 | 1,135 | 0,746155673 |
| SUZ12 | 0,328 | 1 |
| SYNCRIP | 0,788 | 1 |
| TAF13 | 1,617 | 1 |
| TAF1D | 0,489 | 1 |
| TAGLN2 | 1,286 | 0,000438468 |
| TALDO1 | 0,442 | 1 |
| TANK | 0,656 | 1 |
| TAOK1 | 0,379 | 1 |
| TAPBP | 0,766 | 1 |
| TARS | 0,923 | 1 |
| TAX1BP3 | 1,691 | 1 |
| TCAF1 | 0,691 | 1 |
| TCEAL9 | 0,658 | 1 |
| TCF4 | 0,250 | 1 |
| TCN2 | 2,813 | 0,982636283 |
| TCP1 | 0,583 | 1 |
| TDG | 0,309 | 1 |
| TEAD1 | 1,044 | 1 |
| TENT5A | 1,335 | 1 |
| TES | 0,480 | 1 |
| TFG | 0,384 | 1 |
| TFPI | 0,982 | 1 |
| TGFB1 | 1,252 | 1 |
| TGFB2 | 1,322 | 1 |
| TGFB3 | 2,068 | 1 |
| TGFBI | 4,904 | 3,26E-15 |
| TGIF1 | 1,215 | 1 |
| TGM2 | 2,523 | 1 |
| THAP9-AS1 | 1,494 | 1 |
| THBD | 3,014 | 1 |
| THBS1 | 1,957 | 0,494374 |
| THRAP3 | 0,371 | 1 |
| THY1 | 4,430 | 8,76E-20 |
| TIGAR | 1,138 | 1 |
| TIMM13 | 0,663 | 0,431017568 |
| TIMM17A | 0,645 | 1 |
| TIMP1 | 3,102 | 6,19E-13 |
| TIMP2 | 0,258 | 1 |
| TIPRL | 0,278 | 1 |
| TKT | 1,011 | 1 |
| TLE3 | 1,034 | 1 |
| TM4SF1 | 1,435 | 1 |
| TM9SF2 | 0,388 | 1 |
| TM9SF3 | 0,274 | 1 |
| TMBIM6 | 0,192 | 1 |
| TMCO1 | 0,755 | 1 |
| TMCO3 | 1,288 | 1 |
| TMED10 | 1,337 | 1 |
| TMED2 | 0,705 | 1 |
| TMED3 | 0,858 | 1 |
| TMED5 | 1,185 | 1 |
| TMED9 | 0,837 | 1 |
| TMEM123 | 0,394 | 1 |
| TMEM132A | 0,771 | 1 |
| TMEM141 | 0,363 | 1 |
| TMEM165 | 1,113 | 1 |
| TMEM167A | 0,722 | 1 |
| TMEM179B | 0,447 | 1 |
| TMEM208 | 0,144 | 1 |
| TMEM214 | 1,399 | 1 |
| TMEM230 | 0,221 | 1 |
| TMEM258 | 0,126 | 1 |
| TMEM263 | 1,005 | 1 |
| TMEM50A | 0,187 | 1 |
| TMEM70 | 1,113 | 1 |
| TMEM87B | 0,669 | 1 |
| TMEM8A | 0,726 | 1 |
| TMF1 | 0,480 | 0,871463482 |
| TMOD3 | 0,206 | 1 |
| TMSB10 | 1,629 | 2,64E-18 |
| TMTC3 | 0,451 | 1 |
| TMX1 | 1,332 | 1 |
| TMX3 | 1,275 | 1 |
| TNF | 1,618 | 1 |
| TNFAIP1 | 0,742 | 1 |
| TNFAIP3 | 1,303 | 0,195746447 |
| TNFAIP6 | 2,854 | 0,102613309 |
| TNFAIP8L1 | 0,802 | 1 |
| TNFRSF12A | 2,130 | 0,162560844 |
| TNFRSF1A | 0,918 | 1 |
| TNFRSF21 | 0,742 | 1 |
| TNIP1 | 1,276 | 1 |
| TNKS1BP1 | 0,278 | 1 |
| TNKS2 | 1,199 | 1 |
| TNPO1 | 0,616 | 1 |
| TNS3 | 0,861 | 1 |
| TOMM20 | 0,186 | 1 |
| TOMM22 | 0,998 | 1 |
| TOP1 | 0,342 | 1 |
| TOPBP1 | 1,416 | 1 |
| TP53 | 1,598 | 1 |
| TP53BP2 | 0,454 | 1 |
| TP53I3 | 0,212 | 1 |
| TPD52L2 | 1,022 | 1 |
| TPGS1 | 0,378 | 1 |
| TPI1 | 0,944 | 0,081849017 |
| TPM3 | 0,983 | 1 |
| TPM4 | 1,662 | 1,60E-07 |
| TPP1 | 0,627 | 1 |
| TPP2 | 0,601 | 1 |
| TPPP3 | 1,164 | 1 |
| TPRKB | 0,139 | 1 |
| TPX2 | 6,960 | 1 |
| TRAF4 | 1,321 | 1 |
| TRAF7 | 1,077 | 1 |
| TRAM1 | 1,153 | 1 |
| TRAM2 | 1,709 | 1 |
| TRIAP1 | 0,833 | 1 |
| TRIM24 | 1,234 | 1 |
| TRIM28 | 0,390 | 1 |
| TRIO | 1,348 | 1 |
| TRPC6 | 2,682 | 1 |
| TSC22D4 | 1,208 | 1 |
| TSG101 | 0,278 | 1 |
| TSPAN15 | 0,981 | 1 |
| TSPAN5 | 1,929 | 1 |
| TSPAN9 | 1,605 | 1 |
| TUBA1A | 0,385 | 1 |
| TUBA1B | 0,874 | 0,834615148 |
| TUBA1C | 3,073 | 2,89E-08 |
| TUBB | 2,198 | 1,09E-08 |
| TUBB4B | 0,650 | 1 |
| TUBB6 | 1,196 | 1 |
| TUFM | 0,323 | 1 |
| TUSC3 | 1,698 | 1 |
| TWF1 | 0,384 | 1 |
| TWIST1 | 3,054 | 0,661533913 |
| TWISTNB | 0,229 | 1 |
| TXLNA | 1,415 | 1 |
| TXN | 0,817 | 1 |
| TXN2 | 0,513 | 1 |
| TXNDC12 | 0,509 | 1 |
| TXNDC15 | 0,703 | 1 |
| TXNDC17 | 1,332 | 0,289908346 |
| TXNL1 | 0,856 | 1 |
| TXNRD1 | 1,799 | 1 |
| TYMP | 0,973 | 0,095401243 |
| TYMS | 1,974 | 1 |
| TYROBP | 0,596 | 1 |
| U2SURP | 0,320 | 1 |
| UACA | 2,413 | 0,000773163 |
| UBA1 | 0,354 | 1 |
| UBA5 | 1,289 | 1 |
| UBALD2 | 0,843 | 1 |
| UBAP2 | 1,237 | 1 |
| UBAP2L | 0,477 | 1 |
| UBE2A | 0,394 | 1 |
| UBE2B | 0,147 | 1 |
| UBE2D1 | 0,457 | 1 |
| UBE2D3 | 0,323 | 1 |
| UBE2E3 | 0,771 | 1 |
| UBE2I | 0,193 | 1 |
| UBE2J1 | 1,640 | 0,382248161 |
| UBE2J2 | 0,455 | 1 |
| UBE2L3 | 0,608 | 0,110046968 |
| UBE2N | 0,538 | 1 |
| UBE2Q2 | 0,197 | 1 |
| UBE2R2 | 0,352 | 1 |
| UBE2S | 1,864 | 0,321885549 |
| UBE2Z | 0,616 | 1 |
| UBQLN1 | 0,841 | 1 |
| UBR5 | 0,188 | 1 |
| UBXN4 | 0,117 | 1 |
| UFD1 | 0,705 | 1 |
| UFM1 | 0,849 | 1 |
| UGCG | 1,472 | 0,081443711 |
| UGGT1 | 0,752 | 1 |
| UNC5B | 1,452 | 1 |
| UPP1 | 1,375 | 1 |
| UQCC2 | 0,564 | 1 |
| UQCRFS1 | 0,440 | 1 |
| UQCRH | 0,415 | 1 |
| UROS | 0,746 | 1 |
| USO1 | 0,319 | 1 |
| USP14 | 1,096 | 1 |
| USP3 | 1,631 | 1 |
| USP48 | 0,840 | 1 |
| USP7 | 0,447 | 1 |
| USP9X | 0,917 | 1 |
| UXT | 0,468 | 1 |
| VAMP3 | 0,339 | 1 |
| VAMP8 | 0,905 | 1 |
| VAPA | 0,283 | 1 |
| VASP | 0,381 | 1 |
| VAT1 | 1,259 | 1 |
| VCAN | 5,476 | 0,000587608 |
| VCL | 0,444 | 1 |
| VCP | 0,990 | 1 |
| VDAC1 | 0,989 | 0,729973332 |
| VDAC3 | 0,357 | 1 |
| VEGFA | 1,504 | 1 |
| VEZT | 0,313 | 1 |
| VGLL4 | 0,222 | 1 |
| VIM | 1,914 | 1,16E-20 |
| VKORC1 | 0,595 | 1 |
| VMA21 | 0,442 | 1 |
| VMP1 | 1,041 | 1 |
| VPS26B | 0,788 | 1 |
| VPS29 | 0,410 | 1 |
| VPS35 | 0,411 | 1 |
| VPS41 | 0,942 | 1 |
| VPS4A | 0,604 | 1 |
| VSIG4 | 1,283 | 1 |
| VSTM4 | 0,636 | 1 |
| VTI1B | 0,333 | 1 |
| VWA1 | 3,285 | 1 |
| VWF | 2,274 | 1 |
| WAC | 0,347 | 1 |
| WDR1 | 0,565 | 1 |
| WDR41 | 0,551 | 1 |
| WDR60 | 0,946 | 1 |
| WDR83OS | 0,337 | 1 |
| WIPI1 | 1,098 | 1 |
| WSB2 | 0,445 | 1 |
| WTAP | 0,103 | 1 |
| WWTR1 | 0,640 | 0,093132614 |
| XBP1 | 0,608 | 1 |
| XIAP | 0,608 | 1 |
| XIST | 0,720 | 1 |
| XRCC5 | 0,141 | 1 |
| XRN2 | 0,914 | 1 |
| YARS | 1,331 | 1 |
| YBX1 | 0,718 | 0,002688145 |
| YES1 | 2,622 | 0,341751081 |
| YIF1A | 0,541 | 1 |
| YIF1B | 2,873 | 1 |
| YIPF5 | 0,436 | 1 |
| YRDC | 1,115 | 1 |
| YTHDF2 | 0,259 | 1 |
| YWHAE | 0,672 | 0,128345949 |
| YWHAG | 1,345 | 0,306694979 |
| YWHAH | 0,649 | 1 |
| YWHAZ | 0,747 | 1 |
| YY1 | 0,277 | 1 |
| ZBTB1 | 1,167 | 1 |
| ZBTB43 | 1,011 | 1 |
| ZC3H15 | 0,415 | 1 |
| ZC3HAV1 | 0,498 | 1 |
| ZCRB1 | 0,622 | 1 |
| ZDHHC20 | 1,227 | 1 |
| ZEB1 | 0,353 | 1 |
| ZEB2 | 0,196 | 1 |
| ZFAND5 | 0,161 | 1 |
| ZFAND6 | 0,562 | 1 |
| ZFAS1 | 0,384 | 1 |
| ZFX | 0,346 | 1 |
| ZFYVE16 | 0,835 | 1 |
| ZMIZ1 | 0,789 | 1 |
| ZMPSTE24 | 1,784 | 0,904842129 |
| ZNF207 | 0,489 | 1 |
| ZNF217 | 1,130 | 1 |
| ZNF281 | 1,324 | 1 |
| ZNF331 | 0,279 | 1 |
| ZNF503 | 0,971 | 1 |
| ZNF622 | 1,098 | 1 |
| ZNHIT1 | 0,684 | 1 |
| ZSWIM6 | 0,856 | 1 |
| ZYG11B | 1,357 | 1 |
| ZYX | 0,381 | 1 |

C) Table listing the 228 shared genes between mouse and human mural cells. DEGs in mouse tumor mural cells were compared to contralateral cells and cross-referenced with DEGs in human tumor mural cells versus non-malignant tissue.

|  |
| --- |
| Acin1 |
| Actn1 |
| Adamts1 |
| Afap1 |
| Ahsa1 |
| Akr1a1 |
| Ankrd10 |
| Ankrd11 |
| Ankrd40 |
| App |
| Arcn1 |
| Arf1 |
| Arf4 |
| Arhgef12 |
| Arid1a |
| Arl2bp |
| Arl4a |
| Arpc3 |
| Asah1 |
| Atl3 |
| Atp1a1 |
| Atp2b4 |
| Atp6v0b |
| Bach1 |
| Baz1b |
| Bclaf1 |
| Bhlhe40 |
| Bmpr2 |
| Calr |
| Calu |
| Canx |
| Capn2 |
| Capza2 |
| Ccar1 |
| Ccnl2 |
| Cct6a |
| Cd164 |
| Cdc37 |
| Cdk12 |
| Cep170 |
| Chd8 |
| Cltc |
| Cmtm6 |
| Col4a2 |
| Colgalt1 |
| Crispld2 |
| Crk |
| Ctnnb1 |
| Ctsa |
| Ctsb |
| Cux1 |
| Cxxc5 |
| Ddx21 |
| Ddx3x |
| Derl1 |
| Dhx9 |
| Dnaja1 |
| Dock10 |
| Dst |
| Dync1h1 |
| Dync1li2 |
| Eif4g1 |
| Eif4g2 |
| Eif5a |
| Eml4 |
| Eny2 |
| Eprs |
| Erbin |
| Errfi1 |
| Exoc5 |
| Fam13c |
| Fbxo32 |
| Fcho2 |
| Flt1 |
| Fosl2 |
| Frmd4b |
| Fus |
| Gadd45b |
| Glg1 |
| Gls |
| Glul |
| Glyr1 |
| Gnai2 |
| Gnb2 |
| Gns |
| Golga4 |
| Golph3 |
| Gpcpd1 |
| Hdlbp |
| Hip1 |
| Hnrnpc |
| Hnrnpf |
| Hnrnpm |
| Hspa8 |
| Hspd1 |
| Id1 |
| Id3 |
| Ier3 |
| Ifnar1 |
| Impdh2 |
| Irf1 |
| Jmjd1c |
| Kcnq1ot1 |
| Kdm6b |
| Kif1b |
| Klf4 |
| Lama4 |
| Lamp2 |
| Litaf |
| Lmna |
| Macf1 |
| Maff |
| Man1a2 |
| Map1lc3b |
| Map3k8 |
| Map4 |
| Map4k4 |
| Mapk1 |
| Mapre1 |
| Mgat1 |
| Mical2 |
| Midn |
| Mir22hg |
| Mlec |
| Morf4l2 |
| Mtus1 |
| Myo1e |
| Naa50 |
| Nars |
| Ncl |
| Neat1 |
| Nfic |
| Nfkbia |
| Nfkbiz |
| Nr4a2 |
| Nrip1 |
| Nrp1 |
| Nufip2 |
| Olfml2a |
| Osmr |
| P4ha1 |
| Pabpc1 |
| Pcbp1 |
| Pcm1 |
| Pde4b |
| Pdlim5 |
| Pds5a |
| Pdxk |
| Pim3 |
| Plekha1 |
| Plod1 |
| Plod2 |
| Pon2 |
| Ppp1cc |
| Ppp1r15a |
| Ppp1r2 |
| Ppp3ca |
| Prnp |
| Prrc2c |
| Psap |
| Psmd7 |
| Rab10 |
| Rab22a |
| Ralb |
| Ran |
| Rbbp4 |
| Rbm25 |
| Reep3 |
| Rhoa |
| Rhoc |
| Rnf19a |
| Rrbp1 |
| Rsrc2 |
| Runx1 |
| Sar1a |
| Scp2 |
| Sdcbp |
| Serbp1 |
| Sf3b2 |
| Sfpq |
| Slbp |
| Slc3a2 |
| Smchd1 |
| Smim10l1 |
| Snx17 |
| Snx3 |
| Socs3 |
| Spag9 |
| Sptan1 |
| Sptbn1 |
| Sptssa |
| Srek1 |
| Srpk2 |
| Srrt |
| Srsf2 |
| Stk38l |
| Taf1d |
| Thbs1 |
| Thrap3 |
| Tle3 |
| Tm9sf2 |
| Tm9sf3 |
| Tmed9 |
| Tmx3 |
| Tnfrsf1a |
| Tnpo1 |
| Tpm3 |
| Tpm4 |
| Tpp1 |
| Tsc22d4 |
| Txnrd1 |
| Uaca |
| Ubap2l |
| Ube2d3 |
| Ube2r2 |
| Usp14 |
| Usp7 |
| Usp9x |
| Vmp1 |
| Wdr1 |
| Ybx1 |
| Yy1 |
| Zeb2 |
| Zfand5 |
| Zfand6 |
| Zmiz1 |
| Zyg11b |
| Zyx |
